# Multi-Omics and Integrated Network Analyses Reveal New Insights into the Systems Relationships between Metabolites, Structural Genes, and Transcriptional Regulators in Developing Grape Berries (*Vitis vinifera* L.) Exposed to Water Deficit

**DOI:** 10.3389/fpls.2017.01124

**Published:** 2017-07-10

**Authors:** Stefania Savoi, Darren C. J. Wong, Asfaw Degu, Jose C. Herrera, Barbara Bucchetti, Enrico Peterlunger, Aaron Fait, Fulvio Mattivi, Simone D. Castellarin

**Affiliations:** ^1^Department of Agricultural, Food, Environmental and Animal Sciences, University of UdineUdine, Italy; ^2^Department of Food Quality and Nutrition, Research and Innovation Centre, Fondazione Edmund MachSan Michele all’Adige, Italy; ^3^Wine Research Centre, The University of British Columbia, VancouverBC, Canada; ^4^The Jacob Blaustein Institutes for Desert Research, Ben-Gurion University of the NegevSede Boqer, Israel; ^5^Center Agriculture Food Environment, University of TrentoSan Michele all’Adige, Italy

**Keywords:** abiotic stress, central metabolism, drought, grapevine, fruit quality, ripening, RNA-sequencing, specialized metabolism

## Abstract

Grapes are one of the major fruit crops and they are cultivated in many dry environments. This study comprehensively characterizes the metabolic response of grape berries exposed to water deficit at different developmental stages. Increases of proline, branched-chain amino acids, phenylpropanoids, anthocyanins, and free volatile organic compounds have been previously observed in grape berries exposed to water deficit. Integrating RNA-sequencing analysis of the transcriptome with large-scale analysis of central and specialized metabolites, we reveal that these increases occur via a coordinated regulation of key structural pathway genes. Water deficit-induced up-regulation of flavonoid genes is also coordinated with the down-regulation of many stilbene synthases and a consistent decrease in stilbenoid concentration. Water deficit activated both ABA-dependent and ABA-independent signal transduction pathways by modulating the expression of several transcription factors. Gene-gene and gene-metabolite network analyses showed that water deficit-responsive transcription factors such as bZIPs, AP2/ERFs, MYBs, and NACs are implicated in the regulation of stress-responsive metabolites. Enrichment of known and novel *cis*-regulatory elements in the promoters of several ripening-specific/water deficit-induced modules further affirms the involvement of a transcription factor cross-talk in the berry response to water deficit. Together, our integrated approaches show that water deficit-regulated gene modules are strongly linked to key fruit-quality metabolites and multiple signal transduction pathways may be critical to achieve a balance between the regulation of the stress-response and the berry ripening program. This study constitutes an invaluable resource for future discoveries and comparative studies, in grapes and other fruits, centered on reproductive tissue metabolism under abiotic stress.

## Introduction

Drought is considered one of the major threats for crops in the predicted future climatic scenarios. In fruit crops, drought can impact the accumulation of metabolites that determine fruit quality (Ripoll et al., 2014). Generally classified as drought tolerant, grape is often not irrigated or minimally irrigated to improve the berry composition (Chaves et al., 2007). Previous research showed that water deficit causes large changes on the specialized metabolism of the grape berry, promoting the synthesis of specific volatile organic compounds (Bindon et al., 2007; Savoi et al., 2016), carotenoids (Deluc et al., 2009), and phenolics (Castellarin et al., 2007a,b; Hochberg et al., 2015). The grape berry central metabolism is also affected by water deficit. Deluc et al. (2009) reported an increase of proline concentration in Cabernet Sauvignon berries, and the increase paralleled the induction of key genes for the proline synthesis such as the one codifying for the pyrroline-5-carboxylate synthase. Hochberg et al. (2015) reported that also other central metabolites, including sucrose, several amino acids and organic acids, ascorbate, and raffinose can take part in the metabolic response of the grape berry to water deficit.

These results indicate that a complex regulation of several metabolic pathways, possibly determined by common or specific molecular signals, underlay the metabolic response of the grape berry to water deficit. In the model plant *Arabidopsis thaliana*, the transcriptional response to drought is modulated by both ABA-dependent and ABA-independent signal transduction pathways (Yamaguchi-Shinozaki and Shinozaki, 2006; Singh and Laxmi, 2015). In the ABA dependent pathway, the accumulation of ABA is sensed by PYR/PYL/RCAR-PP2C receptor complex, and activates a class III SnRK2s that phosphorylates four transcription factors (TFs), ABA-responsive element (*ABRE*) binding protein 1 (*AREB1*), *AREB2*, *ABRE* binding factor 3 (*ABF3*), and *ABF1*. These TFs regulate several downstream genes by binding to the *ABRE cis*-regulatory element (CRE) present in the promoter region of these genes. Drought induced ABA also regulates the activity of MYB/MYC, NAC, WRKY, and NF-Y TFs. In the ABA-independent signaling pathway, the DREB2A, a protein that belongs to the AP2/ERF family, plays a pivotal role in the transcriptional response under drought stress (Yoshida et al., 2014). Recently, a cross-talk between ABA-dependent and ABA-independent pathways has been hypothesized under drought stress, with AREB/ABFs inducing DREB2A (Nakashima et al., 2014; Singh and Laxmi, 2015). In grape, ABA participates in the drought response of multiple organs and in the process of fruit ripening. In the xylem sap of shoots and roots, ABA levels negatively correlate with stem and root water potential, and increases of ABA concentration correspond to decreases of leaf transpiration (Rossdeutsch et al., 2016). ABA related genes were shown to be involved in the transcriptional response of the leaf to drought (Dal Santo et al., 2016; Hopper et al., 2016) and, in the grape berry, water deficit increased ABA concentration and ABA related transcripts, but not consistently between varieties (Deluc et al., 2009).

Among fruit crops, grapes are the most considered crop in studies on the impact of drought on fruit composition. The adoption of large-scale metabolite and transcript analyses (Deluc et al., 2009; Hochberg et al., 2015; Savoi et al., 2016) has strongly enhanced our understanding of the metabolic response of the grape berry to water deficit; however, systems approaches considering the analysis of primary and secondary metabolism, the molecular pathways, and the network relationships between genes and metabolites involved in the response to water deficit are still missing in grape, as well as in other fleshy fruits. Furthermore, the modulation of the volatile organic compounds (VOCs) under water deficit has been poorly investigated in wine grapes (Savoi et al., 2016), although they represent a key component of the final wine flavor.

Recent studies have successfully considered a multi-omics approach in several annual herbaceous crops, such as *Medicago truncatula* (Zhang et al., 2014), *Oryza sativa* (Maruyama et al., 2014), and *Zea mays* (Opitz et al., 2016). In these studies, RNA-sequencing and large-scale metabolite analyses were adopted and revealed several commonalities in the metabolic response between these crops and *A. thaliana*. Network-based analyses using multi-omics data provide a powerful tool for discovering links between and within the many layers of biological complexity that governs plant functions such as the coordinated regulation of genes and metabolic pathways. This approach has been performed in a few fruit crop studies to prioritize candidate genes involved the control of fruit development and composition (Mounet et al., 2009; Zamboni et al., 2010; Osorio et al., 2011, 2012; Savoi et al., 2016). We aimed to apply a similar approach to deeply characterize the molecular and metabolite response to water deficit in the grape berry, to uncover the relationships between water deficit-responsive genes and the metabolite accumulation during berry development, and to identify the key putative molecular regulators that underlay the metabolic response to water deficit. For that purpose, we conducted a two season experiment where two contrasting water regimes were applied and the levels of transcripts and metabolites were analyzed at several berry developmental stages. Transcripts and metabolites that were modulated by water deficit were used for constructing molecular networks to investigate their relationships and to identify the major molecular pathways that underlay the response of grape berry metabolism to this abiotic stress.

## Materials and Methods

### Field Experiment, Physiological Measurements, and Sample Preparation

Field experiments were conducted in 2011 and 2012 at the University of Udine experimental farm on 18/19 years old *Vitis vinifera* L. ‘Merlot’ (clone R3 onto SO4 rootstock) vines. The vines were planted with 2.5 m × 1.0 m spacing and trellised to a spur cordon system. To ensure a proper management of the water regime during the experimental trial and consistent treatments across seasons, four rows were covered with an ethylene-vinyl-acetate (EVA) film at the beginning of the seasons, as described in Herrera et al. (2015). Two irrigation treatments were imposed at approximately 25 days after anthesis (DAA): control (CT) vines where weekly irrigated maintaining the midday stem water potential (Ψ_Stem_) above -0.6 MPa and water deficit (WD) vines were not irrigated from fruit set until Ψ_Stem_ was lower than -1.4 MPa, whereupon irrigation was managed to maintain Ψ_Stem_ between -1.0 and -1.4 MPa. Each treatment was replicated four times in plots of 10 vines each in a completely randomized design. Plant water status was monitored weekly by measuring midday Ψ_Stem_ (Savoi et al., 2016).

Berries were randomly sampled for analyses seven times during each season: three times before the beginning of ripening (30, 44, and 60 DAA in 2011, and 26, 40, and 53 DAA in 2012), at the beginning of ripening (74 DAA in 2011 and 67 DAA in 2012), and three times during berry ripening (87, 100, and 115 DAA in 2011, and 81, 95, and 106 DAA in 2012). These developmental stages corresponded to E-L 31, 32, 33, 35, 36, 37, and 38 in the E-L system, respectively. The onset of ripening (veraison, defined as the day at which 50% of the berries had changed color from green to red) was recorded at 69 and 60 DAA in 2011 and 2012, respectively. In both seasons, the last sampling point coincided with the harvest date.

At each date, two sets of berries were randomly collected from each plot, for a total of four biological replicates per irrigation treatment. The first set of 60 berries was used for measuring berry weight, total soluble solids (TSS), titratable acidity (TA), and pH as described in Herrera et al. (2015). The second set of 40 berries was used for the metabolite and transcript analyses; samples were snap frozen with liquid nitrogen, and stored at -80°C.

For metabolite and transcript analyses, whole berries, without pedicel, were grinded to a fine powder under liquid nitrogen using an analytical mill (IKA-Werke GMbH & Co.). The frozen powder was aliquoted for metabolite and RNA extraction as described below. Moreover, a quality control (QC) sample for metabolite analysis was prepared by pooling together aliquots of all the samples. Large-scale targeted metabolite analyses were undertaken in both seasons, while transcriptome analyses were performed only in 2012.

### Metabolite Analyses

Primary metabolites were extracted from 100 mg of frozen powder, derivatized for GC-MS analysis and analyzed in a Trace GC Ultra gas chromatograph coupled to a DSQII quadrupole mass spectrometer (Thermo Scientific) as described in Degu et al. (2014). XCalibur software was used for the mass spectra identification using the NIST library (United States) and the RI libraries from the Max-Planck Institute for Plant Physiology (Germany). The QC sample was used for data normalization.

Metabolites were determined as described in Savoi et al. (2016) unless specified. Phenylpropanoids, stilbenoids, flavonols, flavan-3-ols, and proanthocyanidins chromatographic analysis was carried out using a Waters Acquity UPLC system (Milford) coupled to a Waters Xevo triple-quadrupole mass spectrometer detector (Milford). Compounds were identified with TargetLynx software based on their reference standard, retention time, and qualifier and quantifier ion, and were accurately quantified by their calibration curve and expressed as mg/Kg of grapes. Anthocyanins were analyzed as described in Sivilotti et al. (2016) using a HPLC (Shimadzu) equipped with a diode array detector. The concentration of individual anthocyanins was expressed in oenin chloride equivalents and expressed as mg/Kg of grapes. Carotenoids chromatographic analysis was performed in a 1290 Infinity Binary UPLC (Agilent) equipped with an RP C30 3 μm column Spectra components and elution profiles were determined with the R package ‘alsace’ 3.0. Compounds were quantified from linear calibration curves built with standard solutions and expressed as mg/Kg of grapes.

Free (non-glycosylated) VOCs analysis was performed with a Trace GC Ultra gas chromatograph (Thermo Scientific) coupled to a TSQ Quantum Tandem mass spectrometer. XCalibur software was used for the peaks identification. VOCs were identified by comparing the retention times of individual peaks with the retention times of their reference standards, and by identifying the mass spectra using the NIST library. The ratio of each VOC area to the d_8_-acetophenone internal standard area was considered to reduce technical variability among extractions and chromatographic runs and VOCs quantity were expressed as μg/Kg of grapes of d_8_-acetophenone equivalents.

Extractions and injections of the samples were performed in a random sequence and QC samples were injected at the beginning of the sequence and every six sample injections.

### RNA Extractions and RNA Sequencing Analysis

Transcriptome analyses were performed on the samples collected at 26, 53, 67, 81, and 106 DAA in 2012. Three out of the four biological replicates per treatment were considered. RNA extraction, RNA quality and quantity determination, library preparation and quantification, sequencing and QC analysis were performed as described in Savoi et al. (2016). Reads were aligned against the reference grape genome V1 PN40024 12X (Jaillon et al., 2007) using the software TopHat version 2.0.6 (Trapnell et al., 2012) with default parameters. Aligned reads were counted with htseq-count (version 0.6.0), in intersection-non-empty mode for overlap resolution (Anders et al., 2015). Differentially expressed (DE) genes [false discovery rate (FDR) less than 0.05] analysis was performed with the R package ‘DESeq2’ (Love et al., 2014). Annotation of gene functions was done according to Grimplet et al. (2012) and Naithani et al. (2014) and retrieved from recent literature. Gene ontology analyses for each sampling were carried out as described in Savoi et al. (2016).

### Quantitative Real-Time Polymerase Chain Reaction

The validation of RNA-Seq data was carried out on a set of DE genes using the quantitative real-time polymerase chain reaction (qPCR) technique. The reverse transcription of RNA samples was performed with the QuantiTect Reverse Transcription Kit (Qiagen); specific primers for 12 selected genes were designed with Primer3web version 4.0.0 (**Supplementary Table S1**). Real-Time PCR experiments were performed on a Bio-Rad CFX96TM using SsoFastTM EvaGreen^®^ Supermix. qPCR run condition were as per instructions with annealing temperature of 58°C. *VviAP47* (*VIT_02s0012g00910*) was used as a reference gene (Reid et al., 2006). In order to validate the technical and biological reliability of the transcriptome dataset, qPCR analysis was carried out on samples collected at different stages of development (starting from the sampling that preceded the onset of ripening) in 2011 and 2012.

### Statistical and Network Analyses

A one-way ANOVA was performed using JMP 7 (SAS Institute Inc.) to detect significant differences (*P* < 0.05) between irrigation treatments at each sampling date for the several physiological and compositional parameters considered. Heatmaps representing the log_2_ fold change (log_2_FC) of metabolite concentrations between treatments (WD/CT) were drawn with R software. Metabolites were clustered with Person correlation and a complete link. Principal component analyses (PCAs) on metabolite and transcriptome datasets were performed using the R software. Co-expression analysis was performed using weighted correlation gene co-expression network analysis (WGCNA) package in R (Langfelder and Horvath, 2008) using the log_2_ fold change (WD/CT) [dataset1] and the variance stabilized transformed (VST) counts [dataset2] of DE genes at 26, 53, 67, 81, and 106 DAA, separately to identify highly correlated genes sharing similar water deficit co-response and development accumulation patterns, respectively. Empirical *P*-value of Pearson Correlation Coefficient (PCC) values (statistical significance) of dataset1 and dataset2 were estimated by 1,000 permutations using the ‘rsgcc’ package (Ma and Wang, 2012). Enrichment for CRE in the gene promoters (1 kb upstream of the 5′ UTR or TSS) of each WGCNA co-response modules was conducted as described previously (Savoi et al., 2016; Wong et al., 2016). Known CREs of 6-, 7-, 8-mers (222 in total) were analyzed. Enrichment of CREs was validated with the hypergeometric test adjusted with FDR correction. Putative CREs were deemed significantly enriched under FDR < 0.01. Stress co-response and development-regulated submodules were created using PCC threshold > 0.8 with an empirical *P*-value < 0.01 and visualized using Cytoscape software (version 3.1.1) (Shannon et al., 2003).

### Accession Number

All raw sequence reads have been deposited in NCBI Sequence Read Archive^1^. The BioProject accession is PRJNA348618.

## Results

### Impact of Water Deficit on Fruit Development

**FIGURE 1:**
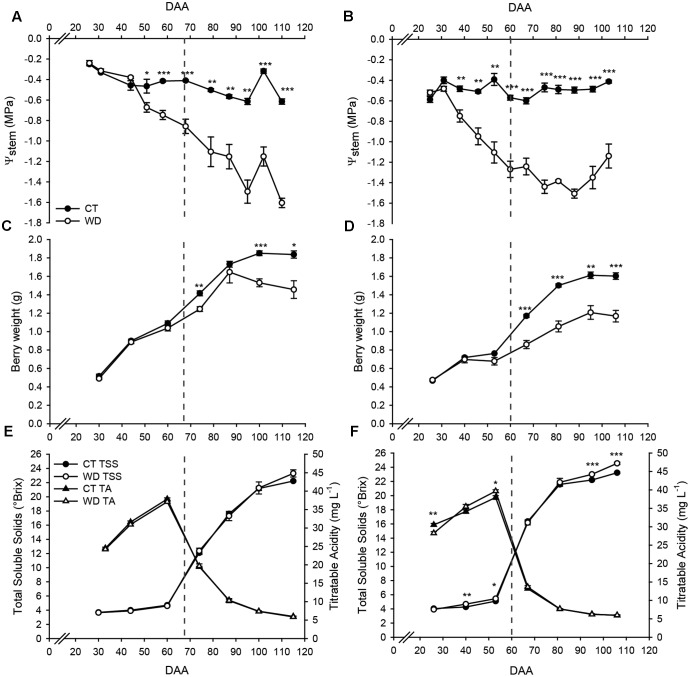
Stem water potential and physiological parameters of control (CT) and minimally irrigated (WD) vines and grapes in 2011 (left panels) and 2012 (right panels). **(A,B)** Stem water potential (Ψ_Stem_), **(C,D)** berry weight, **(E,F)** total soluble solids and titratable acidity. Dashed line indicates the onset of ripening (veraison). Bars represent ± SE. Asterisks indicate significant differences between treatments at *P* < 0.05 (^∗^), *P* < 0.01 (^∗∗^), *P* < 0.001 (^∗∗∗^) evaluated by one-way ANOVA.

In 2011, differences in Ψ_Stem_ between treatments started from 50 DAA and the lowest values in WD vines (<-1.4 MPa) were measured at late stages of fruit development (95 and 110 DAA). Whereas in 2012, differences between the treatments started 10 days earlier (39 DAA), and the lowest values in WD vines (<-1.4 MPa) were measured at 75 and 88 DAA (**Figure 1**). At this stage, the Ψ_Stem_ in WD vines was -0.8 and -1.2 MPa in 2011 and in 2012, respectively. In both seasons, Ψ_Stem_ of WD vines was consistently lower than -1.0 MPa for the entire ripening period.

Water deficit significantly reduced berry weight at 74, 100, and 115 DAA in 2011 (**Figure 1**) and at 67, 81, 95, and 106 DAA in 2012 (**Figure 1**). The reduction in berry weight was also reflected in lower vine productivity in both seasons (**Supplementary Table S2**).

Significant differences in TSS and TA were observed only in 2012 (**Figure 1**). In this season, water deficit increased TSS before (40 and 53 DAA) and after (95 and 106 DAA) the onset of fruit ripening, while TA was observed to be lower and higher in WD than in CT at 26 and 53 DAA, respectively.

### Impact of Water Deficit on Berry Metabolites

A total number of 101 compounds, belonging to the central (primary) and specialized (secondary) metabolism, were identified (standard annotation level 1) and quantified. A complete list of the compounds identified, with their machine readable chemical identifier, is reported in **Supplementary Table S3**, and the kinetics of the accumulation of these compounds in CT and WD berries during development in both vintages is reported in Supplementary Figure S1.

**FIGURE 2:**
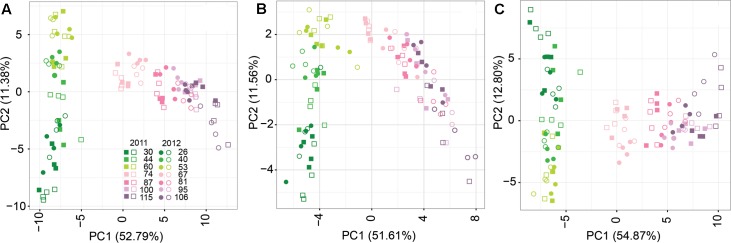
Principal component analysis (PCA) of CT (full symbols) and WD (open symbols) berry metabolite profiles in 2011 (squares) and 2012 (dots). **(A)** Whole set of metabolites; **(B)** Central metabolites; **(C)** Specialized metabolites. Colors from dark green to light pink to violet refer to developmental stages (DAA) of the berry as reported in the figure legend.

Principal component analyses were used to compare the metabolite profiles of CT and WD berries during development in both seasons considering the whole set of metabolites and the central and specialized metabolism separately (**Figure 2**). The first two principal components represent from 64.17 to 67.67% of the variance in the datasets. In all the PC1-PC2 score plots, clear separation among samples was observed based on the berry developmental stage. This separation was consistent between the two seasons; samples collected at similar developmental stages in the two seasons clustered together in the PC1-PC2 score plots. The PC2 and PC1 separated the developmental stages before and after the beginning of fruit ripening, respectively. Separation between irrigation treatments was observed from 100 DAA in 2011 and from 81 DAA in 2012. This large and coordinated change in the whole composition of the berry was more evident when the whole set of metabolites was included in the analysis than when central and specialized metabolism were analyzed independently (**Figure 2**). In both seasons, differences in the berry metabolite profiling between CT and WD berries were maximized at the last sampling stage.

**FIGURE 3:**
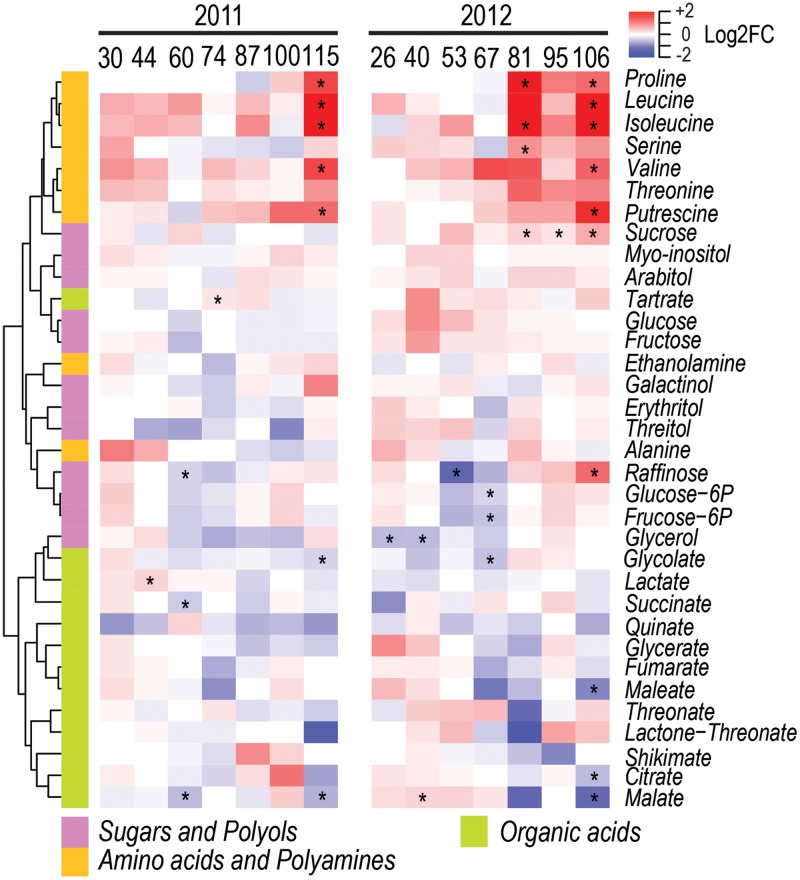
Impact of water deficit on central metabolites during berry development in 2011 (left) and 2012 (right). Heatmaps represent log_2_FC (WD/CT) at each developmental stage. Blue and red color shades indicate lower and higher metabolite concentration in WD than CT, respectively. Asterisks indicate significant differences (*P* < 0.05) between treatments. Metabolites were hierarchically clustered based on their response to water deficit. The colored side bar on the left indicates the class of metabolites.

Among the 34 central – amino acids, sugars, organic acids, polyols, and polyamines – metabolites analyzed, a total of 11 and 15 metabolites were significantly modulated by water deficit at one or more developmental stages in 2011 and 2012, respectively (**Figure 3** and Supplementary Figure S1). Most differences were observed during berry ripening, when in WD berries the level of leucine, valine, isoleucine, threonine, proline, and putrescine increased consistently in the two seasons. On the contrary, raffinose decreased before the beginning of ripening in both seasons as a result of water deficit, as well as malate decreased at late stages of ripening.

**FIGURE 4:**
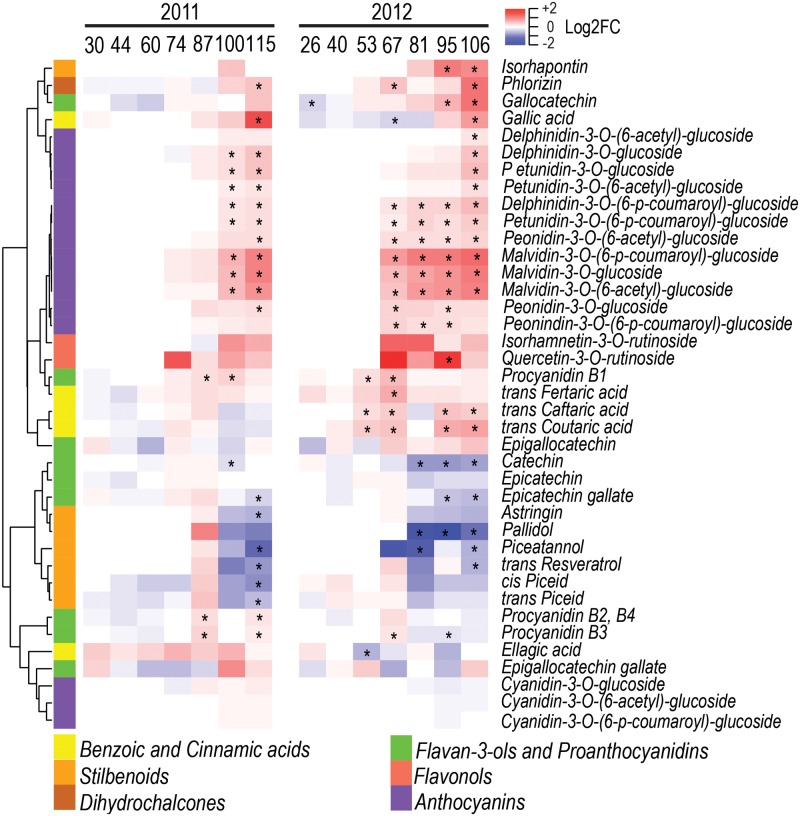
Impact of water deficit on phenolics during berry development in 2011 (left) and 2012 (right). Heatmaps represent log_2_FC (WD/CT) at each developmental stage. Blue and red color shades indicate lower and higher metabolite concentration in WD than CT, respectively. Asterisks indicate significant differences (*P* < 0.05) between treatments. Metabolites were hierarchically clustered based on their response to water deficit. The colored side bar on the left indicates the class of metabolites.

The specialized metabolites analyzed in this study were phenolics, carotenoids, and free VOCs. Among the 39 phenolics detected, 22 and 28 were significantly modulated by water deficit in 2011 and 2012, respectively (**Figure 4** and Supplementary Figure S1). Eighteen of them were consistently modulated between the two seasons. Water deficit increased the concentration of benzoic and cinnamic acids. Specifically, gallic acid was increased by water deficit at harvest in both seasons, while *trans*-caftaric, *trans*-coutaric, and *trans*-fertaric acids were increased only in 2012. Water deficit strongly increased the concentration of most anthocyanins detected. Stilbenoids, such as *trans*-resveratrol, piceatannol, and pallidol, were decreased by water deficit in both seasons at harvest, while *cis*- and *trans*-piceid were significantly decreased by water deficit only in 2011 even though they had a similar trend but showed no significance in 2012. Monomeric flavan-3-ols, such as catechin and epicatechin gallate, were decreased by water deficit in both seasons during berry ripening. Conversely, proanthocyanidins B1, B2+B4 were generally increased by water deficit. Flavonols were not consistently affected by water deficit; quercetin-3-*O*-rutinoside was significantly increased by water deficit, but only at 95 DAA in 2012. However, the method used for detecting and quantifying flavonols was unable to detect the glycosylated myricetin, one of the main flavonols produced in red grapes (Mattivi et al., 2006).

**FIGURE 5:**
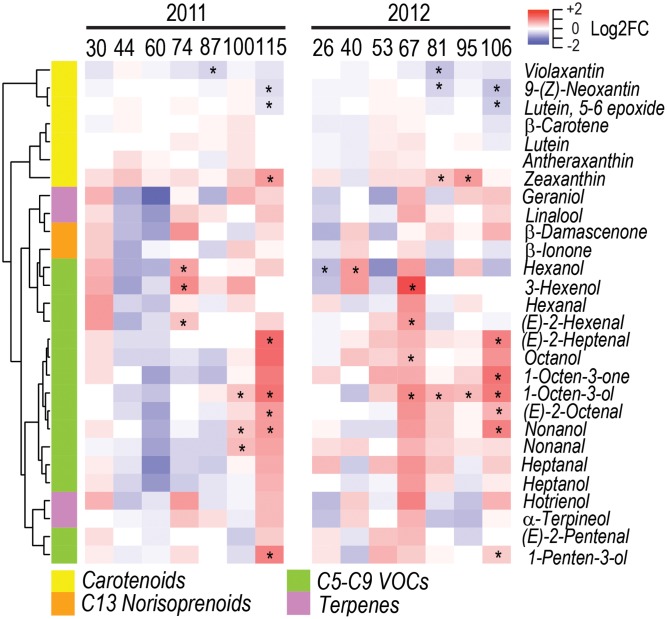
Impact of water deficit on carotenoids and free volatile organic compounds during fruit development in 2011 (left) and 2012 (right). Heatmaps represent log_2_FC (WD/CT) at each developmental stage. Blue and red color shades indicate lower and higher metabolite concentration in WD than CT, respectively. Asterisks indicate significant differences (*P* < 0.05) between treatments. Metabolites were hierarchically clustered based on their response to water deficit. The colored side bar on the left indicates the class of metabolites.

Carotenoids such as violaxanthin, neoxanthin, and lutein 5-6-epoxide, decreased in WD berries during ripening, while zeaxanthin increased (**Figure 5** and Supplementary Figure S1).

A strong increase of free VOCs was consistently observed between the two seasons at late developmental stages under water deficit. Remarkably, eight free VOCs were consistently modulated between seasons by water deficit (**Figure 5** and Supplementary Figure S1). C6 VOCs, such as *(E)-*2-hexenal and 3-hexenol, increased in WD berries at the onset of ripening, while the C6 VOC hexanol increased at 74 DAA in 2011 and at 40 DAA in 2012, but decreased at 26 DAA in 2012. Furthermore, C5, C7, C8, and C9 VOCs, such as 1-penten-3-ol, *(E)-*2-heptenal, *(E)-*2-octenal, 1-octen-3-ol, and nonanol increased in WD berries at late ripening stages.

### Impact of Water Deficit on Berry Transcriptome

Larger metabolic changes occurred in 2012, when water deficit started earlier and was overall more severe during the season (**Figure 1**), thus transcriptome analysis was undertaken on berry samples collected during this season at five berry developmental stages (before the onset of ripening, 26 and 53 DAA; at the onset of ripening, 67 DAA; and during ripening, 81 and 106 DAA).

The average number of unique reads that mapped the 12X V1 version of the grape genome (Jaillon et al., 2007) was 27.1 M (**Supplementary Table S4**). Among the 29,971 genes of the grape genome, 23,253 (77.6%) genes were expressed at 26 DAA, 23,220 (77.5%) at 53 DAA, 21,997 (73.4%) at 67 DAA, 22,453 (74.9%) at 81 DAA, and 22,162 (73.9%) at 106 DAA.

**FIGURE 6:**
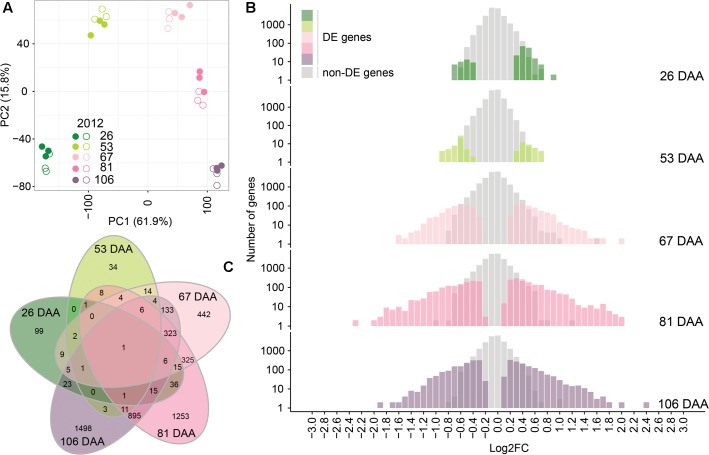
Analysis of CT and WD berry transcriptome in 2012. **(A)** PCA of the transcriptome of the 30 independent samples analyzed. Full and open symbols identify CT and WD berries, respectively. Colors from dark green to light pink to violet indicate the developmental stage (DAA) as reported in the figure legend. **(B)** Number of differently expressed (DE) genes and their log_2_FC (WD/CT) at 26, 53, 67, 81, and 106 DAA. **(C)** Commonly and uniquely DE genes at 26, 53, 67, 81, and 106 DAA represented in a Venn diagram.

A PCA was performed to compare the transcriptome profiles of the 30 independent samples analyzed (2 treatments × 5 developmental stages × 3 biological replicates) (**Figure 6**). The first two principal components explain 61.9 and 15.8%, of the variance among samples, respectively. Berry transcriptome were clearly separated accordingly to the developmental stage; moreover, within developmental stages, the berry transcriptomes of WD berries grouped together and were separated from the transcriptomes of CT berries.

The total number of DE genes between CT and WD was 5,167 (**Supplementary Table S5**). Water deficit modulated the expression of 214 genes (175 up-regulated; 39 down-regulated) at 26 DAA, 90 genes (38 up-regulated; 52 down-regulated) at 53 DAA, 1,290 genes (662 up-regulated; 628 down-regulated) at 67 DAA, 2,900 genes (1,569 up-regulated; 1,331 down-regulated) at 81 DAA, and 2,925 genes (1,431 up-regulated; 1,494 down-regulated) at 106 DAA (**Figure 6**). Several of them were differentially regulated at more than one developmental stage (**Figure 6**). Thirty GO categories (slim biological processes) were significantly overrepresented among the DE genes as presented in Supplementary Figure S2.

The expression of 12 selected DE genes was tested at several developmental stages in both seasons with a qPCR (Supplementary Figure S3). This analysis indicates that differences in the gene expression level between treatments remained consistent regardless the platform or the season considered.

### Transcriptional Regulatory Networks of Berries under Water Deficit

**FIGURE 7:**
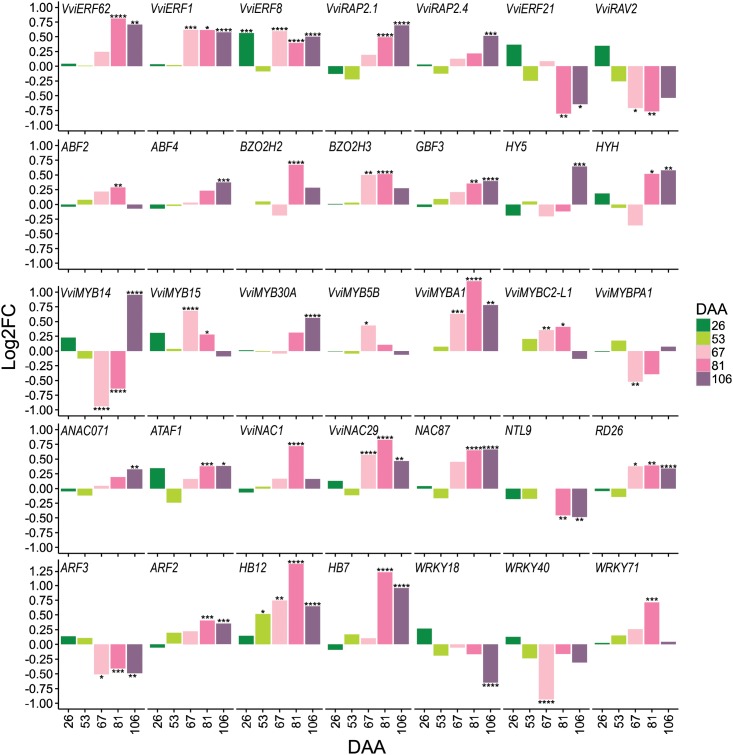
Selected transcription factors (TFs) differentially expressed in berries exposed to water deficit. Bar plots represent the gene expression log_2_FC (WD/CT) at 26, 53, 67, 81, and 106 DAA. The names of TFs are reported as their closest homologs in *Arabidopsis* or according to their original name when characterized in grape. Correspondence between grape and *Arabidopsis* nomenclature and putative function is reported in **Supplementary Table S5**. Colors from dark green to light pink to violet refer to developmental stages (DAA) of the berry as reported in the figure legend. Symbols indicate significant differential expression identified using DESeq2: *p*adj < 0.05 (^∗^), *p*adj < 0.01 (^∗∗^), *p*adj < 0.001 (^∗∗∗^), and *p*adj < 0.0001 (^∗∗∗∗^).

Transcription factors are central in regulating many plant biological processes; including developmental processes and response to the environment. A total of 447 TFs out of 2,211 possible TFs encoded in the grape genome (Grimplet et al., 2012) were modulated in the berry in response to water deficit. In this study, emphasis was given on the ripening stages (67, 81, and 106 DAA), when large metabolite and transcriptome responses to water deficit occurred. A large proportion of the water deficit-modulated TFs belongs to the MYB (33 genes), bHLH (33 genes), AP2-ERF (28 genes), C2H2 (27 genes), WRKY (26 genes), NAC (23 genes), C3H (20 genes), HB (18 genes), GRAS (16 genes), and bZIP (14 genes) families (**Supplementary Table S5**). A selection of these genes is reported in **Figure 7**.

Central components of the ABA-independent drought response pathway are the AP2/ERF TFs encoding dehydration-responsive element binding proteins (DREBs) that bind the dehydration-responsive element/C-repeat (DRE/CRT) sequence (Mizoi et al., 2012). Several of these genes, were significantly modulated by water deficit. For example, *VviERF1*, an upstream component of the jasmonic acid and ethylene signaling pathway, induced by high salinity and drought stress in *Arabidopsis* (Cheng et al., 2013), was strongly up-regulated by water deficit during fruit ripening. Another AP2/ERF-DREB (*VviRAP2.4*) was up-regulated by water deficit at late ripening. In *Arabidopsis* this TF confers drought tolerance by activating drought-responsive genes (Lin et al., 2008).

Our data indicate that basic leucine zipper (bZIP) proteins – components of the ABA-dependent signaling pathway – might also be implicated in the regulation of the drought response in fruits.

MYB TFs are key modulators of plant metabolism and development, and have been shown to be involved in the drought response. Several MYB TFs were strongly modulated by water deficit. Among them, we noted *VviMYB14* (*VIT_07s0005g03340*) and *VviMYB15* (*VIT_05s0049g01020)*, *VviMYB5B* (*VIT_06s0004g00570*), *VviMYBA1* (*VIT_02s0033g00410*), *VviMYBC2-L1* (*VIT_01s0011g04760*), and *VviMYBPA1* (*VIT_15s0046g00170*) that are involved in regulating various branches of the phenylpropanoid, stilbenoid, and flavonoid metabolism.

In this study, most of the DE NACs were up-regulated by water deficit. Among the other TFs modulated by water deficit, two auxin response factors (*VviARF3* – *VIT_10s0003g00420*; *VviARF2* – *VIT_17s0000g00320*) – possibly implicated in fruit development and ripening (Kumar et al., 2014) – encoding orthologous genes for *AtARF3/ETTIN* and *AtARF2*, were down- and up-regulated during ripening, respectively. Example of grape ARFs that may integrate multiple signaling pathways includes, *VviARF2* (*VIT_17s0000g00320*), the homolog of tomato *ARF2*, and *VviARF5* (*MONOPTEROS*, *VIT_18s0001g13930*). *Arabidopsis ARF2* and *ARF5* are master regulators of auxin hormone responses, largely targeting the genes implicated in hormone-mediated signaling pathway, growth, and tissues development, among others (O’Malley et al., 2016).

Two homeobox-leucine zipper proteins (*VviHB7* – *VIT_15s0048g02870*, and *VviHB12* – *VIT_16s0098g01170*), encoding for orthologous genes of *AtHB7* and *AtHB12* (Valdés et al., 2012), were significantly up-regulated under water deficit. Particularly, *VviHB12* was up-regulated from 53 DAA onward, with the highest induction at 81 DAA when water deficit reached the highest severity. Also, two of the WRKYs (*VviWRK18* – *VIT_04s0008g05760*, and *VviWRKY40* – *VIT_09s0018g00240*) involved in the ABA signaling pathway (Geilen and Böhmer, 2015) were down-regulated under water deficit during ripening. Vice versa, one WRKY TF (*VviWRKY71* – *VIT_12s0028g00270*), that has been shown to be involved in the oxidative stress process, as well as in the salicylic acid and jasmonic acid signaling pathways (Guo and Qin, 2016), was up-regulated at 81 DAA by water deficit.

### Modulation of Central and Specialized Pathway Genes under Water Deficit

Many genes DE under water deficit codify for enzyme involved in major central and specialized pathways. Detailed description of how these major pathways were modulated is presented and discussed in Supplementary Figures S4–S7.

Water deficit affected the expression of genes involved in 9 steps out of 10 of the glycolysis metabolic pathway (Supplementary Figure S4). Significant differences in the gene expression were observed from 67 DAA onward, and the majority of these genes were up-regulated by water deficit. Few genes of the TCA cycle were moderately modulated at 81 or 106 DAA. Moreover, several sugar transporters, possibly involved in the monosaccharide, sucrose, and polyol and hexose intake into the berry cells were modulated. Hexoses are the major sugars accumulated in the grape berry during ripening and, interestingly, most of the hexose transporters were down-regulated.

Water deficit increased the concentration of the short branched-chain amino acids leucine, valine, and isoleucine in both seasons (**Figure 3**) and several genes involved in valine and leucine biosynthesis were modulated during berry ripening (Supplementary Figure S5). Genes that underlay the synthesis of proline including glutamate dehydrogenases (*GluDH*), glutamate synthases (*GluS*), and a pyrroline-5-carboxylate synthase (*P5CS*) were strongly up-regulated during the final stages of ripening. Furthermore, genes that promote the decarboxylation of arginine or ornithine into polyamines were up-regulated.

Consistently with the differences observed in the phenylpropanoid, stilbenoid, and flavonoid accumulation (**Figure 4**), Water deficit strongly modulated most steps of the related biosynthetic pathways (Supplementary Figure S6); it promoted the expression of the branch of the flavonoid pathway which leads to the production of tri-hydroxylated anthocyanins (Castellarin et al., 2007b) and down-regulated 28 out of 45 stilbene synthases (*STSs*).

Water deficit also affected the expression of several genes of the carotenoid pathway (Supplementary Figure S7), mostly by up-regulating them. Carotenoids such as neoxanthin and violaxanthin can be cleaved by 9-*cis*-epoxycarotenoid dioxygenase (NCED) and further modified to produce the drought and ripening related hormone ABA. Three *VviNCEDs* were up-regulated in WD berries during ripening.

The molecular pathways that underlay the VOC production in fruits, as well as their modulation under water deficit remain largely unknown. Several VOCs detected in this study are produced from the peroxidation of free C18 polyunsaturated fatty acids, such as linolenic and linoleic acids, which lead to the production of C6, C9 (Kalua and Boss, 2009) and putatively C5 (Shen et al., 2014) VOCs. The fatty acid degradation involves lipoxygenases (LOX), hydroperoxide lyase (HPL), hexenal isomerases (HI), and alcohol dehydrogenases (ADH) (Schwab et al., 2008). We found that a 13-LOX, a HPL and five ADHs were up-regulated in WD berries during berry ripening. The induction of *VviLOX* (*VIT_06s0004g01510*) in WD berries at 106 DAA, may explain the higher accumulation of C5, C8, and C9 VOCs observed. In addition, we identified two grape genes codifying for *(Z)-*3:*(E)-*2-hexenal isomerases, recently identified paprika and tomato (Kunishima et al., 2016), consistently up-regulated in WD berries from 67 DAA onward.

Finally, we observed a strong modulation of many transcripts involved in the reactive oxygen species (ROS) related pathways (production, scavenging, and signaling) involved in the plant responses to abiotic stresses (**Supplementary Table S5**) (Dal Santo et al., 2016).

### Predicted Water Deficit-Regulated Modules Link Central Players in the Metabolic Response

**FIGURE 8:**
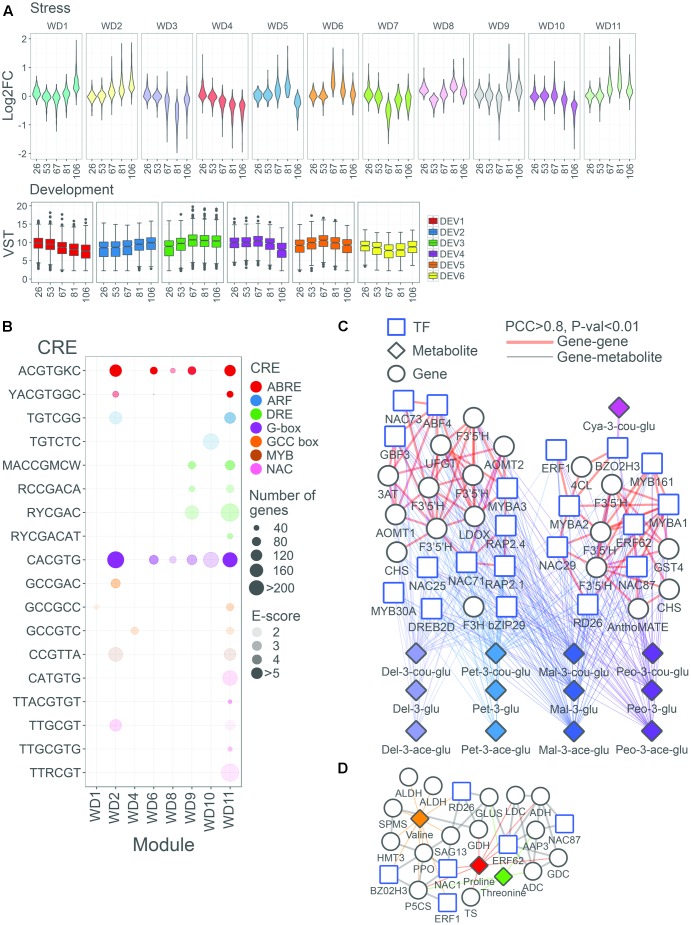
Functional overview of biological modules modulated by water deficit in the grape berry. **(A)** Violin plots represent the distribution of log_2_FC (WD/CT) of all DE genes in stress (WD) co-response gene modules. Box plots represent the distribution of variance stabilized transformed expression values of DE genes in development (DEV) co-regulated gene modules. **(B)** Overview of selected enriched *cis*-regulatory elements (CREs) in stress co-response genes modules. Colors of circles representative the TF family that recognize a designated CRE. Sizes or circles represent the number of genes containing a designated CRE in their promoter region. Opacity of circles (in color) represent the enrichment score, –log_10_(FDR), of CREs. Only enriched CREs (FDR < 0.01) in designated modules are depicted. Network representation of WD2 and WD11 modules, having ripening-associated expression patterns (DEV2/3/5), centered on significantly correlated TF and/or structural genes with **(C)** anthocyanin (pink, lavender blue, cyan, blue, and purple), and **(D)** amino acid compounds (orange, red, and green). Structural genes, TF, and metabolites are represented by circle, square, and triangle nodes, respectively. Thick edges in light red and gray edges represent associations between genes/TFs (PCC > 0.8; *P* < 0.01) for **(C,D)**, respectively. Gene-metabolite (PCC > 0.8; *P* < 0.01) are depicted in thinner edges and the different colors denote different categories, as reported in **(C,D)**.

To determine the correlation pattern among DE genes and analyze their regulation during berry development and in response to water deficit, WGCNA was performed (Langfelder and Horvath, 2008). Eleven co-response gene modules (clusters) of highly correlated genes based on the water deficit-modulation were identified, with each module containing up to six generalized development-based accumulation patterns (**Figure 8** and **Supplementary Table S6**). In seven modules (named WD1, 2, 5, 6, 8, 9, and 11) we observed a general up-regulation of the gene expression under water deficit; on the contrary, in four modules (named WD3, 4, 7, and 10) we observed a general down-regulation of the gene expression. Based on the fact that water deficit was stronger on metabolite and transcript abundance during fruit ripening, we focused on the DE that decreased in expression during ripening (sub-module DEV1 and DEV4), the ones that peaked at the onset of ripening (sub-module DEV5), the ones which were generally highly expressed from the onset of ripening to harvest (sub-module DEV3), and the ones which showed a steady increase in transcripts during ripening before peaking at harvest (sub-module DEV2).

In order to gain more insights into the regulatory control of water deficit-induced genes, the promoters of module genes were evaluated for CRE enrichment (**Figure 8** and **Supplementary Table S6**). We evaluated 222 CREs, 6- to 8-mer in length, given that these lengths usually define the primary DNA element recognized by various plant TFs (Franco-Zorrilla et al., 2014). We have recently shown that, in grape, many of these CREs are indeed bona-fide CREs with large biological relevance (Wong et al., 2016, 2017). The ACGTGKC CRE was highly enriched in general water deficit-induced modules, such as in WD2, WD9, and WD11 modules. Similarly, CACGTG CRE was highly enriched in the WD2 and WD11 modules. Although the core *DRE* element (RCCGAC) was not enriched in the modules, the 7-mer *DRE*, RCCGACA, and other *DRE*-related CREs (e.g., RYCGAC and MACCGMCW) involved in drought responses were enriched in the WD9 and WD11 modules. Examples of genes containing *ABRE* and *G-box* CREs in promoters include all three water deficit up-regulated *VviNCEDs* (*VIT_05s0051g00670, VIT_10s0003g03750*, and *VIT_19s0093g00550*) – whose homologs in *Arabidopsis* (e.g., *AtNCED3*) regulate ABA biosynthesis during drought stress (Yamaguchi-Shinozaki and Shinozaki, 2006). Other CREs enrichment in these DE genes have been associated with drought-induced transcriptional pathways, including *NACR* (CATGTG enriched in the WD11 module and TTRCGT in the WD2 and WD11 modules), *GCC-box* (GCCGCC enriched in the WD1 and WD11 modules), and *MYB* (CCGTTA enriched in the WD2 and WD11 modules), while others may be novel, such as *AuxRE/ETT* (TGTCGG enriched in the WD2 and WD11 modules) and *ZAT6* (ACACTA enriched in the WD8 module).

Module-metabolite co-response was first determined for each module satisfying a PCC > 0.8, as a preliminary step to understand the coordinated regulation of modules with target metabolites (**Supplementary Table S6**). For instance, the WD2 module was correlated with 11 of 15 anthocyanin compounds profiled, in agreement with the many flavonoid pathway genes (e.g., four flavonoid 3′5′-hydroxylases –*VviF3*′*5*′*Hs*, the UDP-glucose:flavonoid-3-*O*-glucosyltransferase –*VviUFGT*, one leucoanthocyanidin dioxygenase –*VviLDOX*, and one flavanone 3-hydroxylases –*VviF3H*) that were localized in that module. To a lesser extent, the WD11 module was correlated with four different anthocyanin compounds coinciding with fewer flavonoid pathway genes (e.g., three *VviF3′5′Hs*, two chalcone synthases –*VviCHSs*, and one anthocyanin-acylglucoside transporter –*VviAnthoMATE2*). The WD9, WD2, and WD11 modules were correlated with various amino acids. For example, the WD11 module correlated to valine, threonine, and proline, and contains key structural genes involved in the formation of precursors for proline (such as *VviGluDH*, *VviGluS*, and *VviP5CS*) and threonine (e.g., threonine synthase – *VviTS*). Correlations between stilbenoid compounds with the modules considered were generally weak (PCC < 0.8), despite a large number of *VviSTS* genes being localized into WD3 (8 *VviSTSs*) and WD4 (19 *VviSTSs*) modules. As regards the correlation of modules with VOCs concentration, the WD2 module was correlated with 1-octen-3-ol and nonanol, and contained *VviHPL1*. Interestingly, several transcripts involved in the degradation of storage lipids such as triacylglycerol lipase and phospholipase A2 were also found in this module. Finally, strong correlations were observed between the WD5 module and zeaxanthin; consistently, the module included several structural carotenoid genes such as phytoene desaturase and ζ-carotene desaturase.

In order to discover new links between and within the many layers of biological complexity that governs the metabolic response of the grape berry to water deficit, the coordination between metabolite and gene patterns and water deficit responses was further investigated. For the metabolite-gene centered subnetwork, significant correlations between metabolites (anthocyanin and amino acids) and annotated structural pathway genes and TFs were investigated further for sub-modules DEV2, DEV3, and DEV5 in WD2 and WD11. The WD2 and WD11 modules were considered for anthocyanin (**Figure 8**), while only module WD11 was considered for amino acid metabolite-gene correlations (**Figure 8**). In addition, only regulatory genes from the bZIP, AP2/ERF, MYB, and NAC families were mainly considered given a consistent enrichment of the associated CREs in the promoters of both modules. Focusing on the anthocyanin subnetwork, we observe frequent correlations of bZIP (*VviABF4* and *VviGBF3*), AP2/ERF (*VviRAP2.1* and *VviRAP2.4*), and NAC (*VIT_02s0012g01040* and *VIT_10s0003g00350*) TFs with various anthocyanin in the WD2 module. Consistently, frequent correlations of AP2/ERF (*VviERF62*) and NAC (*VviNAC87* and *VviRD26*) TFs were also observed with anthocyanin in the WD11 module. As expected, frequent correlations of *VviMYBA1* (WD11) to three *VviF3′5′Hs*, and to eight anthocyanins further corroborate its role in regulating various anthocyanin pathway genes during berry development (Walker et al., 2007; Rinaldo et al., 2015) and under water deficit (Castellarin et al., 2007b). Interestingly, the *ABRE*, *DRE/CRT* and *GCC*, and/or *NACR* were also found in the promoter of *VviMYBA1* and of various flavonoid genes in WD11 module (**Supplementary Table S6**); this might provide additional clues into the regulation of anthocyanin biosynthesis under water deficit. In the amino acid subnetwork, *VviERF1* and *VviNAC1/VviNAC33* (*VIT_19s0027g00230*) were among the top five highly correlated TFs with proline in this module, and were also connected with *VviP5CS* in the network. *ERF1* is involved in the regulation of *P5CS* during normal growth and abiotic stresses in *Arabidopsis* (Cheng et al., 2013). Reinforcing the observed gene and metabolite correlations, *VviP5CS* promoter also contains CRE signatures potentially relevant for *VviERF1* binding, via the *GCC-box* (GCCGCC) and *DRE* (MACCGMCW) CRE (**Supplementary Table S6**) suggesting a conserved regulatory mechanism regulating proline via *VviP5CS* in the berries in response to water deficit stress. Other TF binding sites such as *NAC* (TTRCGT and TTACGTGT) (**Supplementary Table S6**) in *P5CS* promoter highlight its potential regulation via *VviNAC1/VviNAC33*. In addition, *VviERF62* and *VviRD26* were also correlated with *VviGluS* and CRE signatures related to *DRE*, *GCC-box*, and *NACR* are found within *VviGluS* promoter indicating potential regulation of these TFs on *VviGluS*.

## Discussion

Our results represent the first multi-omics study of the metabolic changes induced by water deficit in grape berry and, to the best of our knowledge, in any fruit. We revealed that both the ABA-dependent and ABA-independent signal transduction pathways are modulated by water deficit during fruit ripening, highlighting their central role in a reproductive organ such as the berry, in addition to their widely accepted roles in vegetative tissues of many plants (Yamaguchi-Shinozaki and Shinozaki, 2006; Shinozaki and Yamaguchi-Shinozaki, 2007). Gene-gene, gene-metabolite network analyses, as well as gene promoter analysis shed light on the poorly understood systems relationships between regulators, structural genes, and metabolites in the fruit response to water deficit not only in grapes but also in other fruits. The integrated network analysis associated genes involved in amino acid, phenylpropanoid, and flavonoid pathways with the stress-responsive TFs (bZIPs, AP2/ERFs, MYBs, and NACs) that took part in the water deficit-stress signal into tightly interconnected modules. Enrichment of specific CREs (ABRE, DRE, NACR, GCC-box, MYB, AuxRE/ETT, and ZAT6) in DE genes of specific modules was consistent with the module membership of TFs that recognize these sites. This provides strength into the role of these TFs in modulating water deficit-responsive genes. The analysis of gene-metabolite co-response networks in ripening-associated sub-modules led us to propose several *VviAP2/ERF* and *VviNAC* members as putative regulators of the amino acid and anthocyanin accumulation in the grape berries under water deficit. Network and promoter analysis also revealed that the participation of *VviARFs* and AuxRE, involved in the auxin signaling pathway, may be an important component of the fruit response to water deficit.

The coordinated response of genes and metabolites (**Figure 8** and **Supplementary Table S6**) to water deficit in a fleshy fruit is similar to what observed in various tissues of *A. thaliana* (Harb et al., 2010), *M. truncatula* (Zhang et al., 2014), and *Z. mays* (Opitz et al., 2016). Several studies have shown that both ABA-dependent and ABA-independent pathways are indispensable for water deficit response in vegetative tissues (Yamaguchi-Shinozaki and Shinozaki, 2006; Shinozaki and Yamaguchi-Shinozaki, 2007). Here, we show that similar mechanisms apply in a reproductive organ such as the grape berry. In regards to the ABA-dependent signaling pathway, several *VviAREB/ABFs* and *VvibZIP* genes were modulated by water deficit during berry ripening when deficit reached its highest severity. In regards to the ABA-independent pathway, *VviAP2/ERFs-DREBs* TFs were highly induced by water deficit (Mizoi et al., 2012) as reported in *Arabidopsis* (Lin et al., 2008; Cheng et al., 2013). The relevance for the observed modulation of these pathways in the grape may be attributed with higher ABA sensitivity, reduced transpiration rate, and improved drought tolerance (Kang et al., 2002; Fujita et al., 2005).

In our dataset, one TF in particular (*VviHB12*) was up-regulated by water deficit at 53, 67, 81, and 106 DAA. The induction of *VviHB12* at 53 DAA, just before the onset of ripening and when major effects of water deficit on the level of transcripts and metabolites were not observed yet, suggests that *VviHB12* may be involved in one of the earliest responses to water deficit. The *Arabidopsis AtHB12* is strongly induced by water deficit and ABA, and participates in the regulation of ABA signaling through the regulation of *PP2C* and ABA receptor gene expression (Olsson et al., 2004; Valdés et al., 2012). Similarly to what was found in *Arabidopsis*, the overexpression of this gene is also related with the higher expression of a *VviPP2C* highly expressed during fruit ripening (Supplementary Figure S3).

The involvement of multiple stress regulons (Nakashima et al., 2014) might be critical to orchestrate the balance between stress-responsive regulation and the berry ripening program. These examples suggest that increased osmotic stress signals induced by water deficit may further fine-tune the ripening program through regulating multiple interacting TFs, possibly accelerating ripening (Castellarin et al., 2007b; Herrera and Castellarin, 2016). Further supporting this, several TF ‘switch’ genes that may be master regulators of berry ripening, such as *VviMYBA1-2*, *VviNAC1/VviNAC33-47-71*, and *VviLBD15-18-38* (Palumbo et al., 2014), are induced under water deficit from the onset of ripening onward.

Furthermore, this study confirmed several metabolic reprogramming patterns previously reported in grapevine as well as in other plants. WD increased the accumulation of proline and branch chain amino acids such as leucine, valine, and isoleucine, confirming their role in drought response in grapevine (Deluc et al., 2009; Hochberg et al., 2015), *A. thaliana* (Nambara et al., 1998; Urano et al., 2009) and *O. sativa* (Maruyama et al., 2014). In the case of proline increase a parallel up-regulation of the key biosynthetic gene *VviP5CS* and of *VviGluDH* was observed. A study in tobacco and grape has shown that abiotic stress-induced ROS activate GluDH expression and enhances GluDH activity to produce glutamate for proline biosynthesis (Skopelitis et al., 2006). The coordinative induction of *VviGluDH* with *VviP5CS* under water deficit, observed in WD berries during ripening, may be a conserved mechanism necessary for maintaining a large amount of glutamate available for proline accumulation. NAC binding sites in *VviP5CS* promoter and the CRE signatures related to *DRE*, *GCC-box*, and *NACR* found within *VviGluS* promoter indicate potential regulation of these TFs on the proline accumulation. Direct implication of *NAC1* on *P5CS*, or *RD26* and *ERF62* on *GluS* regulation has not been shown in plants, but some evidences show that other plant NAC and AP2/ERF members, such as *JUNGBRUNNEN1/ANAC042* (Wu et al., 2012), *OsNAC5* (Song et al., 2011), or *GmERF3* (Zhang et al., 2009) are directly implicated in drought stress-mediated proline accumulation.

Several transcripts involved in phenylpropanoid and flavonoid biosynthesis, including *VviMYB5b –* a generic regulator of this pathway (Deluc et al., 2008; Cavallini et al., 2014), were enhanced by water deficit in parallel with a higher accumulation of related metabolites, such as benzoic and cinnamic acids, and anthocyanins. Previous studies have already reported a modulation of these pathways in grape berries exposed to water deficit (Deluc et al., 2009; Savoi et al., 2016). Nonetheless, our gene-metabolite network analysis identified correlations between specific structural and regulatory flavonoid genes (e.g., *CHSs*, *LDOX*, *UFGT*, *AOMT*, *F3′5′Hs*, and *MybAs*) and anthocyanin modulated by water deficit.

The large demand for precursor for anthocyanins production possibly determines the observed impairment of stilbenoid production which decreased both in biosynthesis and concentration, indicating a redirection of phenylpropanoids to the flavonoid pathway instead to the stilbenoid one. However, the two MYBs, *VviMYB14* and *VviMYB15*, that regulate stilbene biosynthesis in grapevine (Höll et al., 2013) do not correlate with the transcripts levels of *VviSTSs* modulated in WD berries, suggesting that other TFs might contribute to the stilbenoid regulation (Wong et al., 2016). One candidate could be *VviMYBC2-L1*, a negative regulator of flavonoid (anthocyanin and proanthocyanidin) and stilbenoid biosynthesis (Huang et al., 2014; Cavallini et al., 2015), that was up-regulated at 67 and 81 DAA, potentially repressing *VviSTS* transcripts. Stilbenoid production increased in Cabernet Sauvignon berries exposed to water deficit (Deluc et al., 2011) but were not significantly affected in Tocai Friulano (Savoi et al., 2016), indicating that the degree of water deficit and the genotype may be key factors for stilbenoid accumulation under drought events.

Analysis of gene-metabolite co-response networks in ripening-associated sub-modules, revealed a strong coordinated response between structural pathway genes and metabolite whilst identifying known regulators for grape anthocyanin biosynthesis (e.g., *VviMYBA1-2*). Gene-metabolite correlation networks has been successfully applied to prioritize candidate genes involved the control of fruit composition and development in tomato (Mounet et al., 2009) and in grapes (Zamboni et al., 2010; Savoi et al., 2016). In this study, we identified new candidate regulators for anthocyanin compounds, including several *VviNACs* that may play direct and/or indirect roles in regulating structural genes or specific pathway regulators, respectively. A recent study demonstrated that NAC TFs (*PpBL*, *PpNAC1*, and *PpNAC2*) can *trans*-activate *PpMYB10.1* (homologs to *VviMYBA1-2*) promoter and that the silencing of *PpBL* inhibits anthocyanin pigmentation in peach fruits (Zhou et al., 2015). Little is known on the regulation of grape amino acid metabolism. The WD11 metabolite-gene subnetwork centered on proline, valine, and threonine correlated with the expected pathway genes such as *VviP5CS*, *VviGluDH*, *VviGluS*, *VviTS*, and other amino acid metabolism genes, and with TFs, such as *VviERF1, VviNAC1, and VviERF62.* Functional validation of these regulatory modules is necessary to confirm their role in the regulation of critical anthocyanin and amino acid biosynthetic genes in response to water deficit.

Many *ABRE*, *DRE/CRT*, and *NACR* CREs involved in stress-responsive transcription in vegetative tissues (Yamaguchi-Shinozaki and Shinozaki, 2006) were found in promoters of DE genes and were also enriched in water deficit-induced modules. This highlights the conserved role of these stress-responsive CREs in modulating water deficit-responsive genes in reproductive tissues such as berries. Nonetheless, our analysis also show additional CRE pertaining to auxin responses may be an important component of the fruit response to water deficit, an observation/role that has not been implicated before in other fruit systems. Enrichment of the auxin response element (*AuxRE/ETT* – TGTCGG) in water deficit-induced modules (e.g., WD2 and WD11) suggests that ARFs that bind to these sites may play an important role in regulating water deficit-induced genes in berries, potentially via auxin signaling. ARFs have relevant function in drought-stress responses in plants like *Glycine max* (Ha et al., 2013) and regulate many aspects of fruit development and ripening (Kumar et al., 2014). Some of them [e.g., *SlARF2*, Hao et al. (2015)] are central components of fruit (tomato) development and ripening regulatory network. These observations reinforces that stress-responsive CREs may serve a critical role in ripening regulatory networks and fruit maturation. Many fruit ripening-associated TFs bind to these elements (Karlova et al., 2014; Kumar et al., 2014; Leng et al., 2014), of which some (e.g., *VviABF2* and tomato *SlNAC4* – homologs of *Arabidopsis ATAF1* and *VviATAF1*) are known to regulate both abiotic stress responses and fruit ripening (Nicolas et al., 2014; Zhu et al., 2014). Finally, limited water availability affects VOCs production in several plant organs (Nowak et al., 2010; Griesser et al., 2015), including fleshy fruits, such as apple (Behboudian et al., 1998; Hooijdonk et al., 2007), and tomato (Veit-Köhler et al., 1999). We have recently reported that water deficit modulates the synthesis of monoterpenes in white grapes (Savoi et al., 2016) and, interestingly, at the end of ripening several volatiles such as 1-octen-3-one, (E)-2-heptenal, (E)-2-octenal, and nonanol were commonly up-regulated by water deficit in Merlot as well as in Tocai Friulano. The higher accumulation of VOCs compounds under water deficit may be the result of complex modulation of fatty acid degradation pathway genes, such as *VviLOX* and *VviHPL1* observed here (**Supplementary Table S5**). Furthermore, consistent up-regulation of triacylglycerol lipase and phospholipase A2 transcripts suggests an additional role for storage lipid degradation on various VOC accumulation. In cucumber, phospholipase A2 has been demonstrated increase the susceptibility of lipid body membrane to lipolytic enzymes such as LOX and lipases via partial degradation of membrane proteins and associated phospholipid monolayer (Rudolph et al., 2011).

Indeed, silencing of *TomloxC* in tomatoes has been shown to increase C8 and C10 compounds (Orzaez et al., 2009) and decrease C5 and C6 ones in ripe fruits. This is probably due to higher precursor availability to other LOXs that may catalyze the production of C8 and C9 precursors (Senger et al., 2005). Shen et al. (2014) also demonstrated that the modulation of tomato *HPL* correlates with changes in C5 and C6 volatiles in fruits. Consistent induction of *VviHPL1* from the onset of ripening onward may explain higher accumulation of VOC aldehydes under water deficit. Moreover, a role of LOXs and HPLs in the drought adaptation process has been already suggested in *Arabidopsis* (Grebner et al., 2013; Savchenko et al., 2014). While various chain length volatiles impart important flavor attributes (Baldwin et al., 2004; Ripoll et al., 2014), these compounds may also act as powerful signaling molecules activating abiotic stress response (Alméras et al., 2003; Yamauchi et al., 2015) and potentially hasten the developmental program in berries during water deficit. Both abiotic stress and ripening processes involves oxidative stress signals. Recent studies have demonstrated that *(E)-*2-hexenal, 2-butenal, and 3-hepten-2-one treatment in *Arabidopsis* seedlings can trigger large transcriptome changes involving abiotic stress genes and TFs, such as *DREBs* (Yamauchi et al., 2015). The possibility of VOCs in modulating maturation-related transcriptome changes (e.g., senescence and ripening) in fruit tissues should not be discounted in light of these observations.

## Conclusion

Our results confirm several previously reported modulation of the primary and specialized metabolism and also provide new insight into the stilbenoid and volatile compounds response to water deficit. The integration with network analysis revealed major water deficit-regulated gene modules that are strongly linked to central and specialized metabolites as well as multiple signal transduction pathways (e.g., regulation of anthocyanin and amino acids via members of *VviAP2/ERF* and *VviNAC* TF families). Activation of both ABA-dependent and ABA-independent signaling pathway may also be critical to achieve a balance between the regulation of the stress response and the berry ripening program. Further functional analyses are needed to characterize the putative identified modulators of this metabolic response. This study represents a first step into understanding the transcriptional control and their downstream regulatory cascades in grapes or other fruits while providing an important resource for breeding opportunities, irrigation management, and comparative studies centered on reproductive tissue metabolism under abiotic stress in fruit crops.

## Author Contributions

SS participated in the design of the study, carried out the specialized metabolite analyses, RNA extractions, part of the transcriptome data analysis, and drafted part of the manuscript; DCJW carried out part of the transcriptome data analysis, the network analysis, and drafted part of the manuscript; AD carried out the central metabolite analysis; JCH carried out the anthocyanin analysis; BB performed the field experiment; EP coordinated the field experiments; AF supervised the central metabolite analysis and critically revised the manuscript; FM participated in the design of the study, supervised the metabolite analysis, and critically revised the manuscript; SDC conceived the study, coordinated the experiments, supervised the field experiment, transcriptome analysis, and network analysis, interpreted the results, and drafted part of the manuscript. All authors read and approved the final manuscript.

## Conflict of Interest Statement

The authors declare that the research was conducted in the absence of any commercial or financial relationships that could be construed as a potential conflict of interest.

## Footnotes

1 http://www.ncbi.nlm.nih.gov/sra

## Supplementary Material

**TABLE S1 |** List of genes and primer sequences assayed for expression by qPCR.

**TABLE S2 |** Impact of water deficit on grape productivity.

**TABLE S3 |** List of compounds identified in this study using GC-MS, UHPLC-MS/MS, HPLC-DAD, and HS-SPME-GC-MS platforms.

**TABLE S4 |** RNA sequencing analysis metrics.

**TABLE S5 |** Summary of differentially expressed genes and associated information at 26 **(A)**, 53 **(B)**, 67 **(C)**, 81 **(D)**, and 106 **(E)** days after anthesis. **(F)** TFs differentially expressed. **(G)** Information on TFs reported in **Figure 7**. **(H)** List of differentially expressed genes involved in ROS production, scavenging, and signaling pathways and associated information.

**TABLE S6 |(A)** Weighted correlation gene co-expression network analysis (WGCNA). **(B)** CRE sequence, length, and description, number of genes containing each CRE, total occurrence in the genome, enrichment value, and the list of genes containing each CRE. **(C)** Module-metabolite co-response matrix determined for each module.
