# Supplementary material for: Multi-Omics and Integrated Network Analyses Reveal New Insights into the Systems Relationships between Metabolites, Structural Genes, and Transcriptional Regulators in Developing Grape Berries (Vitis vinifera L.) Exposed to Water Deficit
Source: Front Plant Sci. 2017 Jul 10;8:1124. doi: 10.3389/fpls.2017.01124 (PMC5502274; doi:10.3389/fpls.2017.01124)
Supplement: Supplementary file 1 [file Table_1.PDF]

Supplementary Table S1. List of genes assayed for expression by qPCR. For each gene, the efficiency and amplificatory factors, forward and reverse primers, and literature references are shown.

Gene ID	Efficiency	Amplification Factor	Forward / Reverse primer sequence	Reference
VviAP47 (HOUSEKEEPING) *(VIT_02s0012g00910)*	109 %	2.09	GGTTCCCATGTTTACAGCATCTG / GCACCCACTCAACGGTATTGTAC	Newly designed
VviP5CS *(VIT_13s0019g02360)*	95 %	1.95	TTGCAGCTAAAGGCTGACCT / TCCAACCCTCGACTTGTCTC	Newly designed
VviGluDH *(VIT_16s0039g02750)*	113 %	2.13	ATGCTGGAGGTGTGACTGTG / ACTGCAGTTGTGCGATTGAC	Newly designed
VviArgDC *(VIT_03s0038g00760)*	105 %	2.05	GAGATGTGCTCCGAGTGATG / GAAGAACCCGCCACAAGATA	Newly designed
VviCHS3 *(VIT_05s0136g00260)*	---	---	GTTTCGGACCAGGGCTCACT / GGCAAGTAAAGTGGAAACAG	Goto-Yamamoto 2002
VviSTS 25+27+29 *(VIT_16s0100g00770)*	133 %	2.33	GGTTTTGGACCAGGCTTGACT / GAGATAAATACCTTACTCCTATTCAAC	Höll 2013
VviUFGT *(VIT_16s0039g02230)*	111 %	2.11	GGGGTTTTCACAGAGAGTGG / AAATCCACCAGGGTTTTGAA	Newly designed
VviAnthoMate2 *(VIT_16s0050g00910)*	115 %	2.15	AACAGTATGCAGCCAGTGATTT / CACCCCCAAATTTGCTTTGT	Newly designed
VviHPL1 *(VIT_12s0059g01060)*	108 %	2.08	GACCCGGAAACTTTCGTACC / CAGCGGTCATGGTGACATAG	Newly designed
VviBZO2H3 *(VIT_14s0030g02200)*	107 %	2.07	ACCCATGACCCAAGAATCAA / CTTTTGCAGATGCTCCAAGC	Newly designed
VviRD26 *(VIT_19s0014g03290)*	104 %	2.04	TCCAAGCCGAAGAAGAAGTC / CCACTAGGTTGGGTCGGGTA	Newly designed
VviNAC29 *(VIT_01s0026g02710)*	108 %	2.08	TTTGATTTCCAAGGCACCAT / TCACAAATAGAGGCTGGTTCG	Newly designed
VviPP2C *(VIT_06s0004g05460)*	117 %	2.17	CCCAGAGGACGAGTGTTTGA / GCACCTATCCCAGCTCCAGT	Newly designed
