# Supplementary material for: Multi-Omics and Integrated Network Analyses Reveal New Insights into the Systems Relationships between Metabolites, Structural Genes, and Transcriptional Regulators in Developing Grape Berries (Vitis vinifera L.) Exposed to Water Deficit
Source: Front Plant Sci. 2017 Jul 10;8:1124. doi: 10.3389/fpls.2017.01124 (PMC5502274; doi:10.3389/fpls.2017.01124)
Supplement: Supplementary file 2 [file Table_2.PDF]

Supplementary Table S2. Impact of water deficit on crop productivity. Cluster per vine, cluster weight and yield per plant of CT and WD grapes in 2011 and 2012. Values are averages ± the standard error. Differences between treatments (P<0.05) were assessed with a one-way ANOVA. The level of significance is reported within the columns: * or ns, P < 0.05 or not significant, respectively.

	**Merlot**			
**Year**	2011		2012	
**Treatment**	CT	WD	CT	WD
**Cluster per Vine**	22.9 ± 0.6	22.8 ± 1.2	20.1 ± 1.7	19.4 ± 1.4
	ns		ns	
**Cluster Weight (g)**	189.4 ± 11.5	142.4 ± 5.9	129.0 ± 4.2	101.5 ± 9.3
	*		*	
**Yield per Plant (Kg)**	4.29 ± 0.35	3.23 ± 0.22	2.62 ± 0.22	1.97 ± 0.19
	*		*	
