# Supplementary material for: Multi-Omics and Integrated Network Analyses Reveal New Insights into the Systems Relationships between Metabolites, Structural Genes, and Transcriptional Regulators in Developing Grape Berries (Vitis vinifera L.) Exposed to Water Deficit
Source: Front Plant Sci. 2017 Jul 10;8:1124. doi: 10.3389/fpls.2017.01124 (PMC5502274; doi:10.3389/fpls.2017.01124)
Supplement: Supplementary file 3 [file Table_3.PDF]

Supplementary Table S3. List of compounds identified in this study using GC-MS, UHPLC-MS/MS, HPLC-DAD, and HS-SPME-GC-MS platforms.

Compound	Chemspider ID	Chemical Class	Analytical Platform
Leucine	5880	Amino acids	GC-MS
Valine	6050	Amino acids	GC-MS
Isoleucine	6067	Amino acids	GC-MS
Serine	597	Amino acids	GC-MS
Ethanolamine	13835336	Amino acids	GC-MS
Alanine	5735	Amino acids	GC-MS
Threonine	6051	Amino acids	GC-MS
Proline	128566	Amino acids	GC-MS
Putrescine	13837702	Polyamine	GC-MS
Glycerate	732	Organic Acids	GC-MS
Lactate	592	Organic Acids	GC-MS
Glycolate	737	Organic Acids	GC-MS
Quinate	10246715	Organic Acids	GC-MS
Shikimate	8412	Organic Acids	GC-MS
Citrate	305	Organic Acids	GC-MS
Succinate	1078	Organic Acids	GC-MS
Fumarate	10197150	Organic Acids	GC-MS
Maleate	392248	Organic Acids	GC-MS
Malate	510	Organic Acids	GC-MS
Threonate	133224	Organic Acids	GC-MS
Tartrate	852	Organic Acids	GC-MS
Lactone Threonate	2006913	Organic Acids	GC-MS
Sucrose	5768	Sugars	GC-MS
Glucose	96749	Sugars	GC-MS
Fructose	5764	Sugars	GC-MS
Glucose-6-phosphate	388417	Sugars	GC-MS
Fructose-6-phosphate	62713	Sugars	GC-MS
Myo-Inositol	10239179	Polyols	GC-MS
Raffinose	388379	Polyols	GC-MS
Galactinol	19402461	Polyols	GC-MS
Arabitol	84971	Polyols	GC-MS
Erythritol	13835932	Polyols	GC-MS
Threitol	147828	Polyols	GC-MS
Glycerol	733	Polyols	GC-MS
Gallic acid	361	Benzoic acids	UHPLC-MS/MS
Ellagic Acid	4445149	Benzoic acids	UHPLC-MS/MS

trans-Caftaric acid	4944664	Cinnamic acids	UHPLC-MS/MS
trans-Coutaric acid	26325199	Cinnamic acids	UHPLC-MS/MS
trans-Fertaric acid	20058463	Cinnamic acids	UHPLC-MS/MS
trans-Resveratrol	392875	Stilbenes	UHPLC-MS/MS
cis-Piceid	8353968	Stilbenes	UHPLC-MS/MS
trans-Piceid	4445034	Stilbenes	UHPLC-MS/MS
Piceatannol	581006	Stilbenes	UHPLC-MS/MS
Astringin	4445028	Stilbenes	UHPLC-MS/MS
Pallidol	425065	Stilbenes	UHPLC-MS/MS
Isorhapontin	4445032	Stilbenes	UHPLC-MS/MS
Phlorizin	16498836	Dihydrochalcones	UHPLC-MS/MS
(+)-Catechin	8711	Flavan-3-ols	UHPLC-MS/MS
(-)-Epicatechin	65230	Flavan-3-ols	UHPLC-MS/MS
(-)-Epicatechin gallate	97034	Flavan-3-ols	UHPLC-MS/MS
(+)-Gallocatechin	58594	Flavan-3-ols	UHPLC-MS/MS
(-)-Epigallocatechin	65231	Flavan-3-ols	UHPLC-MS/MS
(-)-Epigallocatechin gallate	58575	Flavan-3-ols	UHPLC-MS/MS
Procyanidin B1	9425166	Proanthocyanidins	UHPLC-MS/MS
Procyanidin B2+B4	109417, 129882	Proanthocyanidins	UHPLC-MS/MS
Procyanidin B3	129476	Proanthocyanidins	UHPLC-MS/MS
Quercetin-3-*O*-rutinoside	4444362	Flavonols	UHPLC-MS/MS
Isorhamnetin-3-*O*-rutinoside	4587469	Flavonols	UHPLC-MS/MS
Cyanidin-3-*O*-glucoside	390284	Anthocyanins	HPLC-DAD
Peonidin-3-*O*-glucoside	391786	Anthocyanins	HPLC-DAD
Delphinidin-3-*O*-glucoside	145096	Anthocyanins	HPLC-DAD
Petunidin-3-*O*-glucoside	153683	Anthocyanins	HPLC-DAD
Malvidin-3-*O*-glucoside	391785	Anthocyanins	HPLC-DAD
Cyanidin-3-*O*-(6"-acetyl)-glucoside		Anthocyanins	HPLC-DAD
Peonidin-3-*O*-(6"-acetyl)-glucoside		Anthocyanins	HPLC-DAD
Delphinidin-3-*O*-(6"-acetyl)-glucoside		Anthocyanins	HPLC-DAD
Petunidin-3-*O*-(6"-acetyl)-glucoside		Anthocyanins	HPLC-DAD
Malvidin-3-*O*-(6"-acetyl)-glucoside		Anthocyanins	HPLC-DAD
Cyanidin-3-*O*-(6"-p-coumaroyl)-glucoside		Anthocyanins	HPLC-DAD
Peonindin-3-*O*-(6"-p-coumaroyl)-glucoside		Anthocyanins	HPLC-DAD
Delphinidin-3-*O*-(6"-p-coumaroyl)-glucoside		Anthocyanins	HPLC-DAD
Petunidin-3-*O*-(6"-p-coumaroyl)-glucoside		Anthocyanins	HPLC-DAD
Malvidin-3-*O*-(6"-p-coumaroyl)-glucoside		Anthocyanins	HPLC-DAD
β-Carotene	4444129	Carotenoids	HPLC-DAD
Zeaxanthin	4444421	Carotenoids	HPLC-DAD
Antheraxanthin	4444635	Carotenoids	HPLC-DAD

Violaxanthin	395237	Carotenoids	HPLC-DAD
9-*(Z)*-Neoxanthin	4444659	Carotenoids	HPLC-DAD
Lutein	4444655	Carotenoids	HPLC-DAD
Lutein, 5-6 epoxide	4444656	Carotenoids	HPLC-DAD
Hexanal	5949	C6 Compounds	HS-SPME-GC-MS
(E)-2-Hexenal	4444608	C6 Compounds	HS-SPME-GC-MS
Hexanol	7812	C6 Compounds	HS-SPME-GC-MS
3-Hexenol	4447565	C6 Compounds	HS-SPME-GC-MS
(E)-2-Pentenal	4516892	C5 Compounds	HS-SPME-GC-MS
1-Penten-3-ol	11525	C5 Compounds	HS-SPME-GC-MS
Heptanal	7838	C7 Compounds	HS-SPME-GC-MS
(E)-2-Heptenal	4446437	C7 Compounds	HS-SPME-GC-MS
Heptanol	7837	C7 Compounds	HS-SPME-GC-MS
Octanol	932	C8 Compounds	HS-SPME-GC-MS
(E)-2-Octenal	4446445	C8 Compounds	HS-SPME-GC-MS
1-Octen-3-ol	17778	C8 Compounds	HS-SPME-GC-MS
1-Octen-3-one	55282	C8 Compounds	HS-SPME-GC-MS
Nonanal	29029	C9 Compounds	HS-SPME-GC-MS
Nonanol	8574	C9 Compounds	HS-SPME-GC-MS
Linalool	13849981	Terpenes	HS-SPME-GC-MS
Geraniol	558917	Terpenes	HS-SPME-GC-MS
Hotrienol	4518161	Terpenes	HS-SPME-GC-MS
α-Terpineol	13850142	Terpenes	HS-SPME-GC-MS
β-Ionone	553581	C13 Norisoprenoids	HS-SPME-GC-MS
β-Damascenone	4517997	C13 Norisoprenoids	HS-SPME-GC-MS
