# Supplementary material for: Multi-Omics and Integrated Network Analyses Reveal New Insights into the Systems Relationships between Metabolites, Structural Genes, and Transcriptional Regulators in Developing Grape Berries (Vitis vinifera L.) Exposed to Water Deficit
Source: Front Plant Sci. 2017 Jul 10;8:1124. doi: 10.3389/fpls.2017.01124 (PMC5502274; doi:10.3389/fpls.2017.01124)
Supplement: Supplementary file 4 [file Table_4.PDF]

**Supplementary Table S4.** RNA sequencing analysis metrics. Transcriptome analyses were performed in CT and WD berries in 2012 at five selected developmental stages (26 DAA and 53 DAA, before the onset of ripening; 67 DAA, at the onset of ripening; 81 and 106 DAA, during ripening) using an Illumina HiSeq platform.

| Sampling (DAA) | Treatment | Biol. Rep. | Sequenced Reads | Trimmed and Filtered Reads | Mapped Reads | Unique Reads | Expressed Genes |
|---|---|---|---|---|---|---|---|
| 26 | CT | 1 | 32,282,090 | 27,285,970 | 25,243,216 | 24,173,521 | 22,949 |
| 26 | CT | 2 | 23,218,489 | 22,903,285 | 20,935,341 | 20,020,535 | 23,576 |
| 26 | CT | 3 | 18,987,430 | 18,756,732 | 17,497,629 | 16,763,590 | 23,214 |
| 26 | WD | 1 | 38,030,560 | 32,117,022 | 29,449,144 | 28,148,361 | 22,829 |
| 26 | WD | 2 | 21,823,539 | 21,554,999 | 20,080,109 | 19,207,041 | 23,298 |
| 26 | WD | 3 | 26,975,517 | 26,651,368 | 24,776,353 | 23,663,710 | 23,654 |
| 53 | CT | 1 | 48,502,153 | 41,329,184 | 36,962,611 | 35,181,638 | 23,334 |
| 53 | CT | 2 | 32,683,299 | 31,833,552 | 26,901,141 | 25,540,691 | 23,021 |
| 53 | CT | 3 | 33,287,117 | 32,513,137 | 28,120,305 | 26,644,501 | 23,155 |
| 53 | WD | 1 | 35,861,125 | 34,996,173 | 30,361,145 | 28,905,599 | 23,243 |
| 53 | WD | 2 | 32,371,631 | 31,790,674 | 28,156,175 | 26,746,733 | 23,156 |
| 53 | WD | 3 | 31,895,415 | 31,177,159 | 27,397,806 | 26,058,126 | 23,411 |
| 67 | CT | 1 | 29,986,521 | 29,562,710 | 27,225,348 | 25,908,529 | 22,098 |
| 67 | CT | 2 | 29,979,931 | 29,554,286 | 27,175,999 | 25,813,402 | 22,140 |
| 67 | CT | 3 | 26,456,360 | 26,061,866 | 24,057,180 | 22,879,874 | 22,180 |
| 67 | WD | 1 | 28,029,734 | 27,594,638 | 25,227,554 | 23,570,934 | 21,530 |
| 67 | WD | 2 | 31,262,847 | 30,798,680 | 28,119,560 | 26,615,329 | 22,069 |
| 67 | WD | 3 | 30,934,072 | 30,494,505 | 28,192,068 | 26,644,349 | 21,963 |
| 81 | CT | 1 | 56,213,525 | 44,149,321 | 41,653,412 | 39,672,979 | 22,813 |
| 81 | CT | 2 | 41,388,889 | 40,728,237 | 37,824,937 | 36,009,744 | 22,715 |
| 81 | CT | 3 | 20,751,950 | 20,405,881 | 18,550,887 | 17,634,540 | 21,760 |
| 81 | WD | 1 | 41,321,398 | 40,656,157 | 37,710,401 | 35,847,981 | 22,603 |
| 81 | WD | 2 | 37,894,910 | 37,305,935 | 34,633,903 | 32,943,905 | 22,615 |
| 81 | WD | 3 | 28,343,487 | 27,893,814 | 25,611,214 | 24,362,357 | 22,209 |
| 106 | CT | 1 | 24,799,483 | 24,431,124 | 22,611,345 | 21,613,028 | 21,964 |
| 106 | CT | 2 | 44,340,639 | 43,707,436 | 40,246,983 | 38,392,957 | 22,752 |
| 106 | CT | 3 | 33,374,833 | 29,502,167 | 27,184,432 | 25,977,126 | 21,066 |

| | | | | | | | |
|---|---|---|---|---|---|---|---|
| 106 | WD | 1 | 29,059,685 | 28,620,355 | 26,418,924 | 25,190,721 | 21,961 |
| 106 | WD | 2 | 32,545,793 | 32,087,904 | 29,760,625 | 28,365,623 | 22,352 |
| 106 | WD | 3 | 40,625,594 | 40,038,315 | 36,845,815 | 35,126,958 | 22,878 |
