# Supplementary material for: Multi-Omics and Integrated Network Analyses Reveal New Insights into the Systems Relationships between Metabolites, Structural Genes, and Transcriptional Regulators in Developing Grape Berries (Vitis vinifera L.) Exposed to Water Deficit
Source: Front Plant Sci. 2017 Jul 10;8:1124. doi: 10.3389/fpls.2017.01124 (PMC5502274; doi:10.3389/fpls.2017.01124)
Supplement: Supplementary file 7 [file Image_1.PDF]

Amino acids and Polyamine

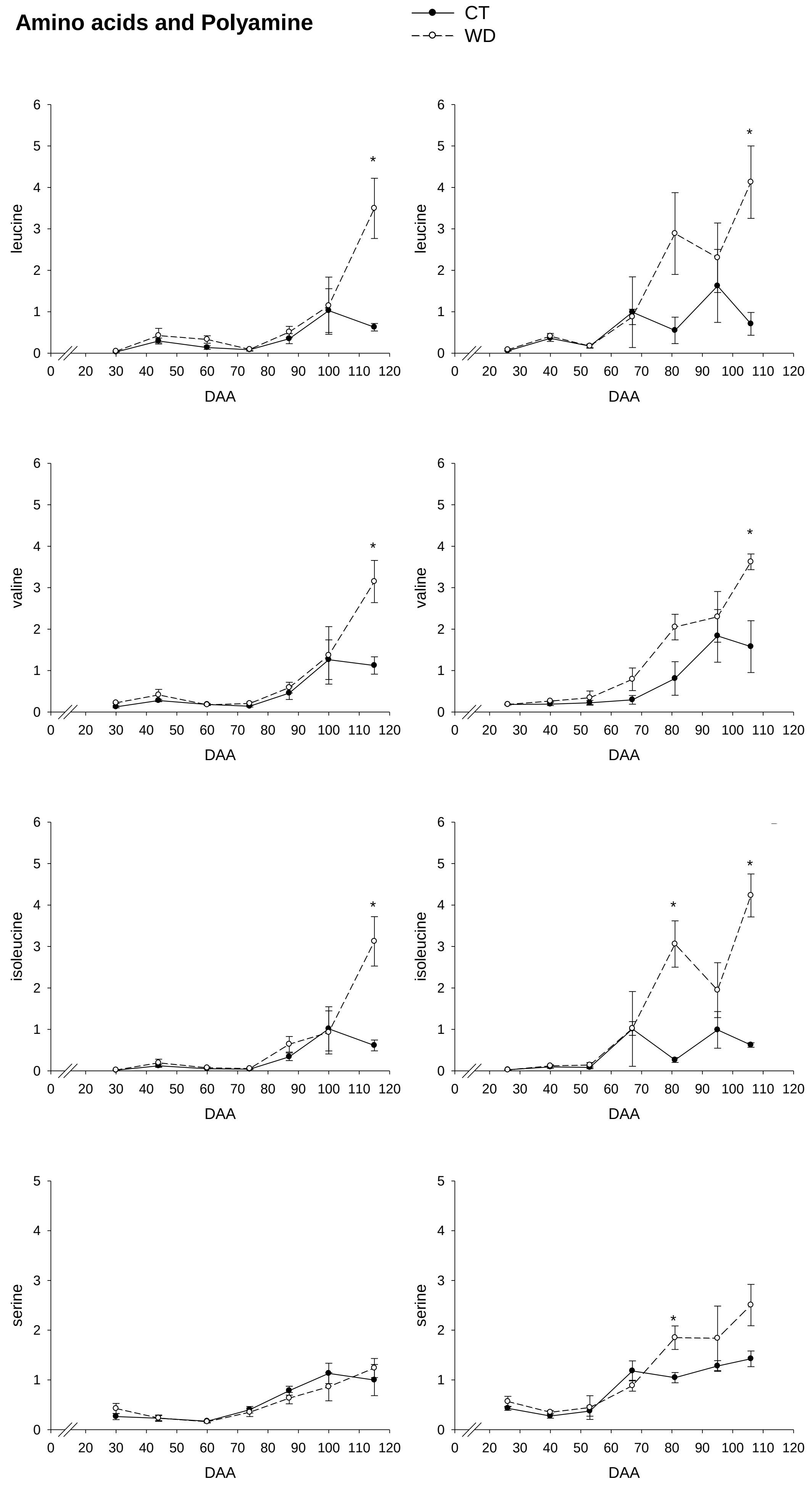

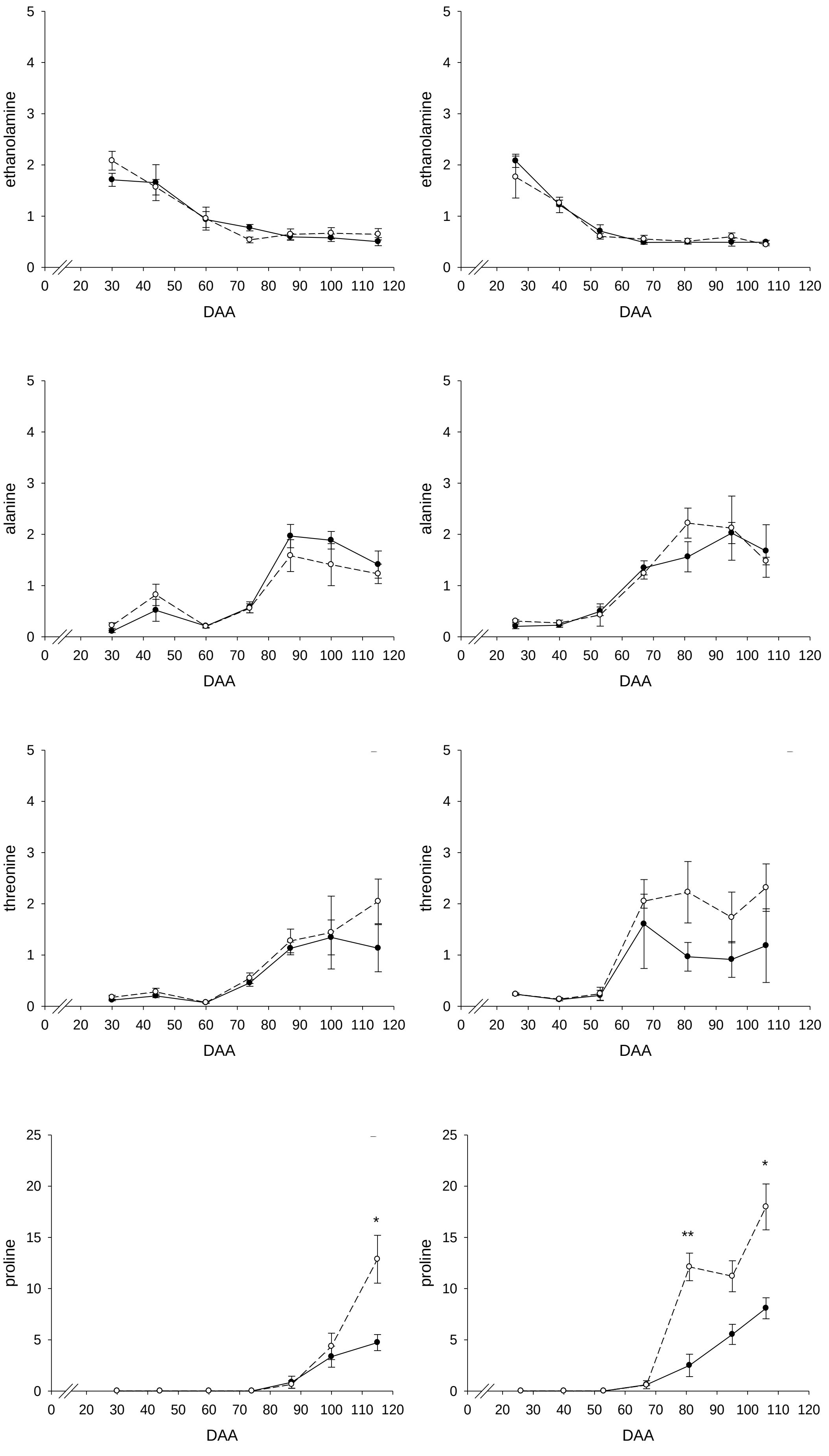

ethanolamine
DAA
ethanolamine
DAA
alanine
DAA
alanine
DAA
threonine
DAA
threonine
DAA
proline
*
DAA
proline
**
*
DAA

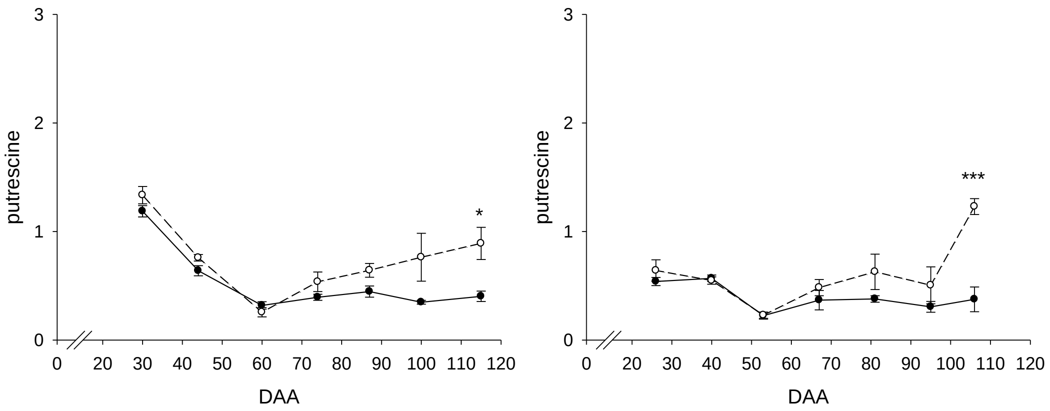

putrescine
DAA
*
putrescine
DAA
***

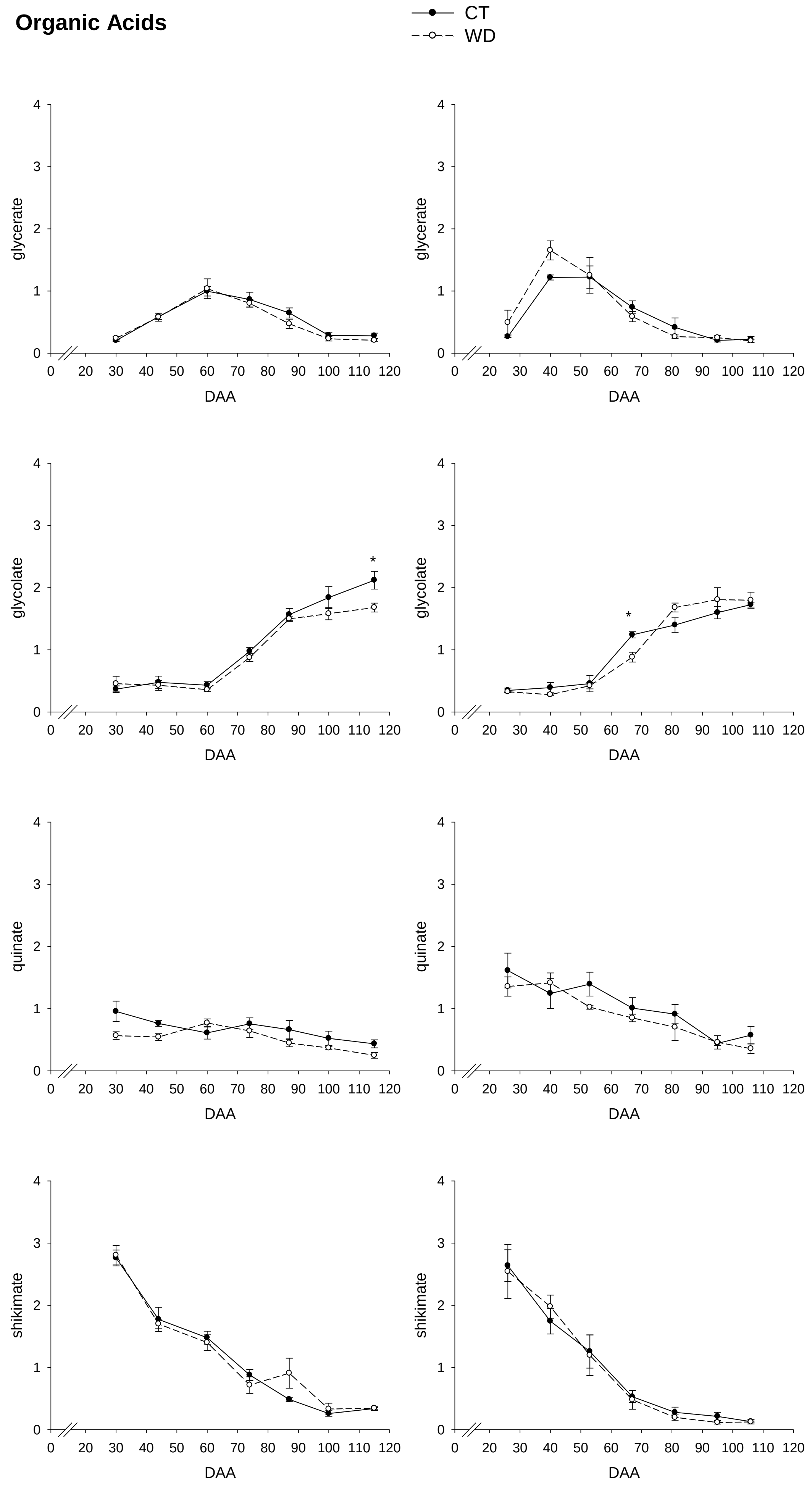
Organic Acids
CT
WD
glycerate
glycolate
quinate
shikimate
DAA
0
1
2
3
4
0 20 30 40 50 60 70 80 90 100 110 120
*

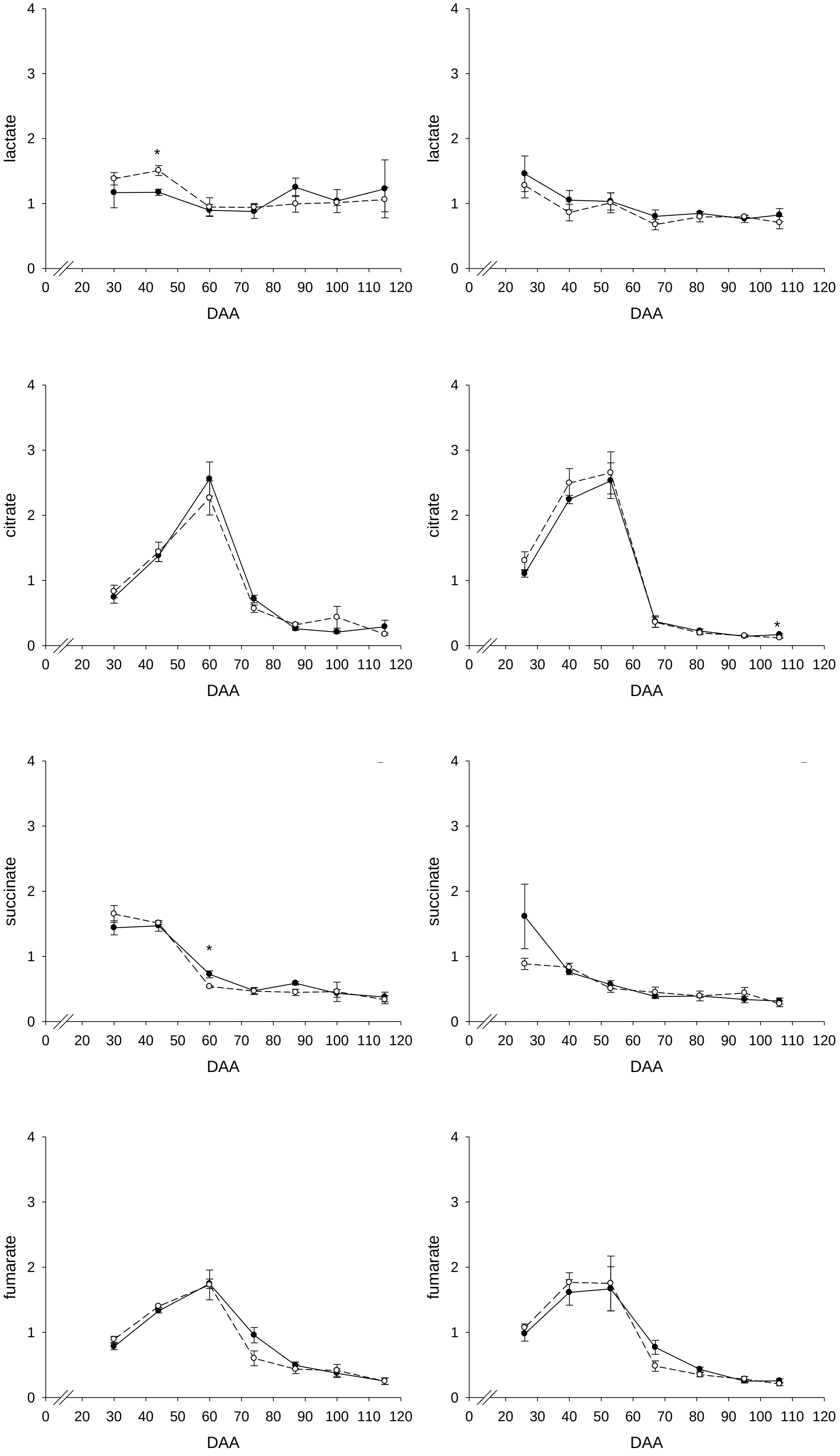

lactate
citrate
succinate
fumarate
DAA
0
1
2
3
4
0 20 30 40 50 60 70 80 90 100 110 120
*

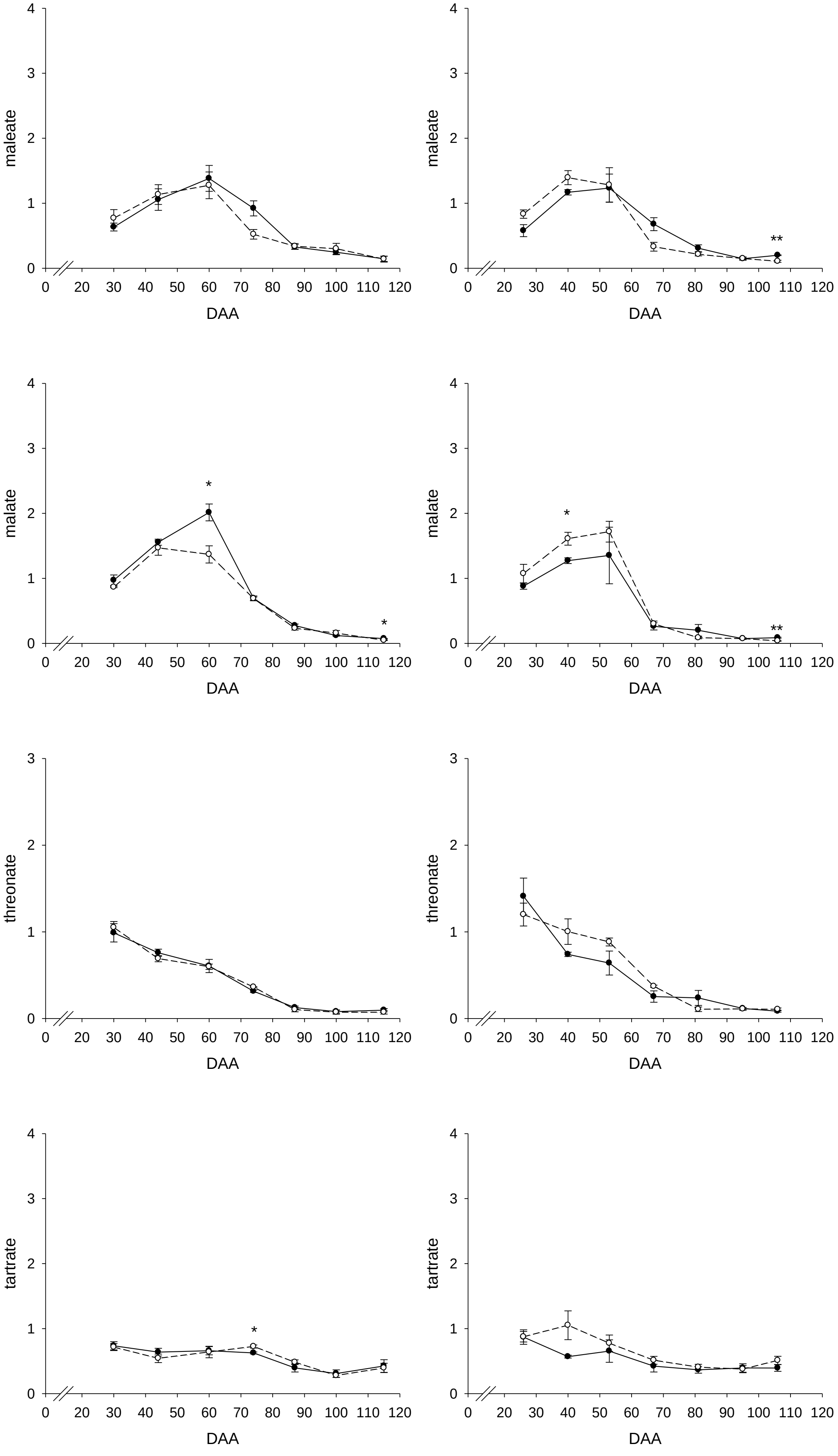

maleate
DAA
maleate
**
DAA
malate
*
*
DAA
malate
*
**
DAA
threonate
DAA
threonate
DAA
tartrate
*
DAA
tartrate
DAA

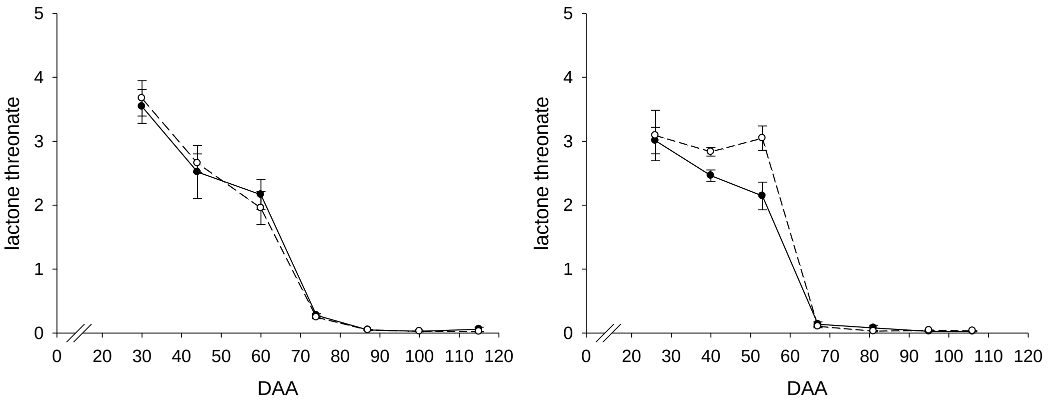
lactone threonate
DAA
0
1
2
3
4
5
0
20
30
40
50
60
70
80
90
100
110
120

Sugars and Polyols

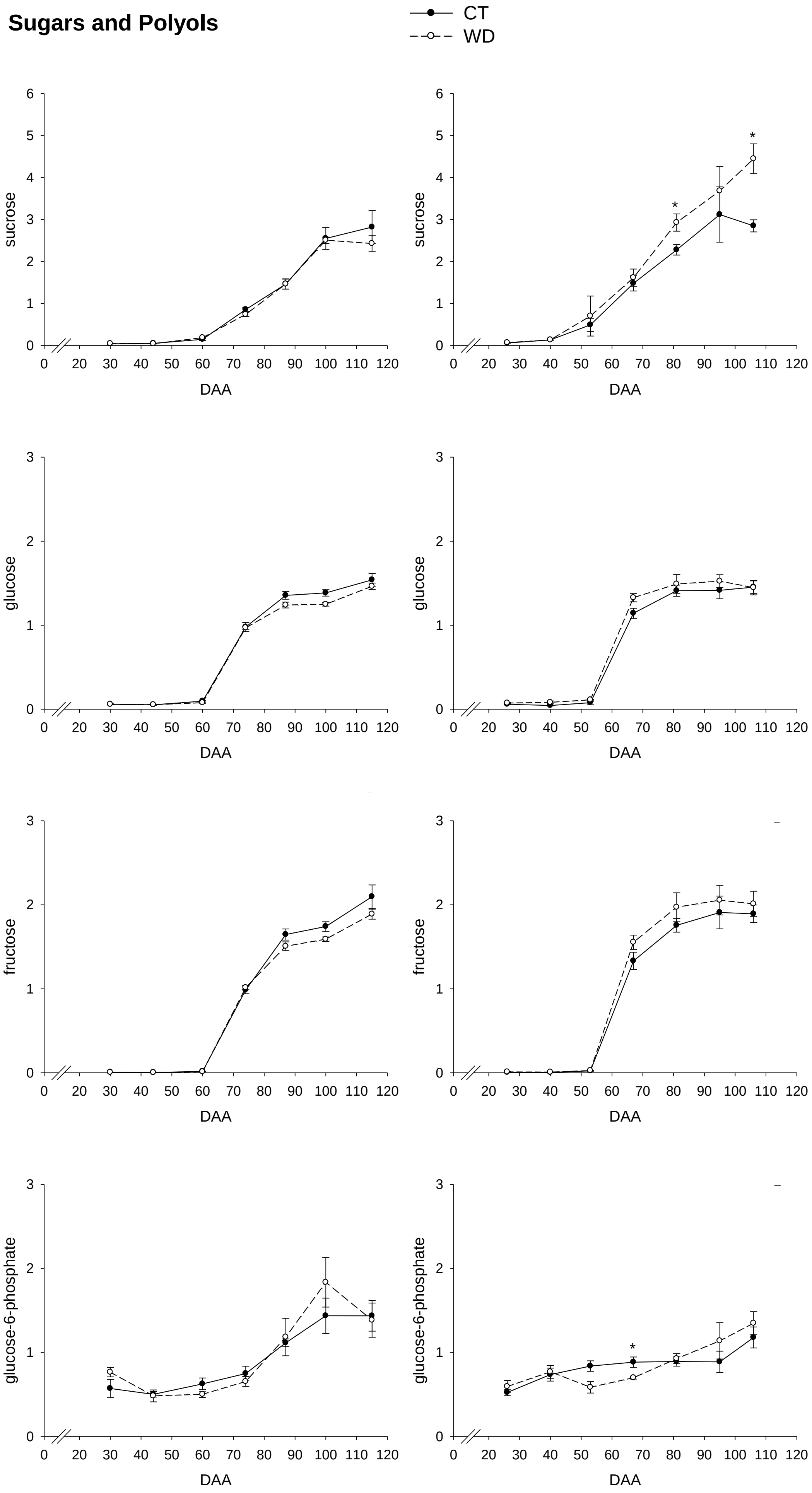

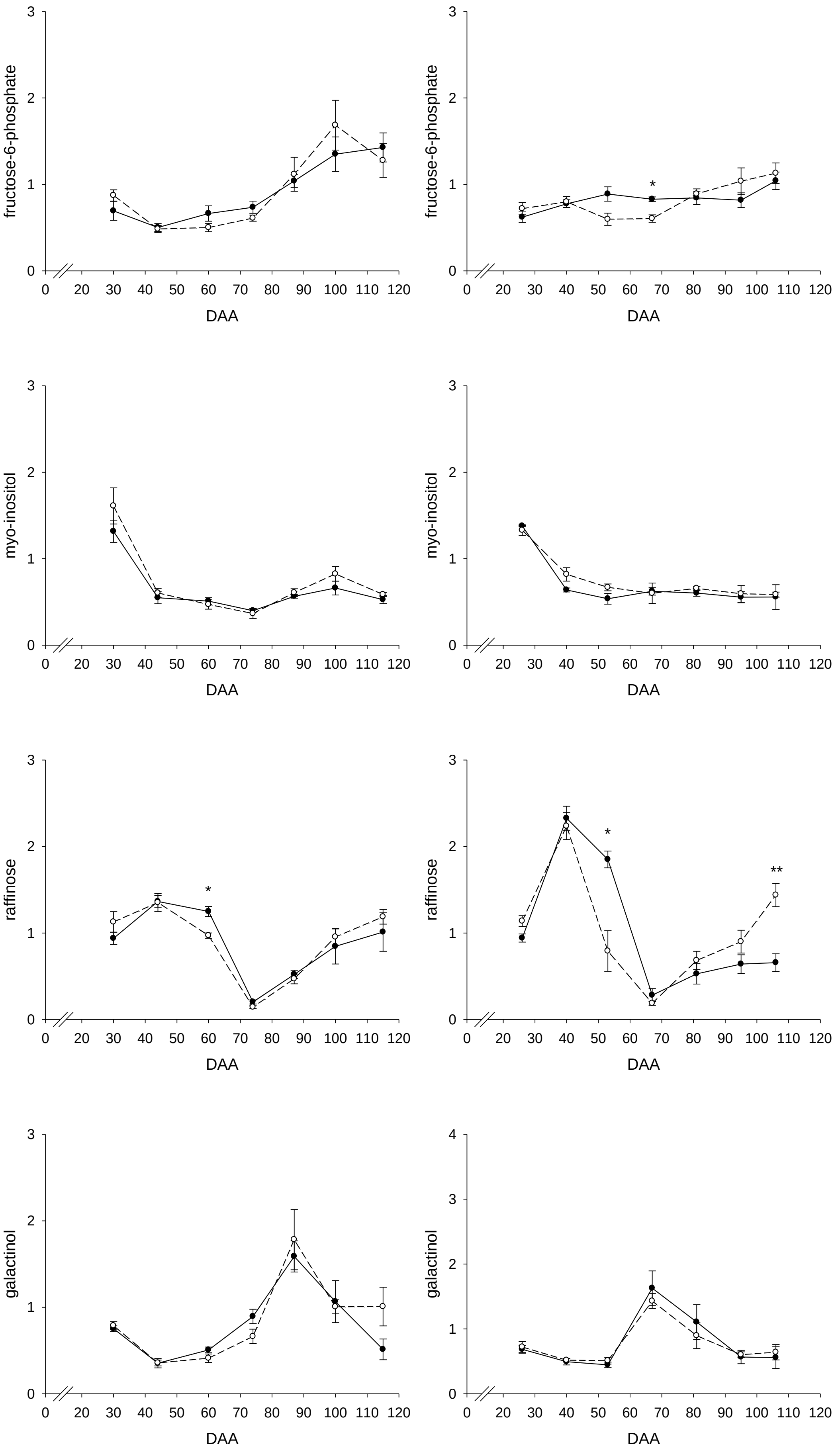

fructose-6-phosphate
DAA
fructose-6-phosphate
DAA
myo-inositol
DAA
myo-inositol
DAA
raffinose
DAA
raffinose
DAA
galactinol
DAA
galactinol
DAA

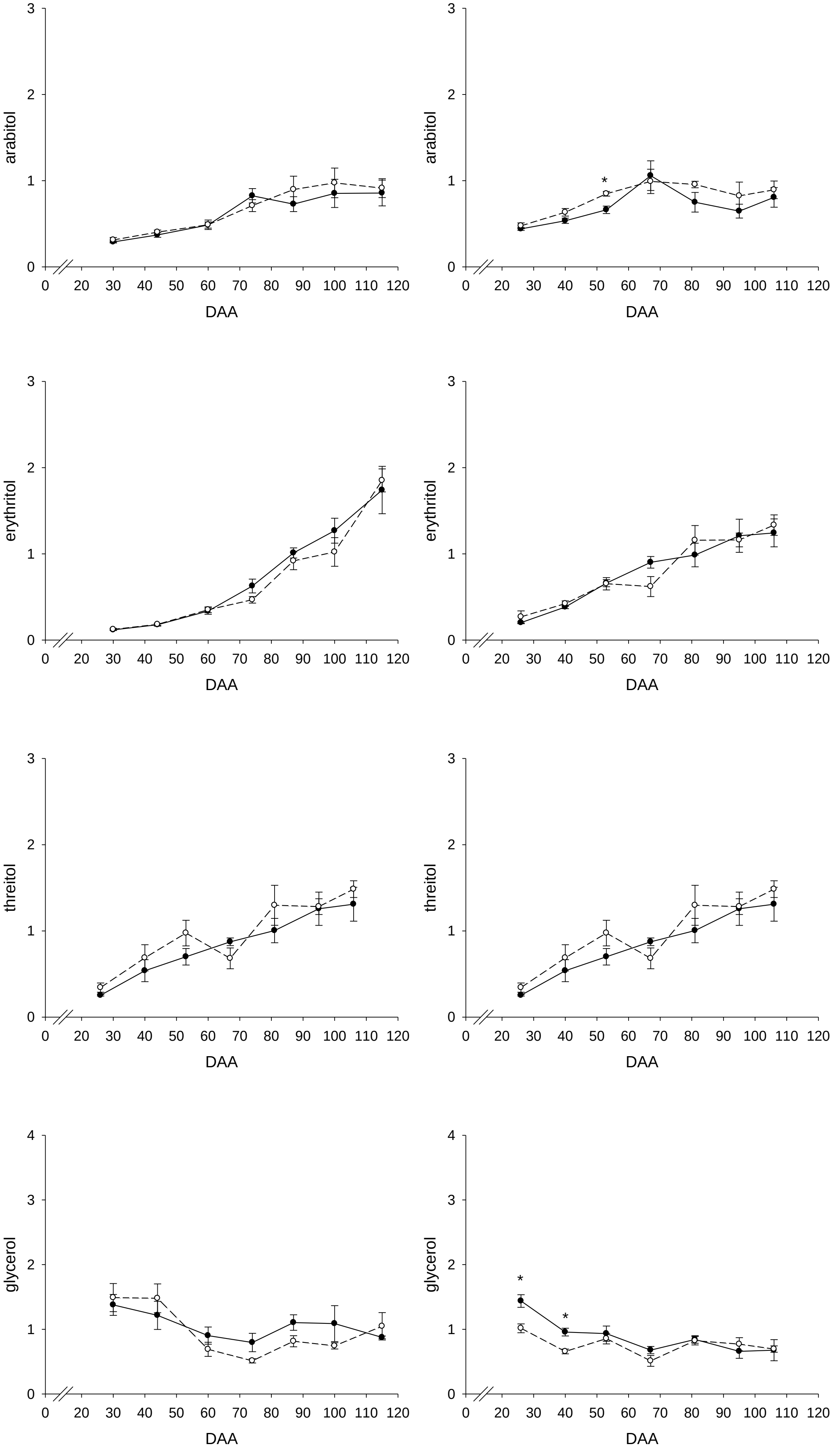

arabitol
arabitol
erythritol
erythritol
threitol
threitol
glycerol
glycerol
DAA
0 20 30 40 50 60 70 80 90 100 110 120
0 1 2 3
0 1 2 3 4
*

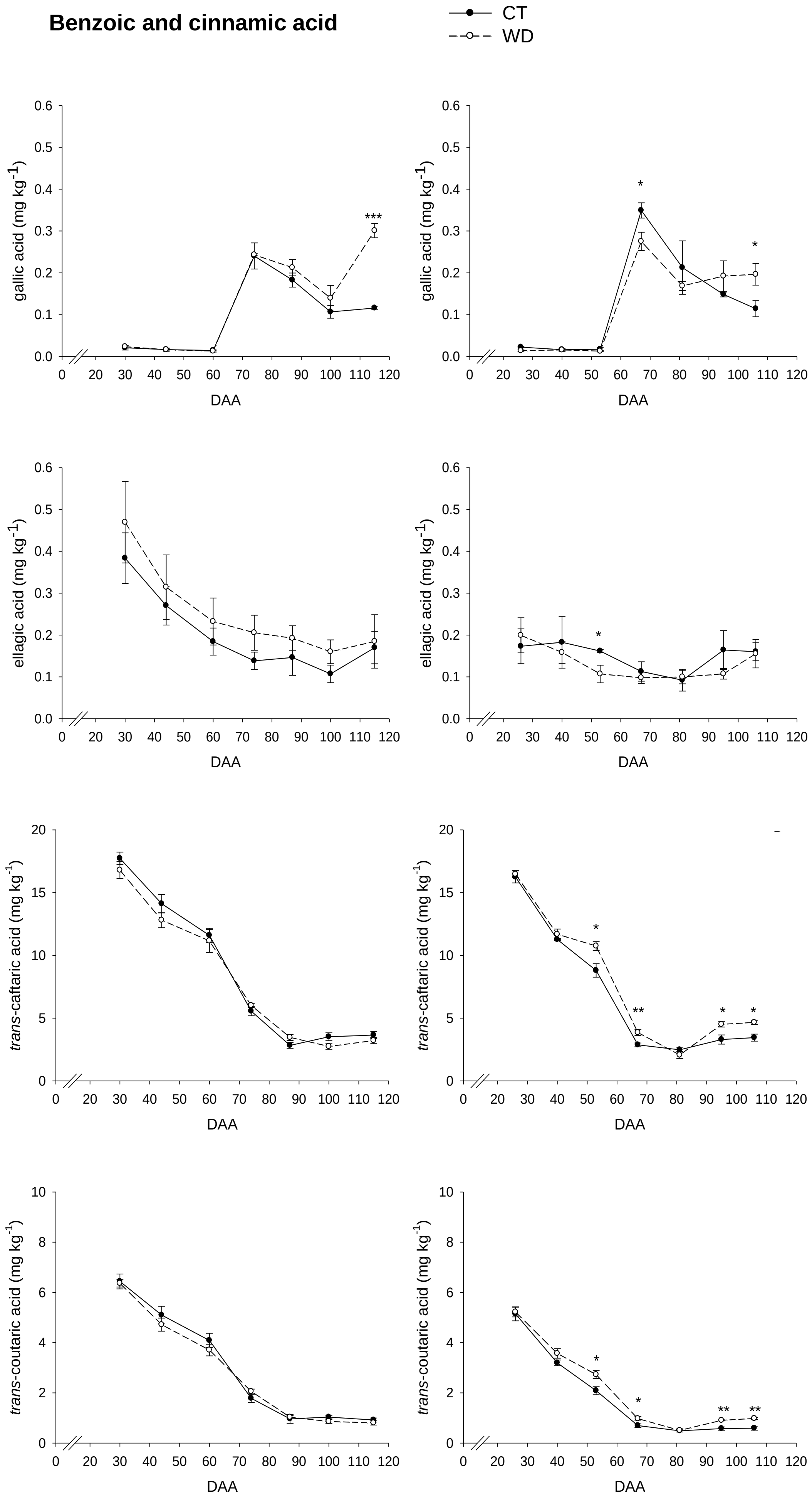

Benzoic and cinnamic acid
CT
WD
gallic acid (mg kg-1)
ellagic acid (mg kg-1)
trans-caftaric acid (mg kg-1)
trans-coutaric acid (mg kg-1)
DAA
*
**
***

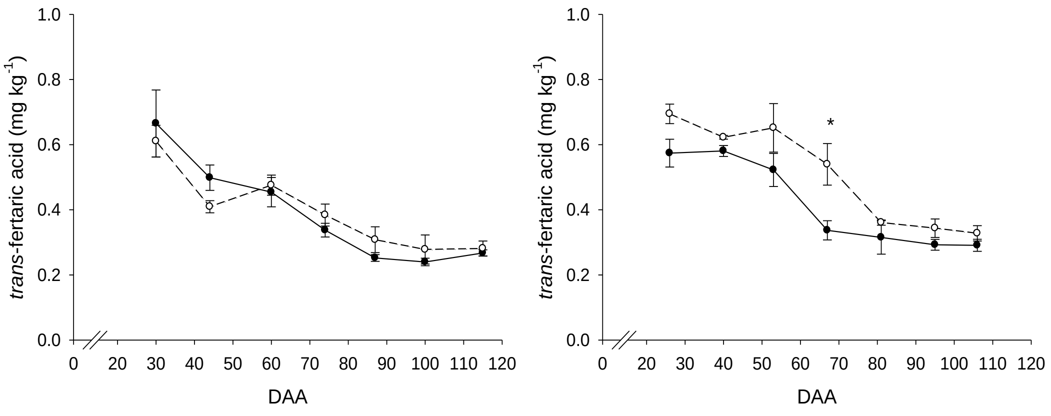

trans-fertaric acid (mg kg-1)
1.0
0.8
0.6
0.4
0.2
0.0
0
20
30
40
50
60
70
80
90
100
110
120
DAA
*

Stilbenoids

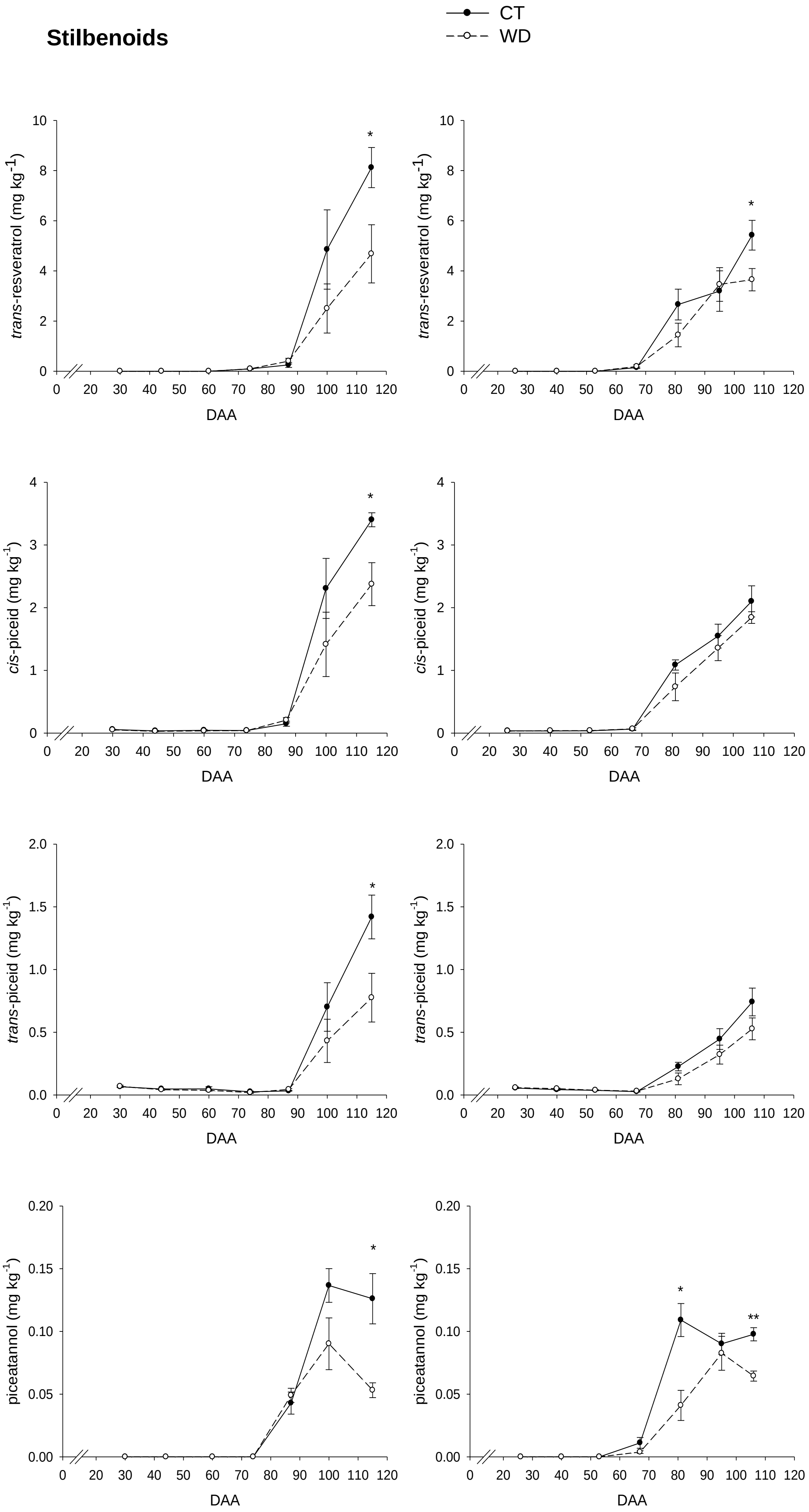

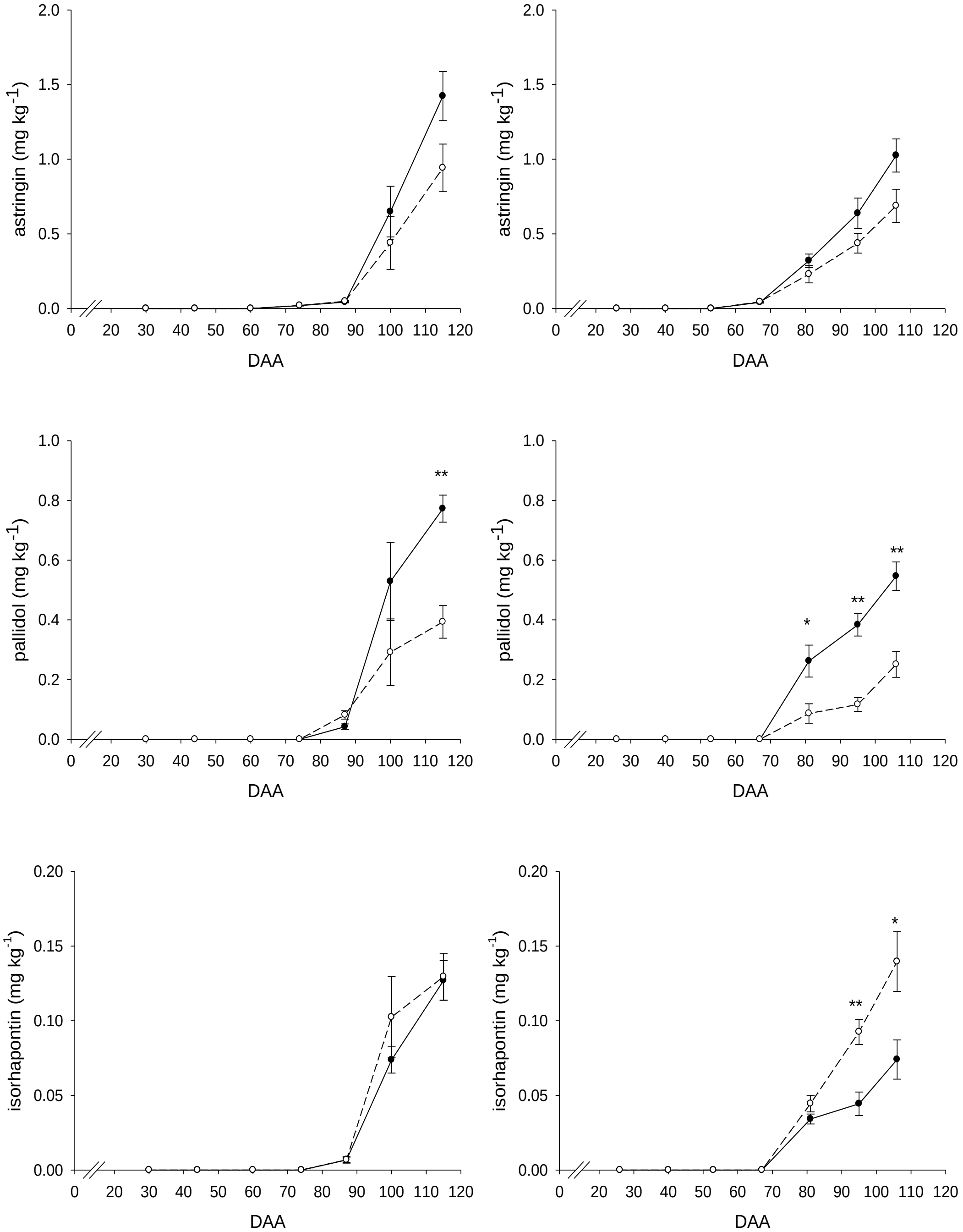

astringin (mg kg-1)
2.0
1.5
1.0
0.5
0.0
0 20 30 40 50 60 70 80 90 100 110 120
DAA
astringin (mg kg-1)
2.0
1.5
1.0
0.5
0.0
0 20 30 40 50 60 70 80 90 100 110 120
DAA
pallidol (mg kg-1)
1.0
0.8
0.6
0.4
0.2
0.0
**
0 20 30 40 50 60 70 80 90 100 110 120
DAA
pallidol (mg kg-1)
1.0
0.8
0.6
0.4
0.2
0.0
*
**
**
0 20 30 40 50 60 70 80 90 100 110 120
DAA
isorhapontin (mg kg-1)
0.20
0.15
0.10
0.05
0.00
0 20 30 40 50 60 70 80 90 100 110 120
DAA
isorhapontin (mg kg-1)
0.20
0.15
0.10
0.05
0.00
**
*
0 20 30 40 50 60 70 80 90 100 110 120
DAA

Dihydrochalcones

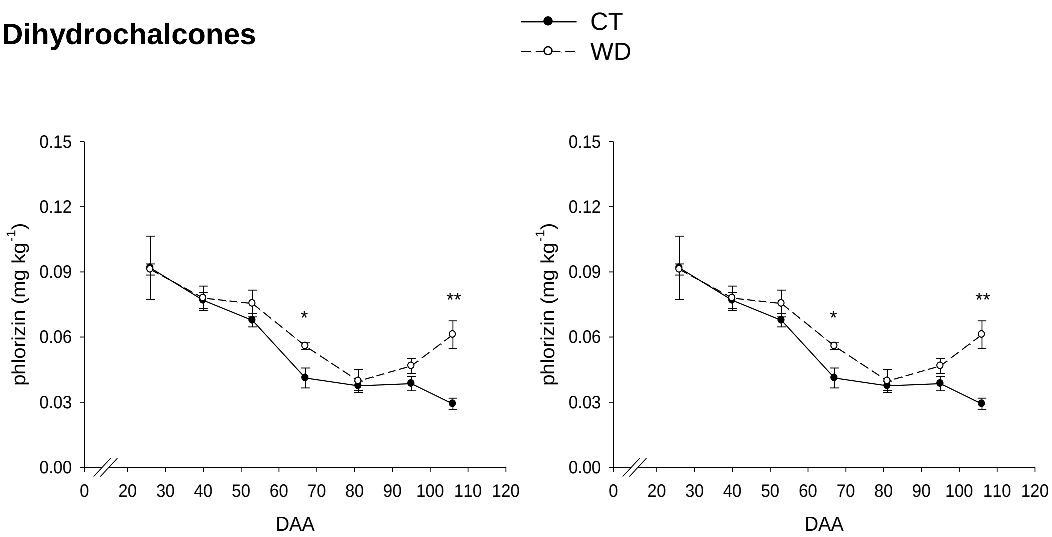

Flavan-3-ols

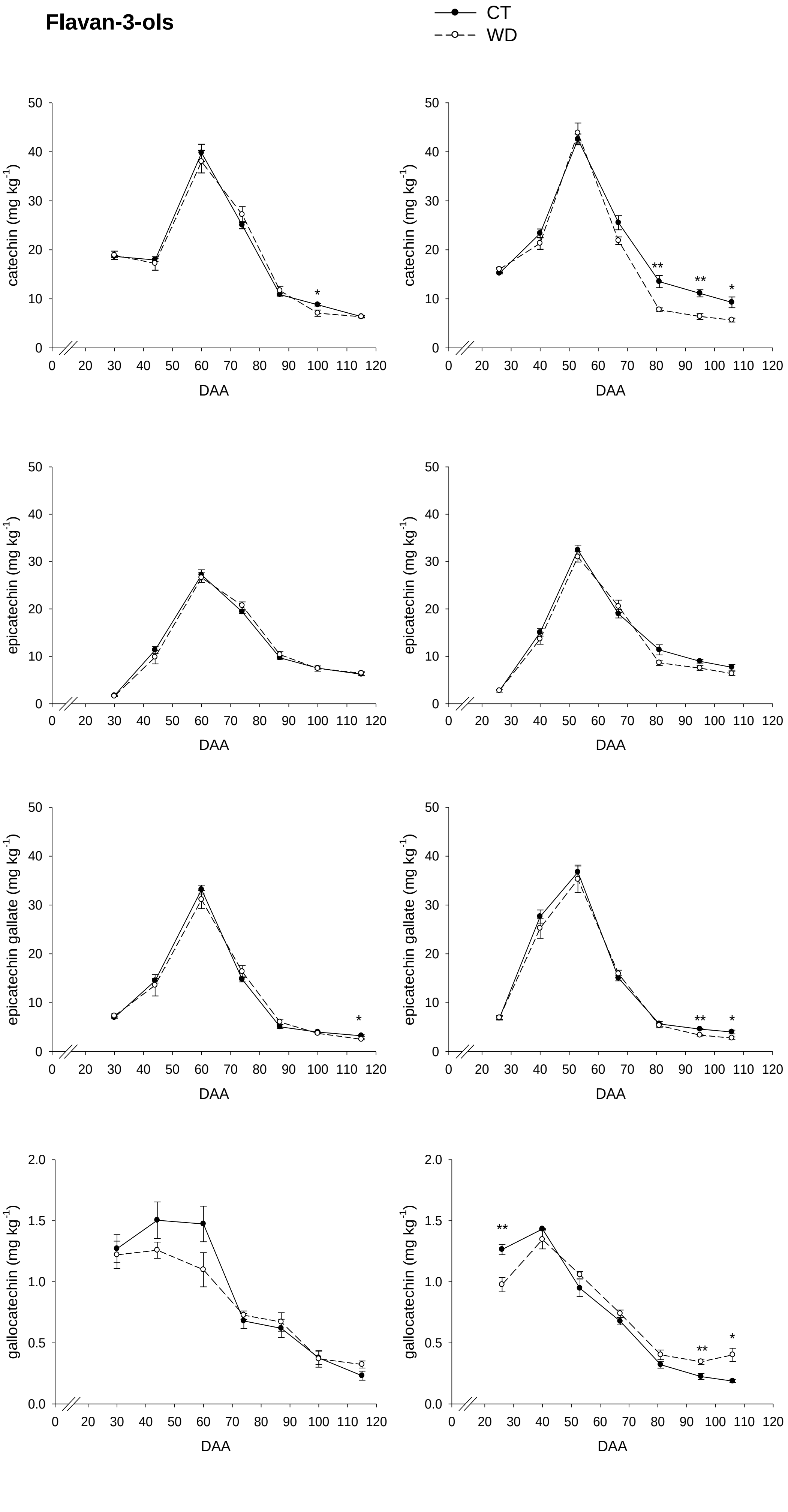

CT
WD
catechin (mg kg^{-1})
epicatechin (mg kg^{-1})
epicatechin gallate (mg kg^{-1})
gallocatechin (mg kg^{-1})
DAA
0
10
20
30
40
50
0.0
0.5
1.0
1.5
2.0
0 20 30 40 50 60 70 80 90 100 110 120
*
**

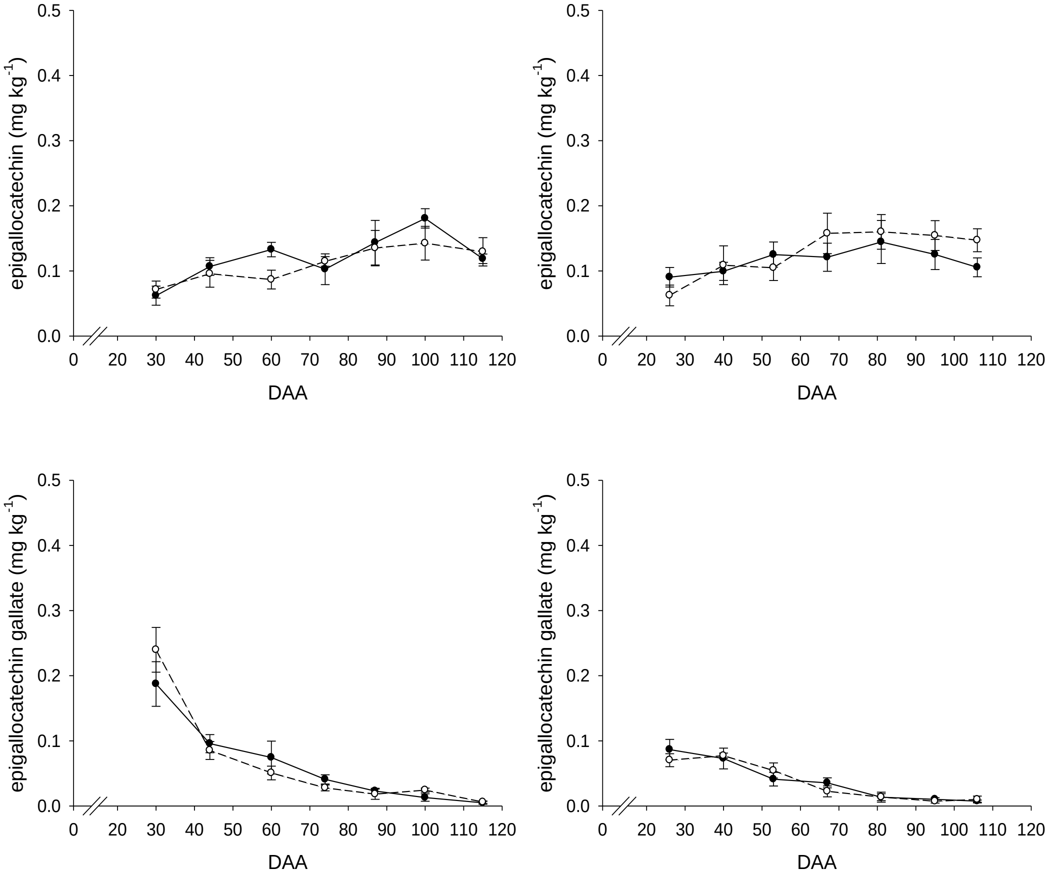

epigallocatechin (mg kg^{-1})
epigallocatechin gallate (mg kg^{-1})
0.0
0.1
0.2
0.3
0.4
0.5
0 20 30 40 50 60 70 80 90 100 110 120
DAA

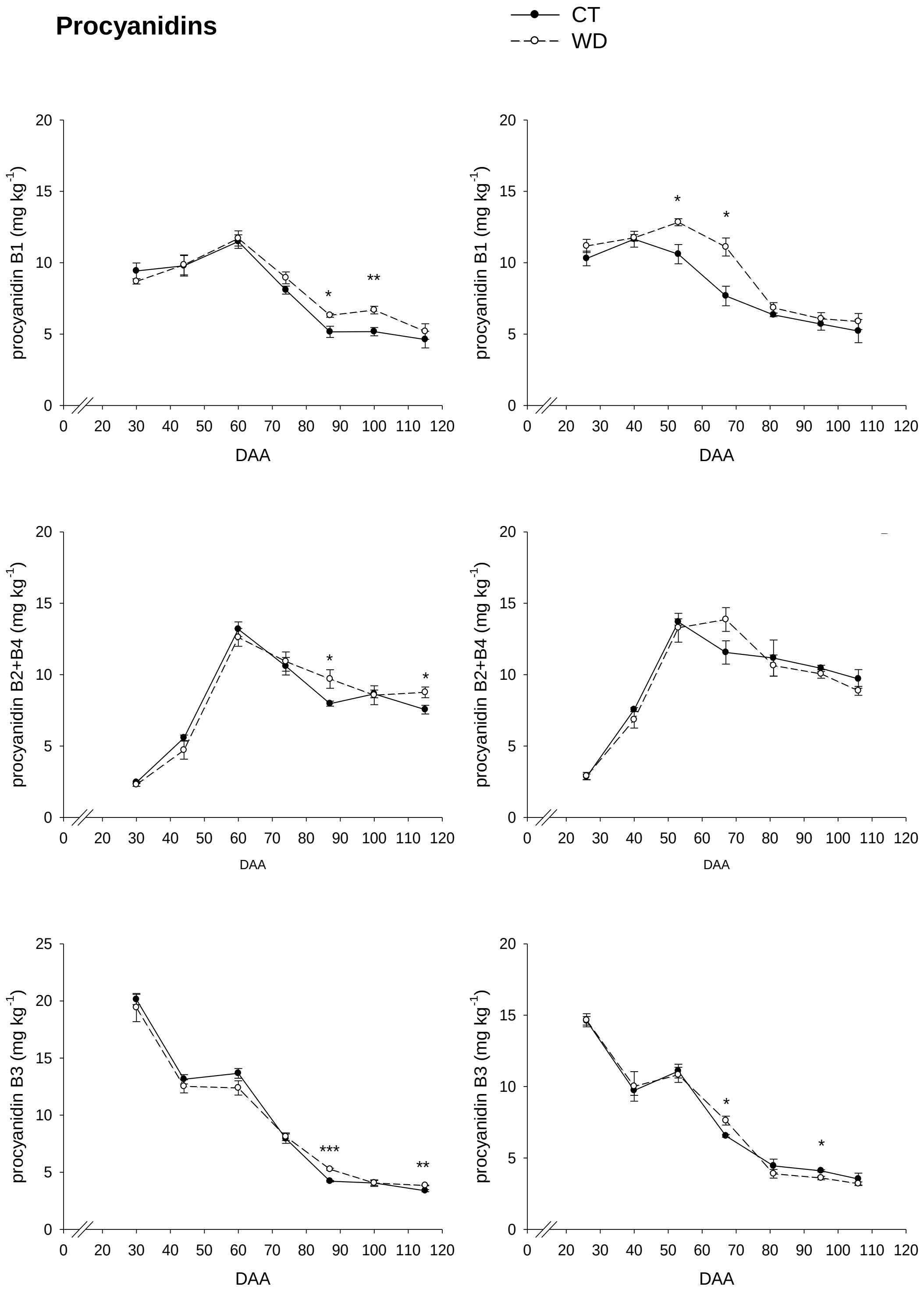
Procyanidins
CT
WD
procyanidin B1 (mg kg-1)
procyanidin B2+B4 (mg kg-1)
procyanidin B3 (mg kg-1)
DAA
0
20
30
40
50
60
70
80
90
100
110
120
5
10
15
20
25
*
**
***

Flavonols

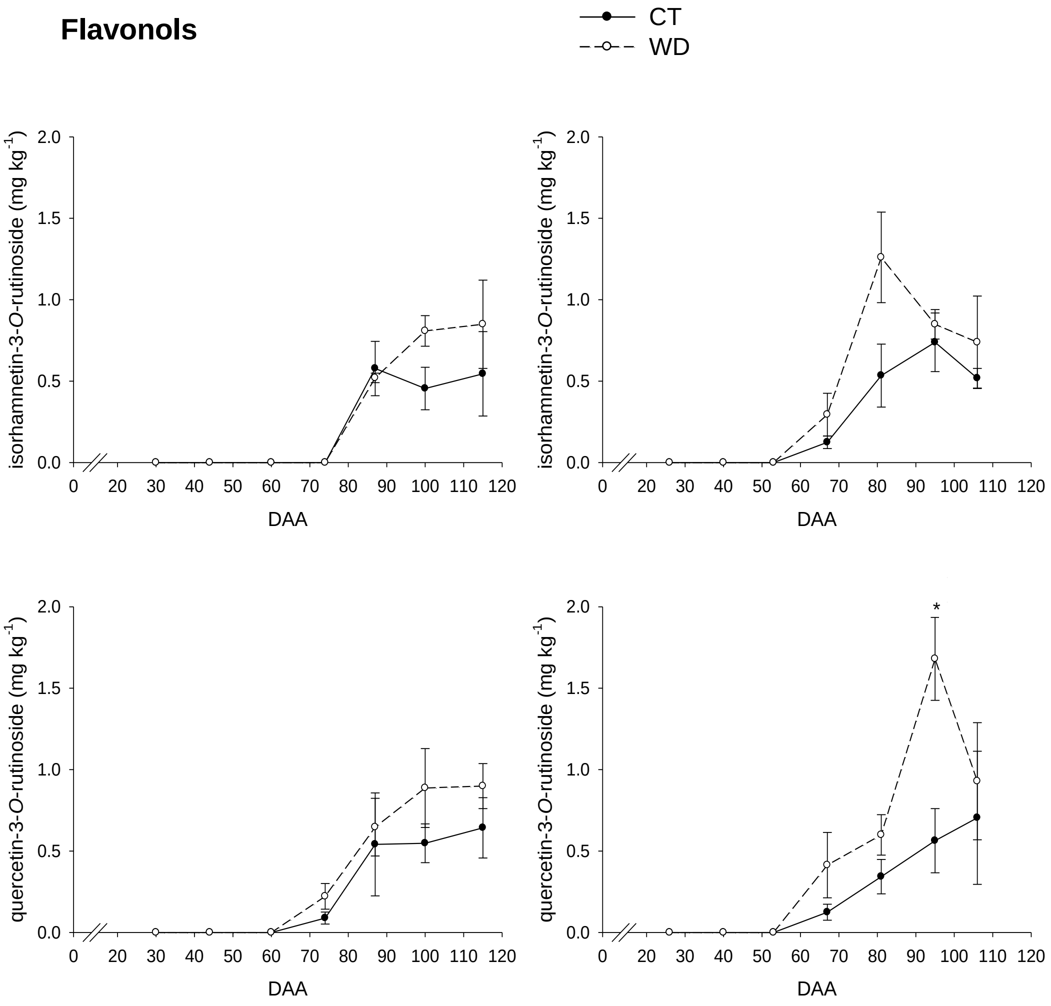
CT
WD
isorhamnetin-3-O-rutinoside (mg kg-1)
quercetin-3-O-rutinoside (mg kg-1)
DAA
2.0
1.5
1.0
0.5
0.0
0
20
30
40
50
60
70
80
90
100
110
120
*

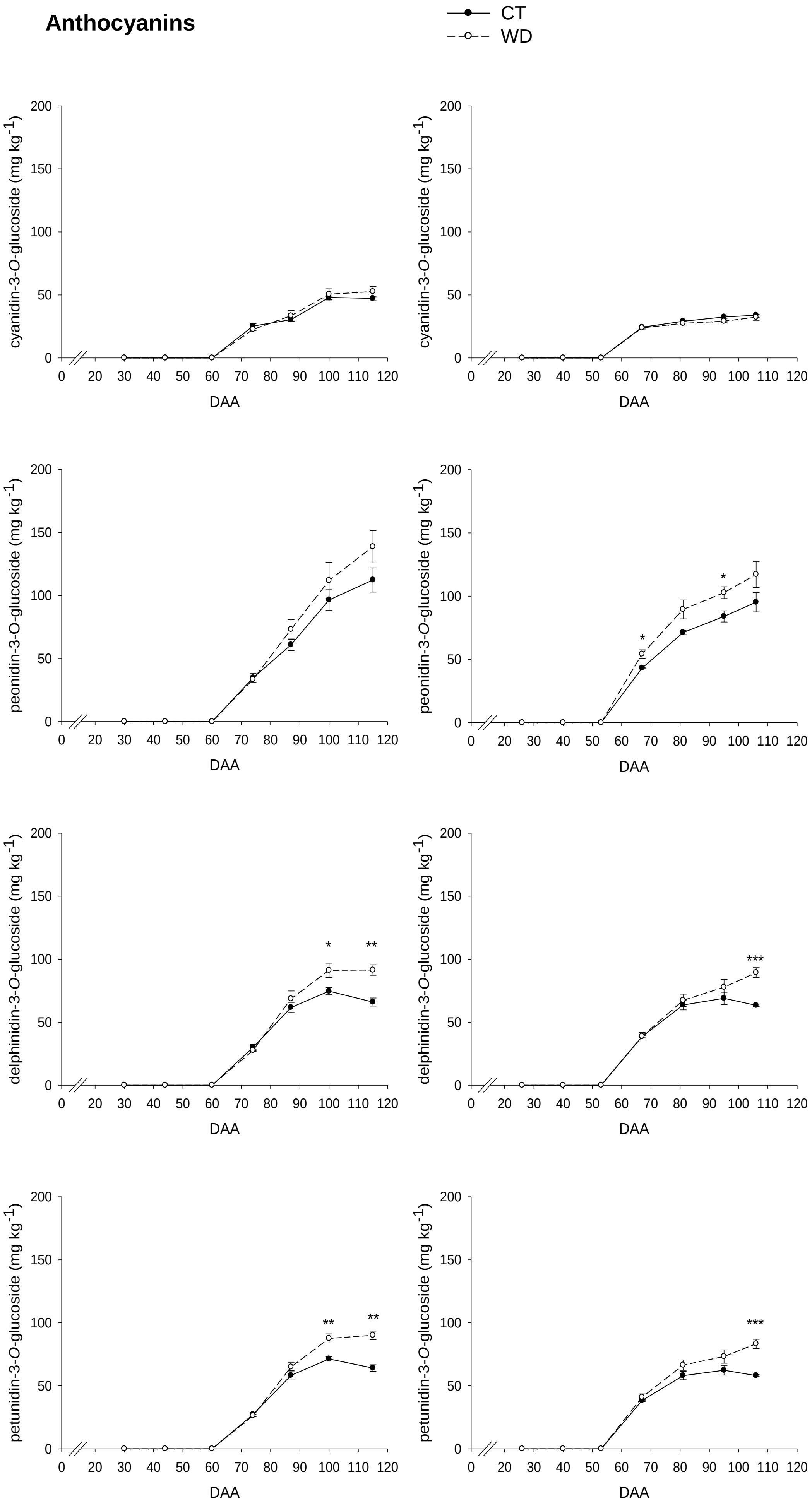
Anthocyanins
CT
WD
cyanidin-3-O-glucoside (mg kg-1)
peonidin-3-O-glucoside (mg kg-1)
delphinidin-3-O-glucoside (mg kg-1)
petunidin-3-O-glucoside (mg kg-1)
DAA
0 20 30 40 50 60 70 80 90 100 110 120
0 50 100 150 200
*
**
***

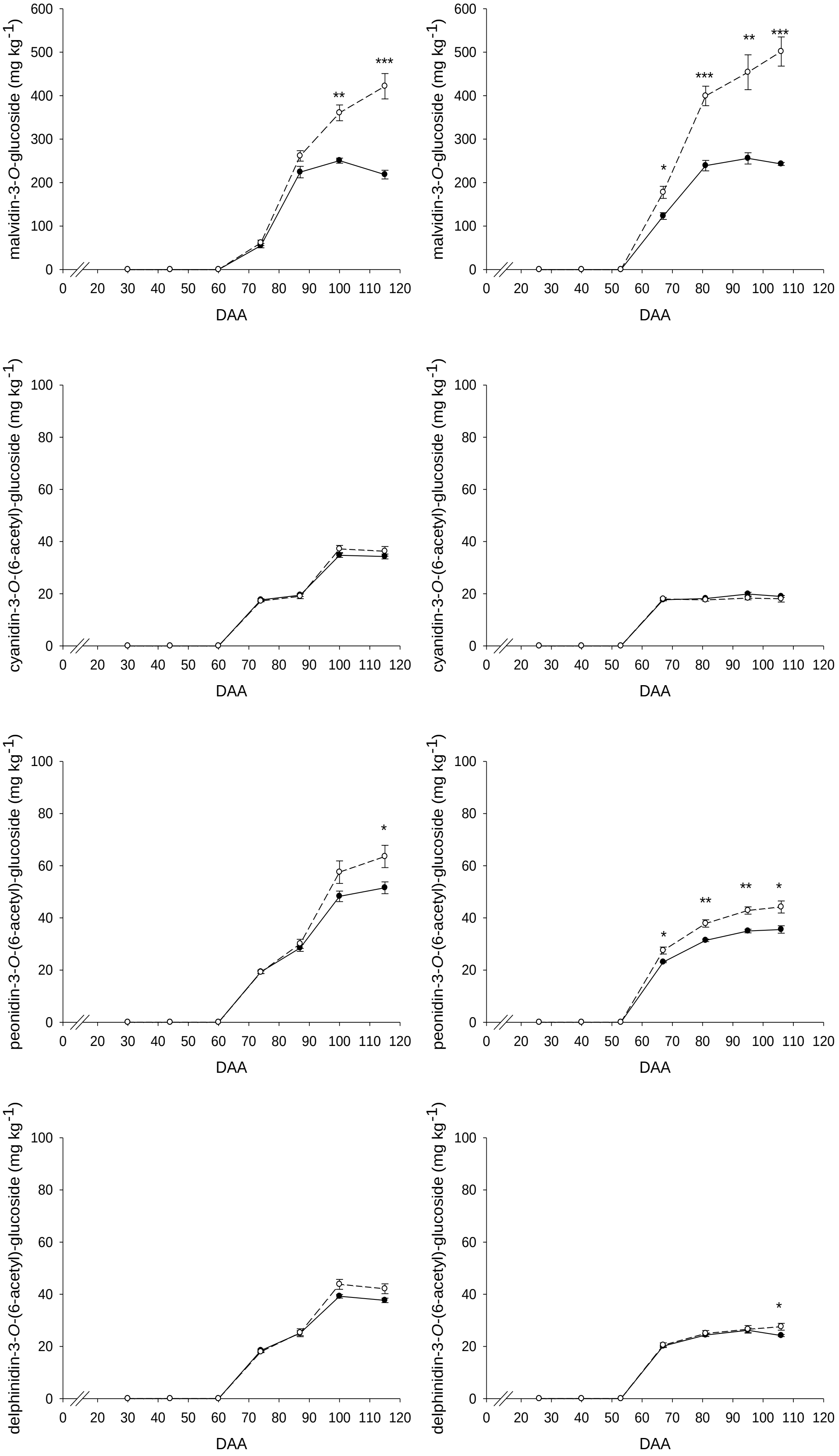

malvidin-3-O-glucoside (mg kg-1)
cyanidin-3-O-(6-acetyl)-glucoside (mg kg-1)
peonidin-3-O-(6-acetyl)-glucoside (mg kg-1)
delphinidin-3-O-(6-acetyl)-glucoside (mg kg-1)
DAA

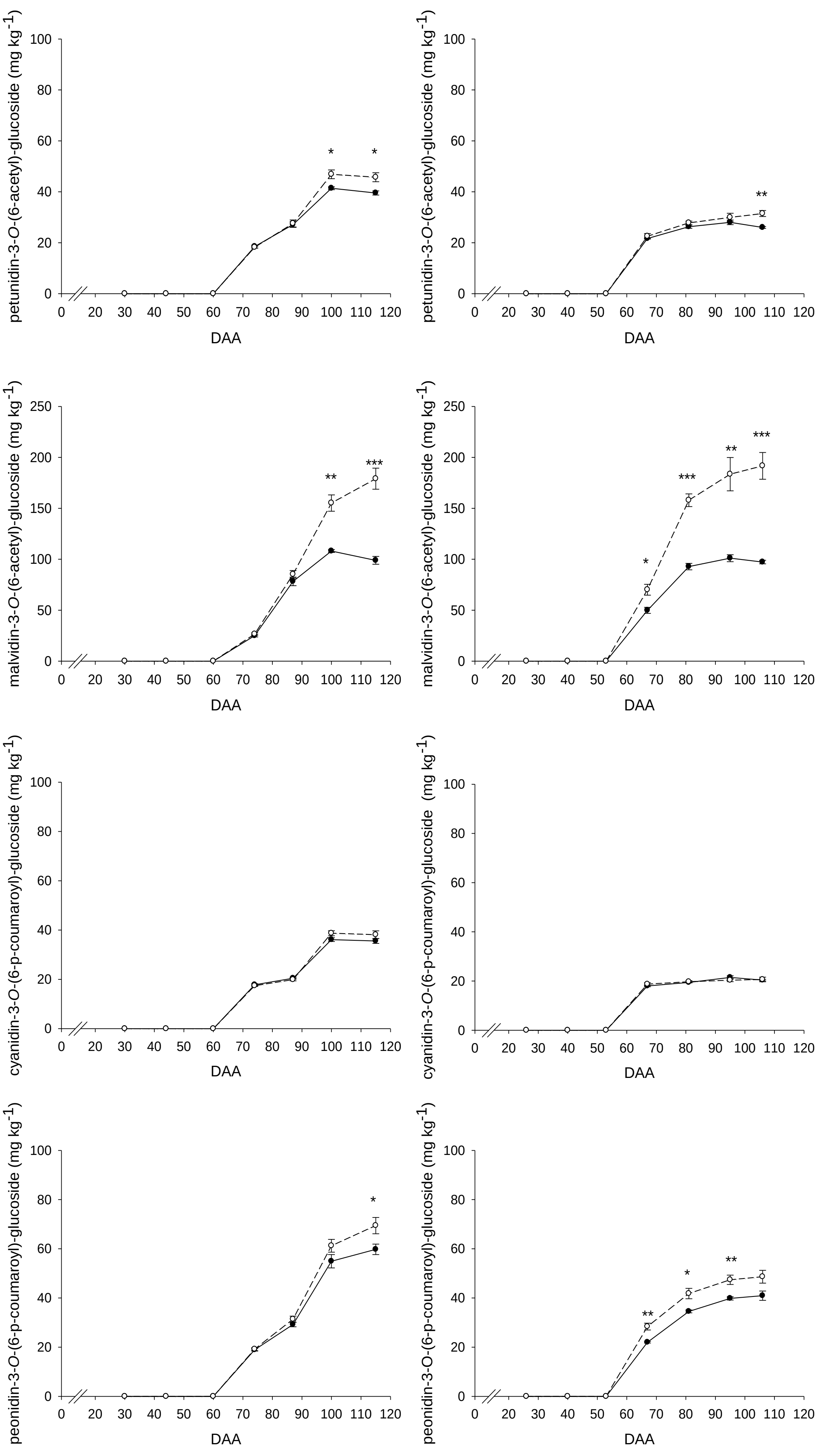

petunidin-3-O-(6-acetyl)-glucoside (mg kg-1)
DAA
*
*
petunidin-3-O-(6-acetyl)-glucoside (mg kg-1)
DAA
**
malvidin-3-O-(6-acetyl)-glucoside (mg kg-1)
DAA
**
***
malvidin-3-O-(6-acetyl)-glucoside (mg kg-1)
DAA
*
***
**
***
cyanidin-3-O-(6-p-coumaroyl)-glucoside (mg kg-1)
DAA
cyanidin-3-O-(6-p-coumaroyl)-glucoside (mg kg-1)
DAA
peonidin-3-O-(6-p-coumaroyl)-glucoside (mg kg-1)
DAA
*
peonidin-3-O-(6-p-coumaroyl)-glucoside (mg kg-1)
DAA
**
*
**

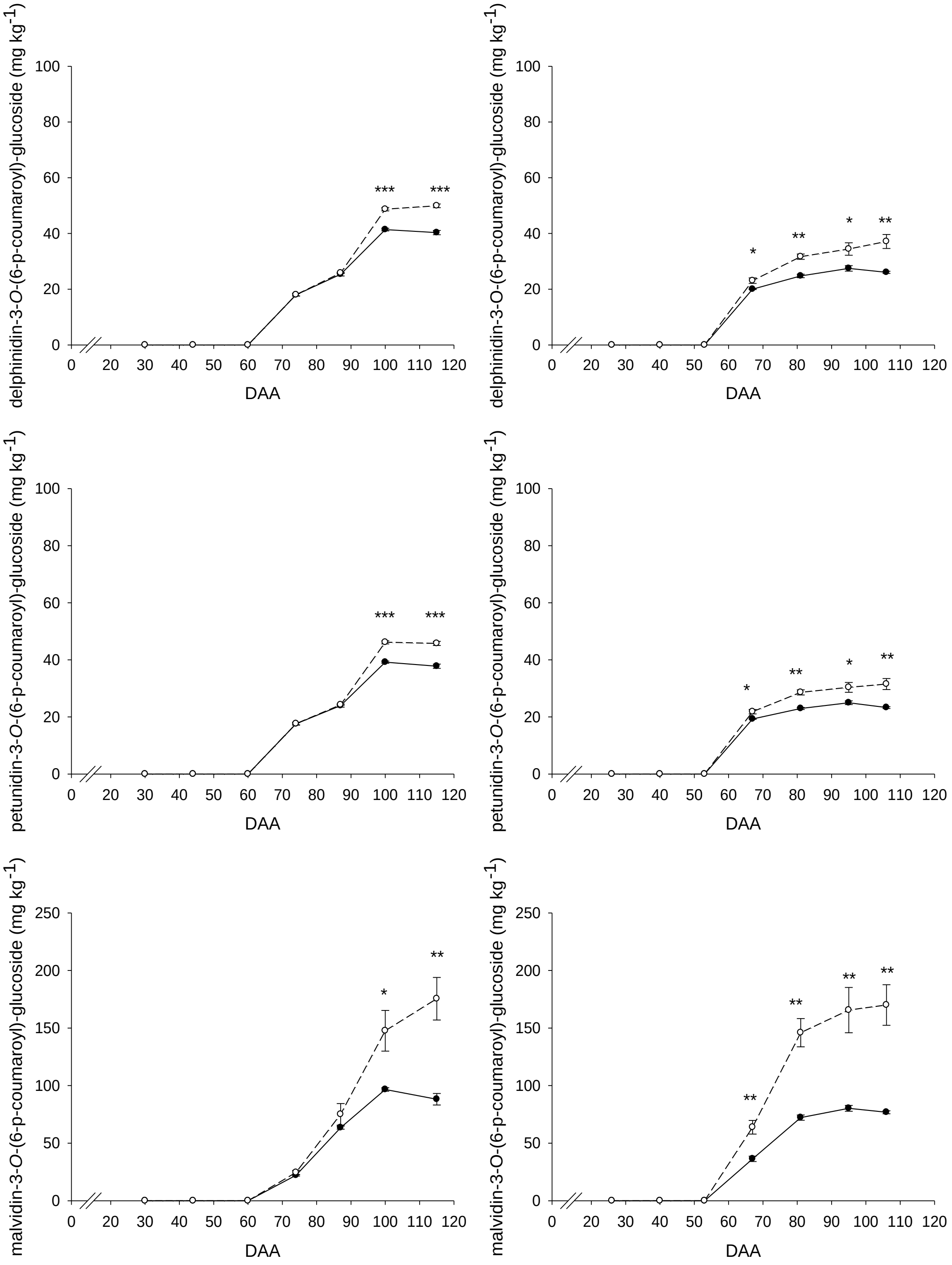
delphinidin-3-O-(6-p-coumaroyl)-glucoside (mg kg-1)
petunidin-3-O-(6-p-coumaroyl)-glucoside (mg kg-1)
malvidin-3-O-(6-p-coumaroyl)-glucoside (mg kg-1)
DAA
0 20 30 40 50 60 70 80 90 100 110 120
0 20 40 60 80 100
0 50 100 150 200 250
***
**
*

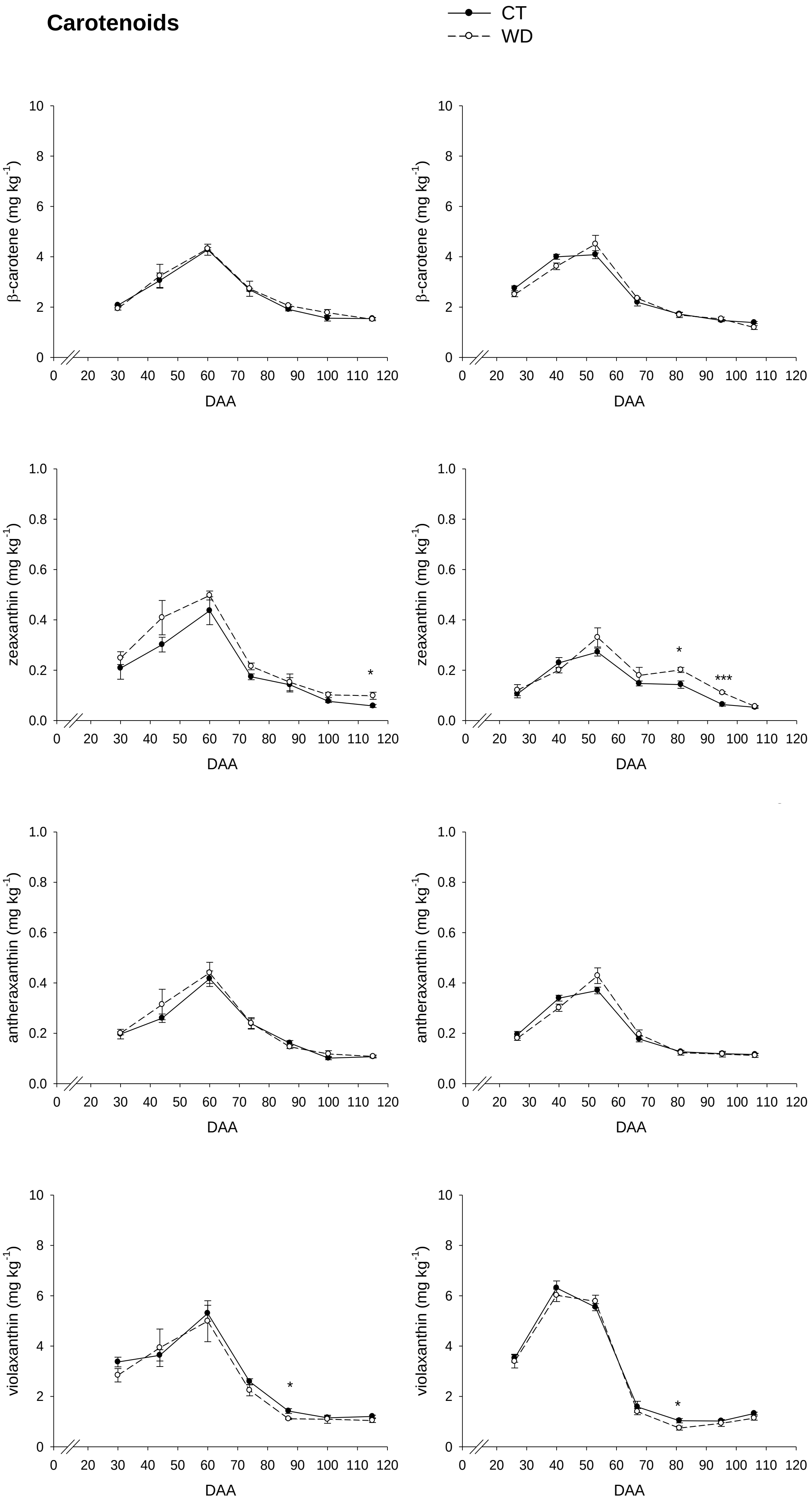
Carotenoids
CT
WD
β-carotene (mg kg-1)
zeaxanthin (mg kg-1)
antheraxanthin (mg kg-1)
violaxanthin (mg kg-1)
DAA
*
***

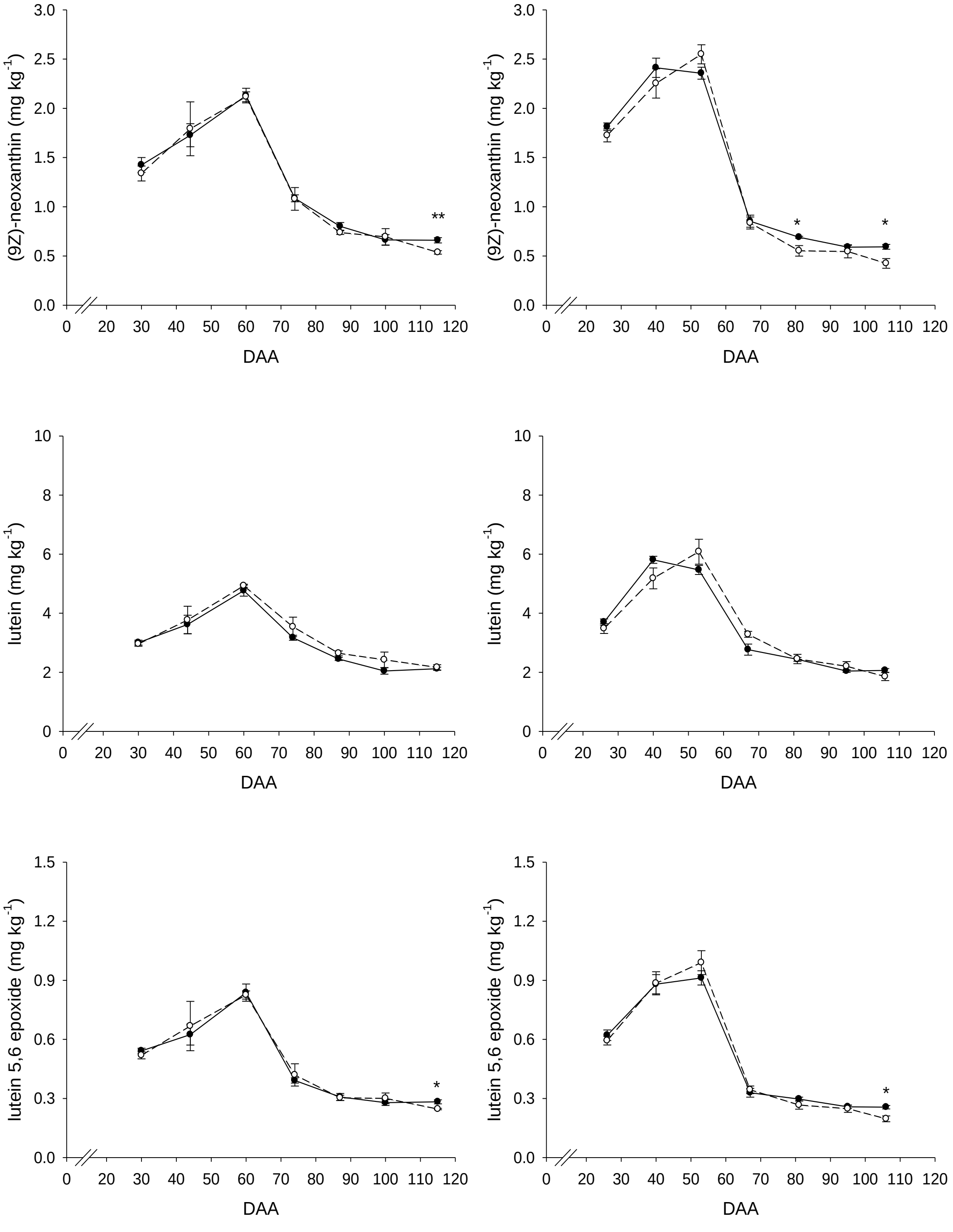

(9Z)-neoxanthin (mg kg-1)
0.0
0.5
1.0
1.5
2.0
2.5
3.0
0 20 30 40 50 60 70 80 90 100 110 120
DAA
**
(9Z)-neoxanthin (mg kg-1)
0 20 30 40 50 60 70 80 90 100 110 120
DAA
*
*
lutein (mg kg-1)
0
2
4
6
8
10
0 20 30 40 50 60 70 80 90 100 110 120
DAA
lutein (mg kg-1)
0 20 30 40 50 60 70 80 90 100 110 120
DAA
lutein 5,6 epoxide (mg kg-1)
0.0
0.3
0.6
0.9
1.2
1.5
0 20 30 40 50 60 70 80 90 100 110 120
DAA
*
lutein 5,6 epoxide (mg kg-1)
0 20 30 40 50 60 70 80 90 100 110 120
DAA
*

C13 Norisoprenoids

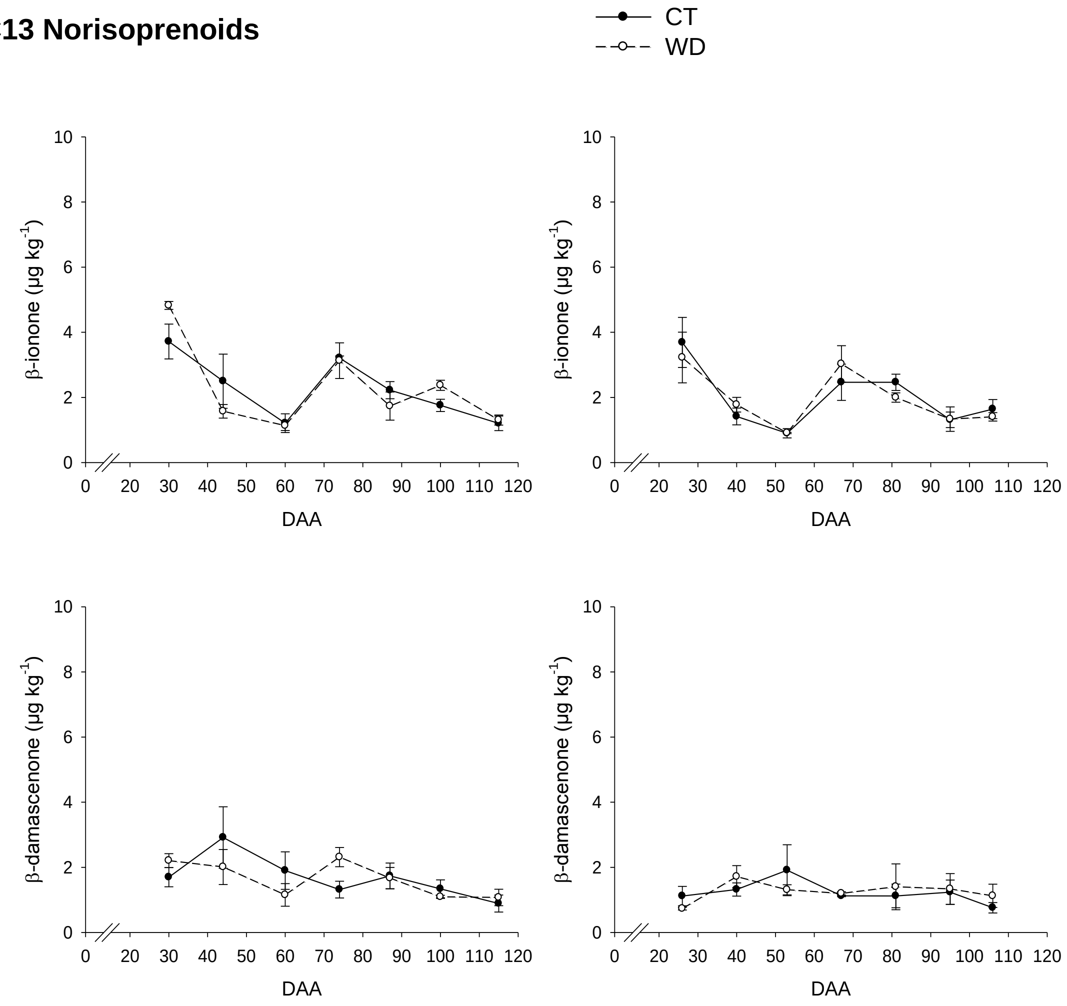

C6 and C5 volatile organic compounds

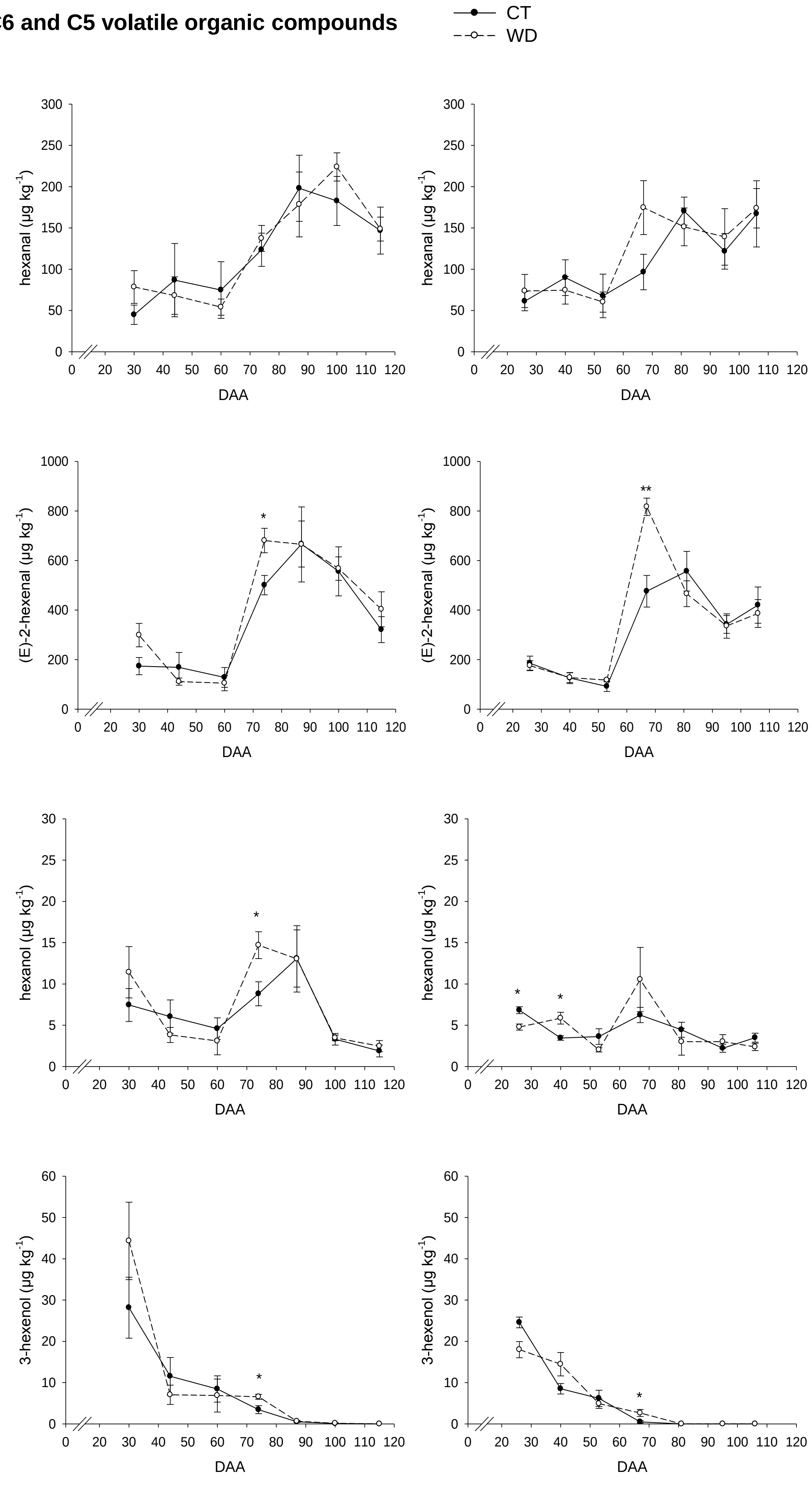

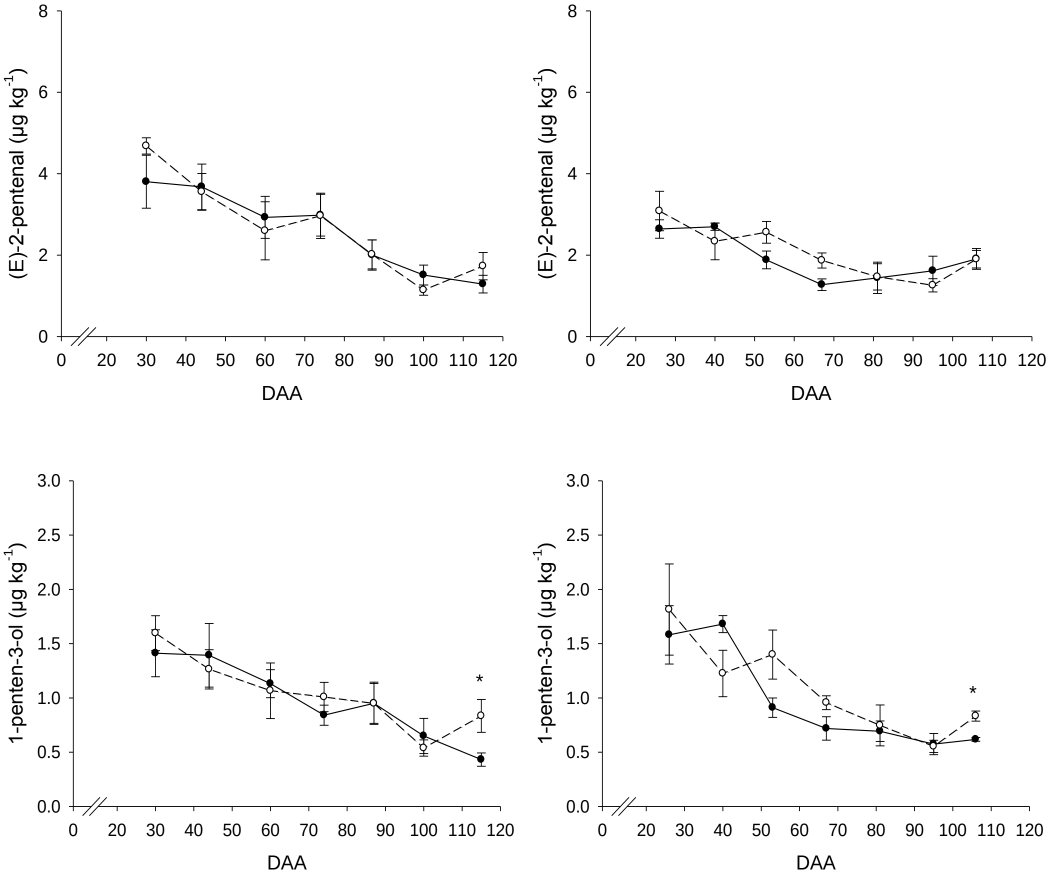

(E)-2-pentenal (µg kg-1)
1-penten-3-ol (µg kg-1)
DAA
0 20 30 40 50 60 70 80 90 100 110 120
0 2 4 6 8
0.0 0.5 1.0 1.5 2.0 2.5 3.0
*

C7, C8 and C9 volatile organic compounds

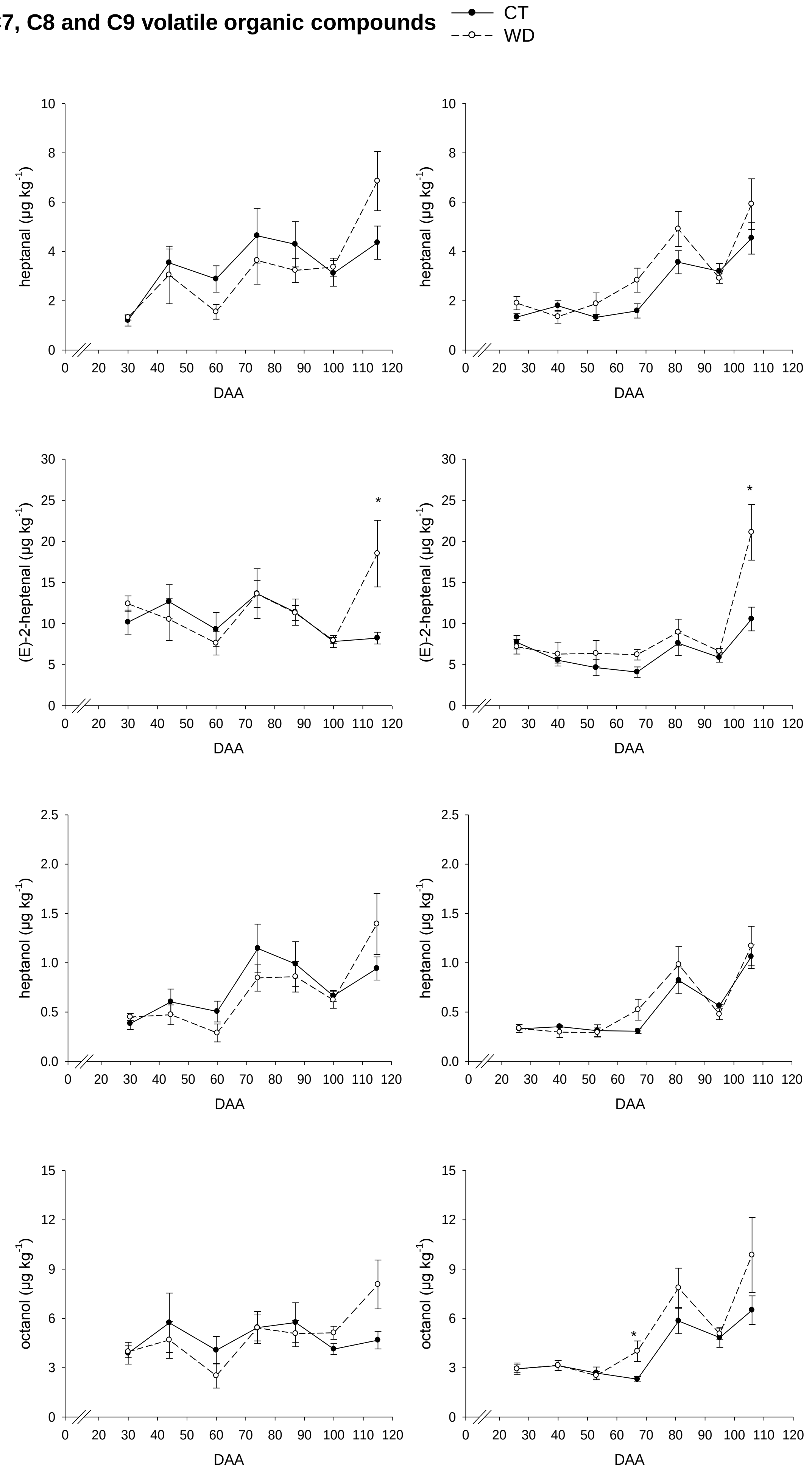

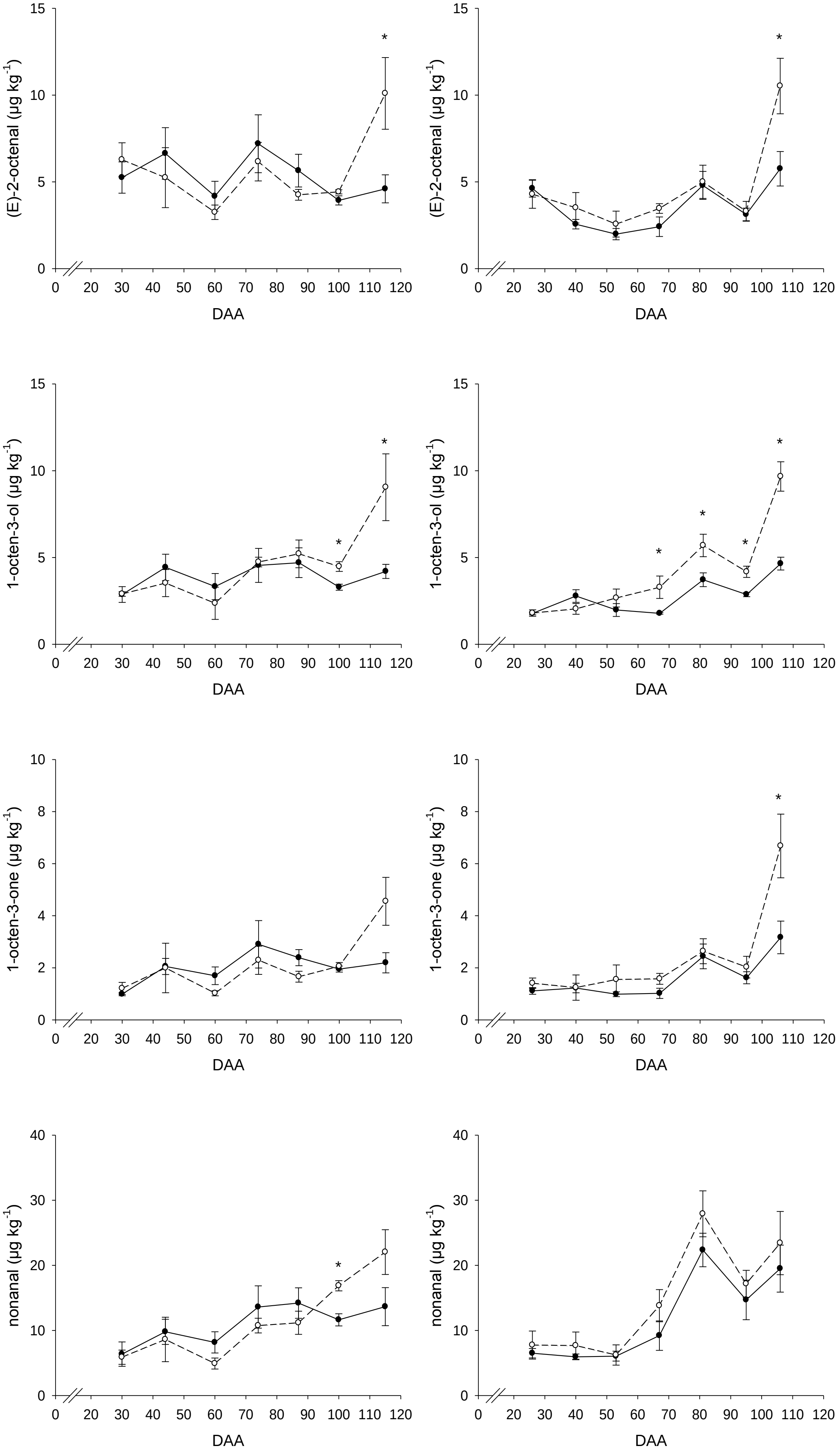

(E)-2-octenal (μg kg-1)
DAA
(E)-2-octenal (μg kg-1)
DAA
1-octen-3-ol (μg kg-1)
DAA
1-octen-3-ol (μg kg-1)
DAA
1-octen-3-one (μg kg-1)
DAA
1-octen-3-one (μg kg-1)
DAA
nonanal (μg kg-1)
DAA
nonanal (μg kg-1)
DAA

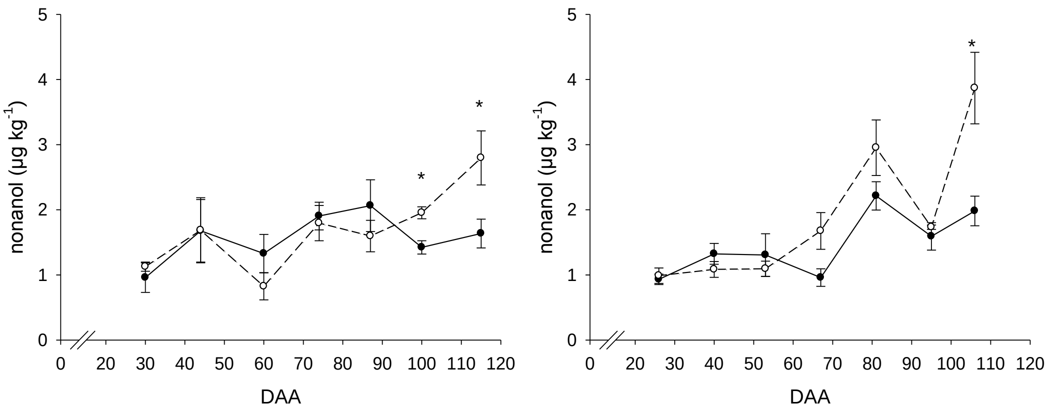

nonanol (µg kg-1)
DAA
*
*
nonanol (µg kg-1)
DAA
*

Terpenes

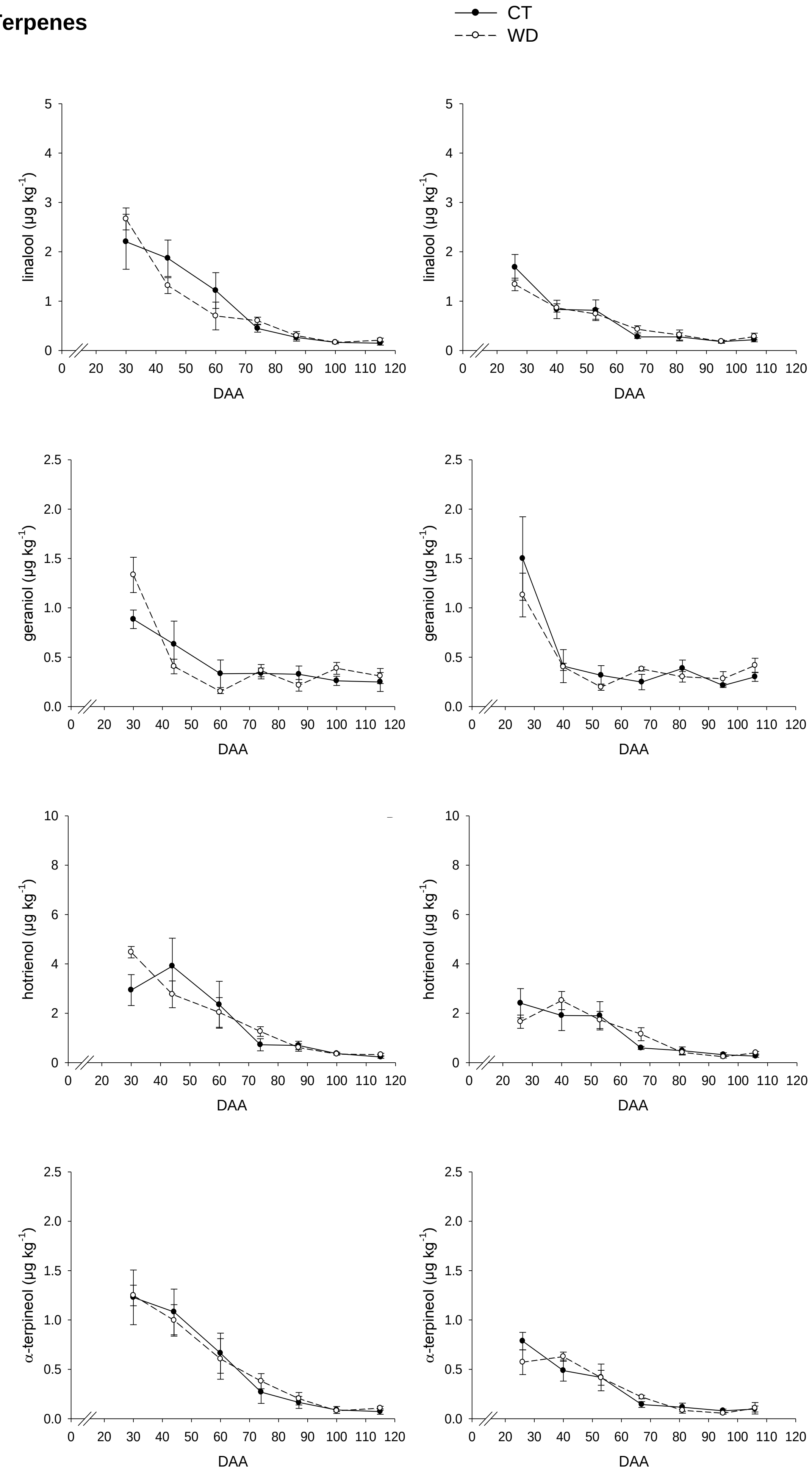
CT
WD
linalool (μg kg-1)
geraniol (μg kg-1)
hotrienol (μg kg-1)
α-terpineol (μg kg-1)
DAA

Supplementary FigureS1. Central and specialized metabolites concentration in CT and WD berries during fruit development in 2011 (left panels) and 2012 (right panels).
