# Supplementary material for: Multi-Omics and Integrated Network Analyses Reveal New Insights into the Systems Relationships between Metabolites, Structural Genes, and Transcriptional Regulators in Developing Grape Berries (Vitis vinifera L.) Exposed to Water Deficit
Source: Front Plant Sci. 2017 Jul 10;8:1124. doi: 10.3389/fpls.2017.01124 (PMC5502274; doi:10.3389/fpls.2017.01124)
Supplement: Supplementary file 8 [file Image_2.PDF]

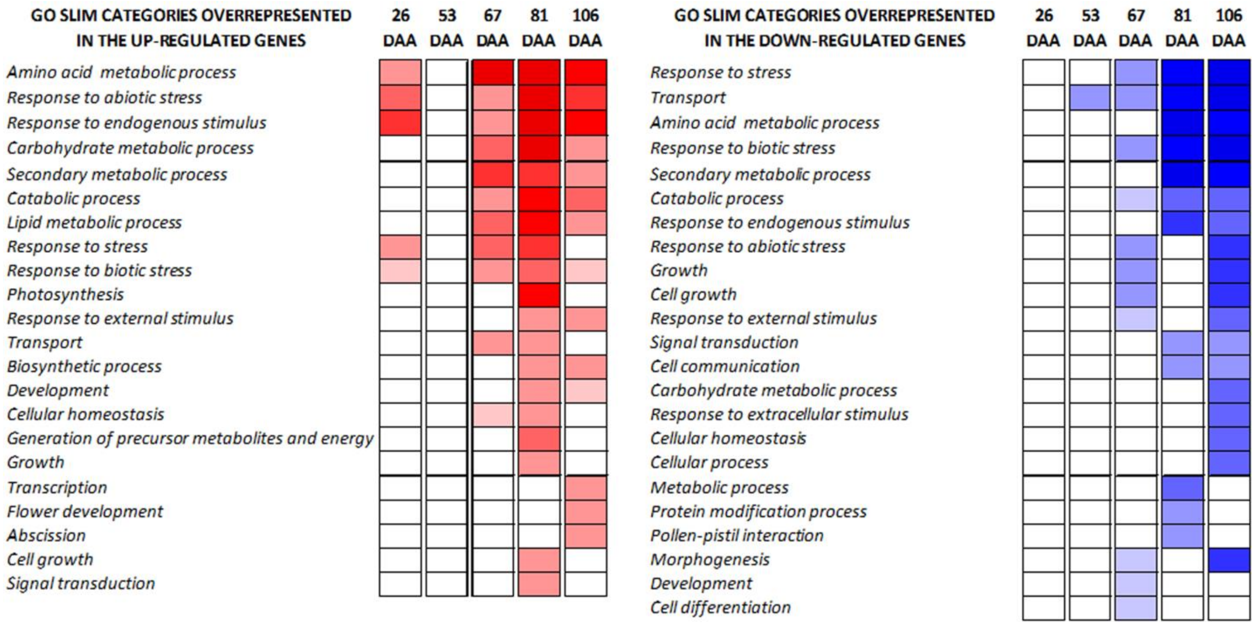

Supplementary FigureS2. Plant gene ontology (GO) slim biological process categories enriched ($P<0.05$) within significantly up- and down-regulated genes under water deficit at 26, 53, 67, 81, and 106 DAA are represented in heatmaps. Red and blue colors denote the overrepresented categories within up- or down-regulated genes, respectively.
