# Supplementary material for: Multi-Omics and Integrated Network Analyses Reveal New Insights into the Systems Relationships between Metabolites, Structural Genes, and Transcriptional Regulators in Developing Grape Berries (Vitis vinifera L.) Exposed to Water Deficit
Source: Front Plant Sci. 2017 Jul 10;8:1124. doi: 10.3389/fpls.2017.01124 (PMC5502274; doi:10.3389/fpls.2017.01124)
Supplement: Supplementary file 9 [file Image_3.PDF]

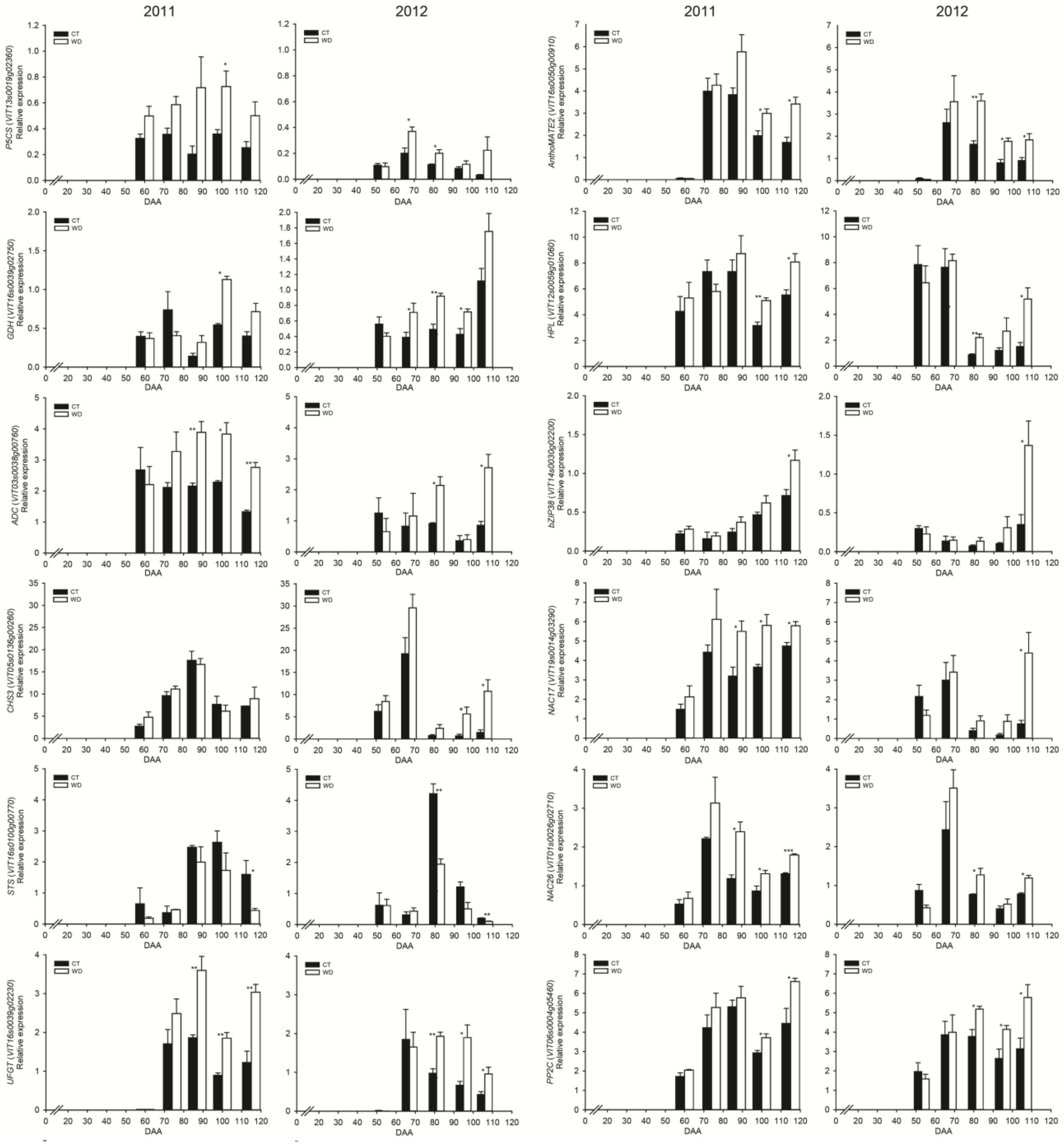

Supplementary FigureS3. Expression (qPCR) of 12 selected genes during berry development in 2011 (left panels) and 2012 (right panels). The expression was analyzed at 60, 74, 87, 100, and 115 DAA in 2011 and at 53, 67, 81, 95, and 106 DAA in 2012. Bars represent ± SE. Asterisks indicate significant differences between treatments at $P < 0.05$ (*), $P<0.01$ (**).
