# Supplementary material for: Multi-Omics and Integrated Network Analyses Reveal New Insights into the Systems Relationships between Metabolites, Structural Genes, and Transcriptional Regulators in Developing Grape Berries (Vitis vinifera L.) Exposed to Water Deficit
Source: Front Plant Sci. 2017 Jul 10;8:1124. doi: 10.3389/fpls.2017.01124 (PMC5502274; doi:10.3389/fpls.2017.01124)
Supplement: Supplementary file 10 [file Image_4.PDF]

Modulation of glycolysis and tricarboxylic acid cycle genes and of sugar related genes under water deficit

Central or primary metabolism fulfils the essential needs of a plant and provides energy for growth, development, and building blocks for the specialized metabolism. The respiratory metabolism is one of them and consists of glycolysis, tricarboxylic acid cycle (TCA), and electron transport chain. Significant differences in the gene expression were observed from 67 DAA onward, and the majority of these genes were up-regulated by water deficit. Interestingly when orthologues displayed a different modulation under water deficit, the ones with higher expression at the specific developmental stage were up-regulated by water deficit. Exceptions were pyruvate kinases; of the three pyruvate kinases modulated by water deficit, the most expressed in the berry (*VIT_16s0050g02660*) was down-regulated during ripening. Moreover, in the gluconeogenesis pathway, two phosphoenolpyruvate carboxykinases (*VIT_05s0049g00950* and *VIT_14s0036g00420*) were down-regulated during berry ripening.

Few genes of the TCA cycle were differently expressed during berry development, and were moderately modulated at 81 or 106 DAA. Consistently with the reduction of malate concentration under water deficit (Fig. 3), three mitochondrial malate dehydrogenases were modulated by WD at 106 DAA, and the two most expressed ones (*VIT_10s0003g01000* and *VIT_17s0000g06270*) were up-regulated.

Sugars are largely accumulated in grape berries and, together with organic acids, are the major osmolytes accumulated in the berry. Sugars can also act as signals modulating the production of specialized metabolites in plants and fruits (Lecourieux *et al.* 2014). In grape berries, two hexoses, glucose and fructose, are largely accumulated in the vacuoles of the fruit cells from the onset of ripening. Among the sugar transporters identified by Afoufa-Bastien *et al.* (2010), *VviSUC27* (*VIT_18s0076g00250*) was up-regulated at 81 DAA by

water deficit, as well the hexose transporter *VviHT3* (*VIT_11s0149g00050*) at 67 and 81 DAA. On the contrary, water deficit down-regulated the expression of other hexose transporters, such as *VviHT1* (*VIT_00s0181g00010*), *VviHT2* (*VIT_18s0001g05570*), *VviHT5* (*VIT_05s0020g03140*), and *VviHT13* (*VIT_11s0016g03400*) during ripening. Several *VviSWEET* genes known to be expressed in the berry (Chong *et al.* 2014) that codify for sugar uniporters, were modulated by WD: *VviSWEET7* (*VIT_02s0025g02080*), *VviSWEET10* (*VIT_17s0000g00830*), and *VviSWEET11* (*VIT_07s0104g01340*) were up-regulated at 53 DAA, 81, and 106 DAA, respectively. However, *VviSWEET15* (*VIT_01s0146g00260*) – the SWEET gene with the highest expression during ripening – was down-regulated at 81 and 106 DAA, as well as *VviSWEET17d* (*VIT_14s0060g01910*) which was down-regulated at 106 DAA. A cell wall invertase (*VIT_09s0002g02320*) was down-regulated in WD at 81 DAA and three cytoplasmic neutral invertases (*VIT_03s0038g01480*, *VIT_05s0077g00510*, and *VIT_13s0074g00720*) were up-regulated during ripening. Two vacuolar invertases were modulated by water deficit: *VviGIN1* (*VIT_16s0022g00670*) was down-regulated at 67 and 81 DAA, while *VviGIN2* (*VIT_02s0154g00090*) was up-regulated at 81 and 106 DAA. Among tonoplast monosaccharide transporters: *VIT_03s0038g03940* was up-regulated while *VIT_18s0122g00850* was down-regulated in WD at late stages of ripening.

Polyols can be involved in the stress response (Lecourieux *et al.*, 2014). Despite the limited effect of water deficit on the concentration of polyols in the berry, polyol-monosaccharide transporters (*VviPTM2 – VIT_11s0016g03920*, and *VviPTM4 – VIT_12s0059g00250*) were differently modulated by WD. *VviPTM2* was up-regulated at 67 and 81 DAA, whereas *VviPTM4* was down-regulated at 81 DAA. Several galactinol synthases were modulated by water deficit: of these genes, the one that had the major expression during fruit ripening (*VIT_01s0127g00470*) was consistently up-regulated from 67 to 106 DAA. Furthermore,

two raffinose synthases (*VviRAFS1* – *VIT_14s0066g00810*, and *VviRAFS2* – *VIT_17s0000g08960*) were also up-regulated during ripening.

References

Afoufa-Bastien D., Medici A., Jeauffre J., Coutos-Thévenot P., Lemoine R., Atanassova R. & Laloi M. (2010) The Vitis vinifera sugar transporter gene family: phylogenetic overview and macroarray expression profiling. *BMC Plant Biology*, **10**, 245.

Chong J., Piron M.C., Meyer S., Merdinoglu D., Bertsch C. & Mestre P. (2014) The SWEET family of sugar transporters in grapevine: VvSWEET4 is involved in the interaction with Botrytis cinerea. *Journal of Experimental Botany*, **65**, 6589-6601.

Lecourieux F., Kappel C., Lecourieux D., Serrano A., Torres E., Arce-Johnson P. & Delrot S. (2014) An update on sugar transport and signalling in grapevine. *Journal of Experimental Botany*, **65**, 821-832.

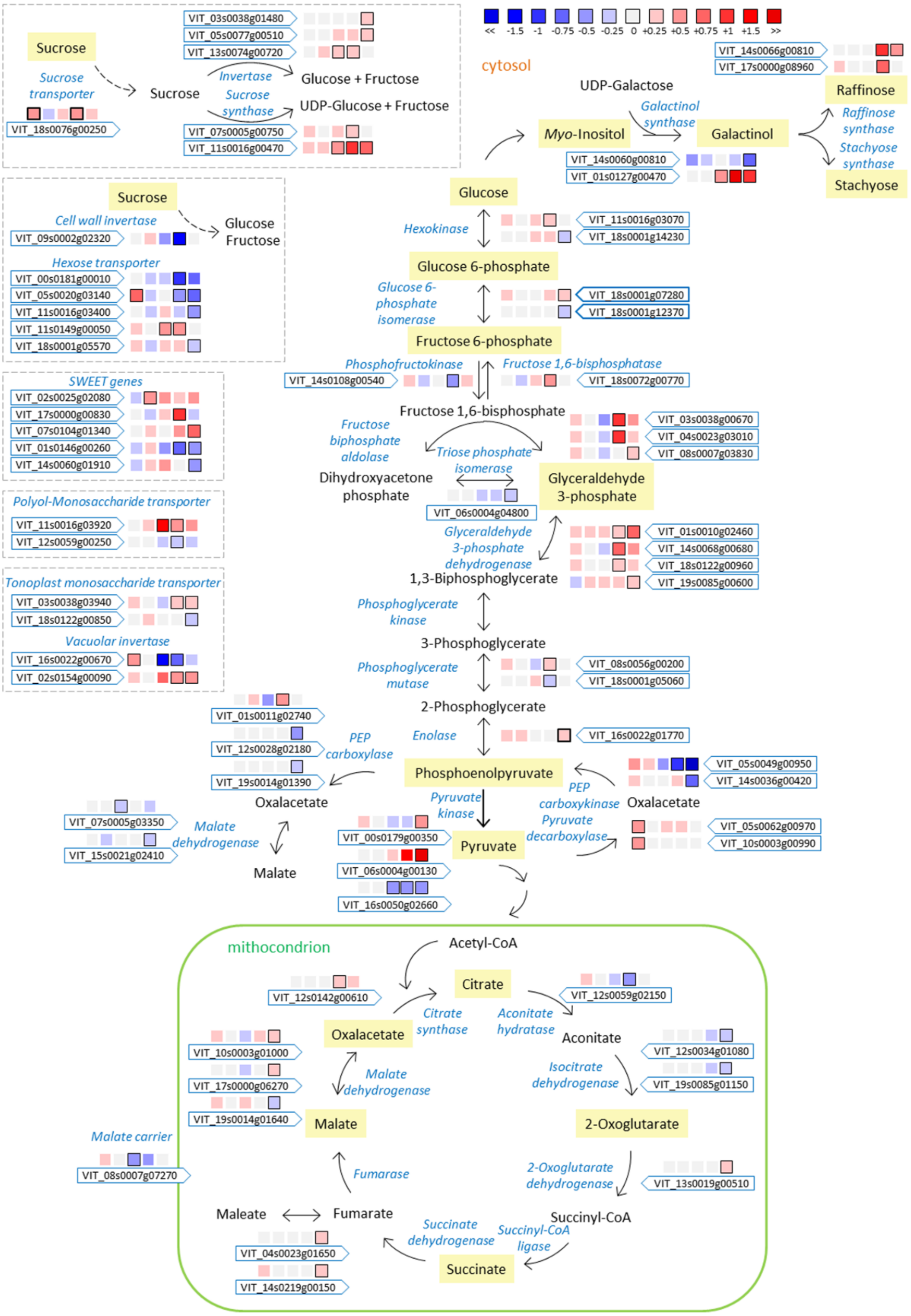

Supplementary FigureS4. Differentially expressed genes codifying for enzymes involved in the glycolysis, the tricarboxylic acid cycle, and the sugar import during fruit development in 2012. Heatmaps represent log_2FC (WD/CT) levels at 26, 53, 67, 81, and 106 DAA from left to right. Blue and red color shades indicate down- or up-regulation of the gene under water deficit, respectively. Bold margins identify significant differences (padj<0.05) between treatments.
