# Supplementary material for: Multi-Omics and Integrated Network Analyses Reveal New Insights into the Systems Relationships between Metabolites, Structural Genes, and Transcriptional Regulators in Developing Grape Berries (Vitis vinifera L.) Exposed to Water Deficit
Source: Front Plant Sci. 2017 Jul 10;8:1124. doi: 10.3389/fpls.2017.01124 (PMC5502274; doi:10.3389/fpls.2017.01124)
Supplement: Supplementary file 11 [file Image_5.PDF]

Modulation of branched-chain amino acids, proline, and polyamines under water deficit

Water deficit increased the concentration of the short branched-chain amino acids leucine, valine, and isoleucine in both seasons (Fig. 3). Four genes involved in valine and leucine biosynthesis were modulated by WD. An acetolactate synthase (*VIT_14s0068g01960*) was down-regulated at 67 and 81 DAA; a ketol-acid reductoisomerase (*VIT_12s0028g02340*) was up-regulated at 81 DAA; a dihydroxy-acid dehydratase (*VIT_05s0051g00830*) was up-regulated at 67, 81, and 106 DAA, and a branched-chain amino acid transaminase (*VIT_14s0128g00100*) was down-regulated at 81 and 106 DAA. The intermediate of the tricarboxylic acid cycle, 2-oxoglutarate, is the precursor for the synthesis of glutamate, a precursor of proline. Two glutamate dehydrogenases (*VviGluDH – VIT_16s0039g02720* and *VIT_16s0039g02750*) and two glutamate synthases (*VviGluS – VIT_08s0007g05260* and *VIT_16s0098g00290*) were up-regulated during the final stages of ripening. Although we could not quantify the glutamate, we observed a large increase of proline concentration. Consistently, a pyrroline-5-carboxylate synthase (*VviP5CS – VIT_13s0019g02360*), a key enzyme for proline synthesis, was strongly up-regulated during the final stages of ripening.

Furthermore, polyamines are derived from the decarboxylation of arginine or ornithine. Our metabolite analysis showed a clear increase in putrescine under water deficit conditions. An arginine decarboxylase (*VIT_03s0038g00760*) – that codifies for a key step of the putrescine biosynthesis – was up-regulated at 67, 81, and 106 DAA. Similarly, a spermine synthase (*VIT_05s0020g03200*) was strongly up-regulated at 67 and 81 DAA.

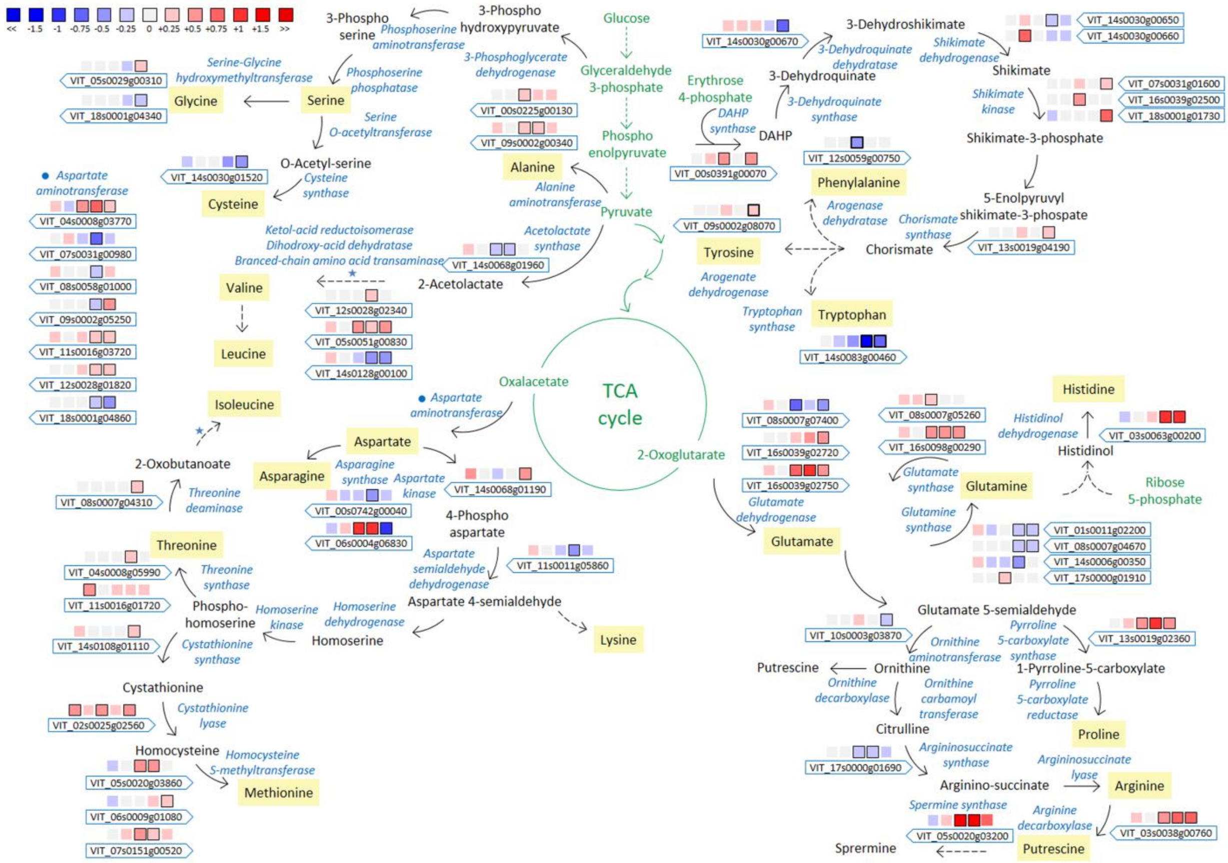

Supplementary FigureS5. Differentially expressed genes codifying for enzymes involved in the amino acid biosynthesis during fruit development in 2012. Heatmaps represent $\log_2$FC (WD/CT) levels at 26, 53, 67, 81, and 106 DAA from left to right. Blue and red color shades indicate down- or up-regulation of the gene under water deficit, respectively. Bold margins identify significant differences (padj<0.05) between treatments. Symbols identify commonly regulated steps of the pathway.
