# Supplementary material for: Multi-Omics and Integrated Network Analyses Reveal New Insights into the Systems Relationships between Metabolites, Structural Genes, and Transcriptional Regulators in Developing Grape Berries (Vitis vinifera L.) Exposed to Water Deficit
Source: Front Plant Sci. 2017 Jul 10;8:1124. doi: 10.3389/fpls.2017.01124 (PMC5502274; doi:10.3389/fpls.2017.01124)
Supplement: Supplementary file 12 [file Image_6.PDF]

Modulation of phenylpropanoid, stilbenoids, and flavonoid pathways under water deficit

Consistently with the differences observed in the phenylpropanoid, stilbenoid, and flavonoid accumulation (Fig. 4), water deficit strongly modulated several steps of the related biosynthetic pathways.

Two phenylalanine ammonia lyases (*VviPAL* – *VIT_06s0004g02620* and *VIT_13s0019g04460*) and one *trans*-cinnamate 4-monooxygenase (*VviC4H* – *VIT_06s0004g08150*) were up-regulated at 67 and 81 DAA. Conversely, other two *VviPALs* (*VIT_08s0040g01710* and *VIT_16s0039g01100*) and another *VviCH4* (*VIT_11s0065g00350*) were down-regulated during ripening. In addition, four 4-coumarate-CoA ligases (*Vvi4CLs*) were modulated at different developmental stages. *VIT_02s0025g03660* was up-regulated while *VIT_06s0061g00450* was down-regulated at 106 DAA; *VIT_11s0052g01090* was up-regulated at 67 DAA and down-regulated at 81 DAA; whereas the *Vvi4CL* most expressed during fruit ripening (*VIT_16s0050g00390)* was up-regulated under water deficit at 67, 81, and 106 DAA. Modifications of the intermediates of the phenylpropanoid pathway produce cinnamic acids. A p-coumaroyl shikimate 3'-hydroxylase (*VviC3H* – *VIT_11s0037g00440*), a hydroxycinnamoyl-CoA:shikimate/quinate hydroxycinnamoyltransferase (*VviHCT* – *VIT_08s0040g00780*), a caffeic acid 3-*O*-metyltransferase (*VviCOMT* – *VIT_02s0025g02920*), and two caffeoyl-CoA 3-*O*-methyltransferases (*VviCCoAOMT* – *VIT_01s0010g03460* and *VIT_07s0031g00350*) were up-regulated in WD berries during ripening. An exception was a *VviCOMT* (*VIT_15s0048g02480*) that was down-regulated at 81 and 106 DAA; however, this gene was detected at very low levels.

Water deficit modulated the expression of many structural genes of the flavonoid pathway. In particular, three chalcone synthases (*VviCHS* – *VIT_05s0136g00260, VIT_14s0068g00920,* and *VIT_14s0068g00930*), two chalcone isomerases (*VviCHI* –

VIT_13s0067g03820 and *VIT_13s0067g02870*), seven flavonoid 3'5'-hydroxylases (*VviF3'5'H – VIT_06s0009g02810, VIT_06s0009g02830, VIT_06s0009g03000, VIT_06s0009g02920, VIT_06s0009g03010, VIT_06s0009g02860,* and *VIT_06s0009g02840*), two flavanone 3-hydroxylases (*VviF3H – VIT_04s0023g03370* and *VIT_18s0001g14310*), one dihydroflavonol reductase (*VviDFR – VIT_18s0001g12800*), one leucoanthocyanidin dioxygenase (*VviLDOX –VIT_02s0025g04720*), two flavonol synthases (*VviFLS – VIT_18s0001g03470, VIT_03s0017g00710*), two flavonol-3-*O*-glycosyltransferases (*VviGT5 – VIT*_11s0052g01600, and *VviGT6 – VIT_11s0052g01630*) (Ono *et al.* 2010), two leucoanthocyanidin reductases (*VviLAR1 – VIT*_01s0011g02960 and *VviLAR2 –VIT_17s0000g04150*), two galloyl glucosyltransferases (*VvigGT1 – VIT*_03s0091g00040 and *VvigGT2 – VIT_03s0180g00200*) putatively involved in PA galloylation (Khater *et al.* 2012), the UDP-glucose:flavonoid-3-*O*-glucosyltransferase (*VviUFGT – VIT_16s0039g02230*), two anthocyanin-*O*-methyltransferases (*VviAOMT – VIT_01s0010g03510* and *VIT_01s0010g03490*) (Fournier-Level *et al.* 2011), and one anthocyanin-3-*O*-glucoside-6''-*O*-acyltransferase (*Vvi3AT – VIT_03s0017g00870*) (Rinaldo *et al.* 2015) were up-regulated by WD at several developmental stages during berry ripening. Few flavonoid genes were down-regulated by water deficit. Specifically, there were a *VviLDOX* (*VIT_08s0105g00380*), one glycosyltransferases putatively involved in PA galloylation (*VviGT3 – VIT_03s0180g00320*) (Khater *et al.*, 2012), and three *VviFLSs* (*VIT_10s0003g02450, VIT_13s0047g00210,* and *VIT_18s0001g03430*).

Once formed, flavonols, proanthocyanidins and anthocyanins are transported into the vacuoles. A proanthocyanidins transporter (*VviPAMATE1 – VIT_12s0028g01160*) (Pérez-Díaz *et al.* 2014), a glycosylated-anthocyanins protein transporter (*VviABCC1 – VIT_16s0050g02480*) (Francisco *et al.* 2013), and an anthocyanin-acylglucoside transporter (*VviAnthoMATE2 – VIT_16s0050g00910*) (Gomez *et al.* 2009) were differently expressed.

VviPAMATE1 was up-regulated at 67 DAA, *VviABCC1* was down-regulated at 67 DAA and up-regulated at 106 DAA, and *VviAnthoMATE2* was up-regulated at 67, 81, and 106 DAA.

The stilbenoid pathway competes with the flavonoid one for precursors. On the contrary than the flavonoid one, this pathway was strongly down-regulated by water deficit. Among the 45 stilbene synthases annotated in the *Vitis vinifera* genome (Vannozzi *et al.* 2012), 28 were down-regulated by WD during ripening. Interestingly, the modulation of the two MYB TF that regulate the stilbenoid biosynthesis – *VviMYB14* and *VviMYB15* – (Höll *et al.* 2013) was not consistent with the modulation of *VviSTSs*. *VviMYB14* was down-regulated at 67 and 81, and up-regulated at 106, while *VviMYB15* was up-regulated at 67 and 81 DAA.

References

Fournier-Level A., Hugueney P., Verriès C., This P. & Ageorges A. (2011) Genetic mechanisms underlying the methylation level of anthocyanins in grape (Vitis vinifera L.). *BMC Plant Biology*, **11**, 179.

Francisco R.M., Regalado A., Ageorges A., Burla B.J., Bassin B., Eisenach C. ...,Nagy R. (2013) ABCC1, an ATP binding cassette protein from grape berry, transports anthocyanidin 3-O-glucosides. *The Plant Cell*, **25**, 1840-1854.

Gomez C., Terrier N., Torregrosa L., Vialet S., Fournier-Level A., Verriès C. ...,Ageorges A. (2009) Grapevine MATE-Type proteins act as vacuolar H+-Dependent acylated anthocyanin transporters. *Plant Physiology*, **150**, 402-415.

Höll J., Vannozzi A., Czemmel S., D'Onofrio C., Walker A.R., Rausch T. ...,Bogs J. (2013) The R2R3-MYB transcription factors MYB14 and MYB15 regulate stilbene biosynthesis in Vitis vinifera. *The Plant Cell*, **25**, 4135-4149.

Khater F., Fournand D., Vialet S., Meudec E., Cheynier V. & Terrier N. (2012) Identification and functional characterization of cDNAs coding for hydroxybenzoate/hydroxycinnamate glucosyltransferases co-expressed with genes related to proanthocyanidin biosynthesis. *Journal of Experimental Botany*, **63**, 1201-1214.

Ono E., Homma Y., Horikawa M., Kunikane-Doi S., Imai H., Takahashi S. ...,Nakayama T. (2010) Functional differentiation of the glycosyltransferases that contribute to the chemical diversity of bioactive flavonol glycosides in grapevines (Vitis vinifera). *The Plant Cell*, **22**, 2856-2871.

Pérez-Díaz R., Ryngajllo M., Pérez-Díaz J., Peña-Cortés H., Casaretto J.A., González-Villanueva E. & Ruiz-Lara S. (2014) VvMATE1 and VvMATE2 encode putative proanthocyanidin transporters expressed during berry development in Vitis vinifera L. *Plant Cell Reports*, **33**, 1147-1159.

Rinaldo A., Cavallini E., Jia Y., Moss S.M.A., McDavid D.A.J., Hooper L.C. ...,Walker A.R. (2015) A grapevine anthocyanin acyltransferase, transcriptionally regulated by

VvMYBA, can produce most acylated anthocyanins present in grape skins. *Plant Physiology*, pp.01255.02015.

Vannozzi A., Dry I.B., Fasoli M., Zenoni S. & Lucchin M. (2012) Genome-wide analysis of the grapevine stilbene synthase multigenic family: genomic organization and expression profiles upon biotic and abiotic stresses. *BMC Plant Biology*, **12**, 130.

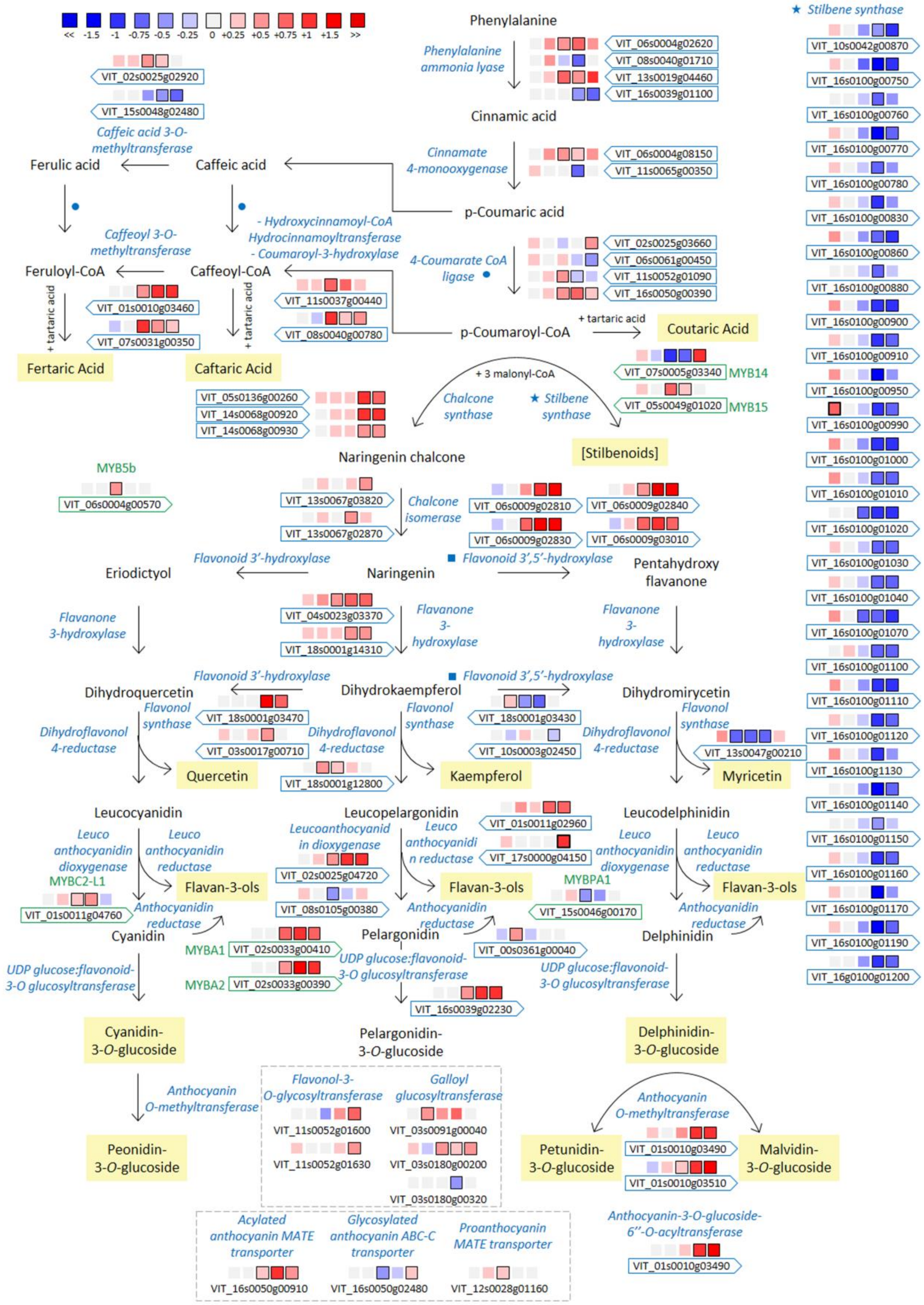

Supplementary FigureS6. Differentially expressed genes codifying for enzymes involved in the phenylpropanoid, flavonoid, and stilbenoid biosynthesis during fruit development in 2012. Heatmaps represent $\log_2$FC (WD/CT) levels at 26, 53, 67, 81, and 106 DAA from left to right. Blue and red color shades indicate down- or up-regulation of the gene under water

deficit, respectively. Bold margins identify significant differences ($padj<0.05$) between treatments. Symbols identify commonly regulated steps of the pathway.
