# Supplementary material for: Multi-Omics and Integrated Network Analyses Reveal New Insights into the Systems Relationships between Metabolites, Structural Genes, and Transcriptional Regulators in Developing Grape Berries (Vitis vinifera L.) Exposed to Water Deficit
Source: Front Plant Sci. 2017 Jul 10;8:1124. doi: 10.3389/fpls.2017.01124 (PMC5502274; doi:10.3389/fpls.2017.01124)
Supplement: Supplementary file 13 [file Image_7.PDF]

Modulation of carotenoid, ABA biosynthetic genes and VOC related genes under water deficit

Water deficit affected the expression of several genes of the carotenoid pathway, mostly by up-regulating them. A phytoene synthase (*VviPSY2 – VIT_12s0028g00960*), a phytoene desaturase (*VviPDH1 –VIT_04s0023g01790*), two ζ-carotene desaturases (*VviZDS – VIT_03s0038g02680* and *VIT_14s0030g01740*), two β-carotene hydroxylases (*VviBCH1 – VIT_02s0025g00240*, a *VviBCH2 – VIT_16s0050g01090*), two violaxanthin de-epoxidases (*VvVDE1 –VIT_*04s0043g01010, and *VvVDE2 – VIT_07s0031g01770*), and a neoxanthin synthase (*VviNSY1 – VIT_14s0006g02880*) were up-regulated during ripening in one or two developmental stages. Exceptions in the pathway were a carotenoid isomerase (*VviCISO1 – VIT_08s0032g00800*) and a zeaxanthin epoxidase (*VviZEP1 – VIT_07s0031g00620*) that were down-regulated.

Both neoxanthin and violaxanthin can be cleaved by 9-*cis*-epoxycarotenoid dioxygenase (NCED) and further modified to produce the drought and ripening related hormone abscisic acid (ABA). Three *VviNCEDs* (*VIT_05s0051g00670*, *VIT_10s0003g03750*, and *VIT_19s0093g00550*) were up-regulated in WD berries during ripening.

Several volatile organic compounds (VOCs) are produced from the peroxidation of free C18 polyunsaturated fatty acids, such as linolenic and linoleic acids. Among the genes involved in this pathway, three 9-lipoxygenases (*Vvi9-LOX – VIT_05s0020g03170*, *VIT_14s0128g00780*, and *VIT_14s0128g00790*) and three 13-lipoxygenases (*Vvi13-LOX – VIT_01s0010g02750*, *VIT_09s0002g01080*, and *VIT_13s0064g01480*) were down-regulated by WD during berry ripening, while a fourth *Vvi13-LOX* (*VIT_06s0004g01510*) was up-regulated at 53 and 106 DAA. Interestingly, subsequent steps of the pathway were consistently induced by water deficit from 67 DAA onwards. A hydroperoxide lyase (*VviHPL – VIT_12s0059g01060*) was up-regulated at 26, 67, 81, and 106 DAA and five

alcohol dehydrogenases (*VviADH* – *VIT_04s0044g01120*, *VIT_04s0044g01130*, *VIT_17s0000g03280*, *VIT_18s0001g15410*, and *VIT_18s0001g15450*) were up-regulated by water deficit during berry ripening. In addition, we identified two grape genes codifying for *(Z)*-3:*(E)*-2-hexenal isomerases (*VviHI* – *VIT_12s0034g01950* and *VIT_12s0034g01870*), based on sequence homology and catalytic site presence to the recently identified paprika and tomato *HI* enzyme (Kunishima *et al.* 2016). Like *VviHPL* and *VviADH*, one *VviHI* (*VIT_12s0034g01950*) was consistently up-regulated to high levels by water deficit in berries from 67 DAA onwards.

Several fruit aromas derive from branched-chain amino acids, such as valine, leucine, and isoleucine, the amino acids that were increased in concentration by water deficit. The conversion of these amino acids into volatile organic compounds such as aldehydes, alcohols, acids, and esters involves the activity of aminotransferases and decarboxylases (Dudareva *et al.* 2013). Recently, Gonda *et al.* (2010) isolated two aminotransferases that catabolite branched-chain amino acids into aroma volatiles. Our differential expression analysis indicated that the grape homologous of these genes were not modulated by water deficit.

References

Dudareva N., Klempien A., Muhlemann J.K. & Kaplan I. (2013) Biosynthesis, function and metabolic engineering of plant volatile organic compounds. *New Phytologist*, **198**, 16-32.

Gonda I., Bar E., Portnoy V., Lev S., Burger J., Schaffer A.A. ...,Lewinsohn E. (2010) Branched-chain and aromatic amino acid catabolism into aroma volatiles in Cucumis melo L. fruit. *Journal of Experimental Botany*, **61**, 1111-1123.

Kunishima M., Yamauchi Y., Mizutani M., Kuse M., Takikawa H. & Sugimoto Y. (2016) Identification of (Z)-3:(E)-2-hexenal isomerases essential to the production of the leaf aldehyde in plants. *Journal of Biological Chemistry*, jbc.M116.726687.

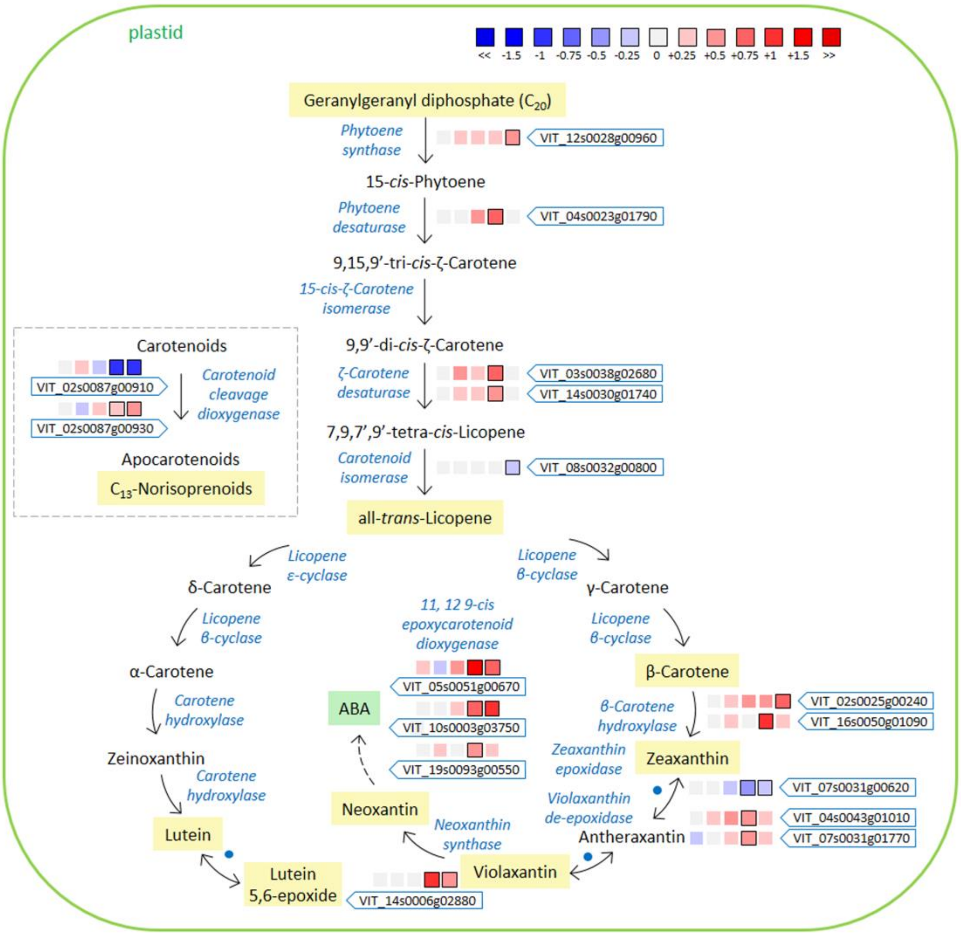

Supplementary FigureS7. Differentially expressed genes codifying for enzymes involved in the carotenoid biosynthesis during fruit development in 2012. Heatmaps represent log_2FC (WD/CT) levels at 26, 53, 67, 81, and 106 DAA from left to right. Blue and red color shades indicate down- or up-regulation of the gene under water deficit, respectively. Bold margins identify significant differences (padj<0.05) between treatments. Symbols identify commonly regulated steps of the pathway.
